# Aminomethylations of electron-deficient compounds—bringing iron photoredox catalysis into play†

**DOI:** 10.1039/d4sc02612h

**Published:** 2024-06-28

**Authors:** Aleksandra Ilic, Benjamin R. Strücker, Catherine E. Johnson, Simon Hainz, Reiner Lomoth, Kenneth Wärnmark

**Affiliations:** a Centre for Analysis and Synthesis (CAS), Department of Chemistry, Lund University SE-22100 Lund Sweden kenneth.warnmark@chem.lu.se; b Department of Chemistry-Ångström Laboratory, Uppsala University SE-75120 Uppsala Sweden reiner.lomoth@kemi.uu.se

## Abstract

The α-functionalisation of *N*-containing compounds is an area of broad interest in synthetic chemistry due to their presence in biologically active substances among others. Visible light-induced generation of nucleophilic α-aminoalkyl radicals as reactive intermediates that can be trapped by electron-deficient alkenes presents an attractive and mild approach to achieve said functionalisation. In this work, [Fe(iii)(phtmeimb)_2_]PF_6_ (phtmeimb = phenyl(tris(3-methylimidazol-2-ylidene))borate), an N-heterocyclic carbene (NHC) complex based on Earth-abundant iron, was used as photoredox catalyst to efficiently drive the formation of α-aminoalkyl radicals from a range of different α-trimethylsilylamines and their subsequent addition to a number of electron-deficient alkenes under green light irradiation. Mechanistic investigations elucidated the different reaction steps of the complete photocatalytic cycle. In terms of yields and substrate scope, we show that [Fe(iii)(phtmeimb)_2_]PF_6_ can compete with noble metal photoredox catalysts, for instance outcompeting archetypal [Ru(bpy)_3_]Cl_2_ under comparable reaction conditions, illustrating that iron photocatalysts can efficiently facilitate photoredox reactions of synthetic value.

## Abstract 2

Electron transfer to the ^2^LMCT state of a photoactive complex based on Earth-abundant iron has been used to drive photocatalytic aminomethylation reactions by generation of α-aminoalkyl radicals and the underlying mechanism has been investigated.

## Graphical abstract

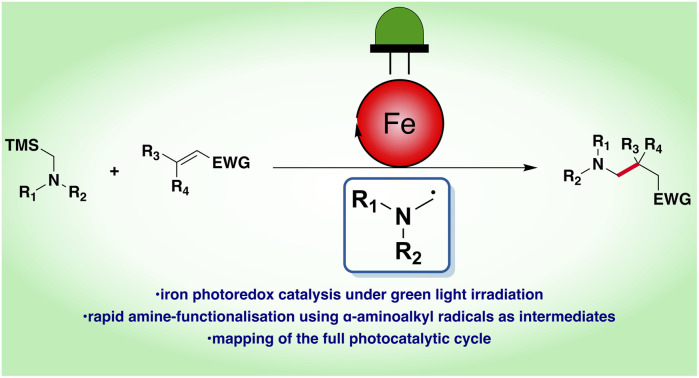

## Introduction

**Scheme 1:**
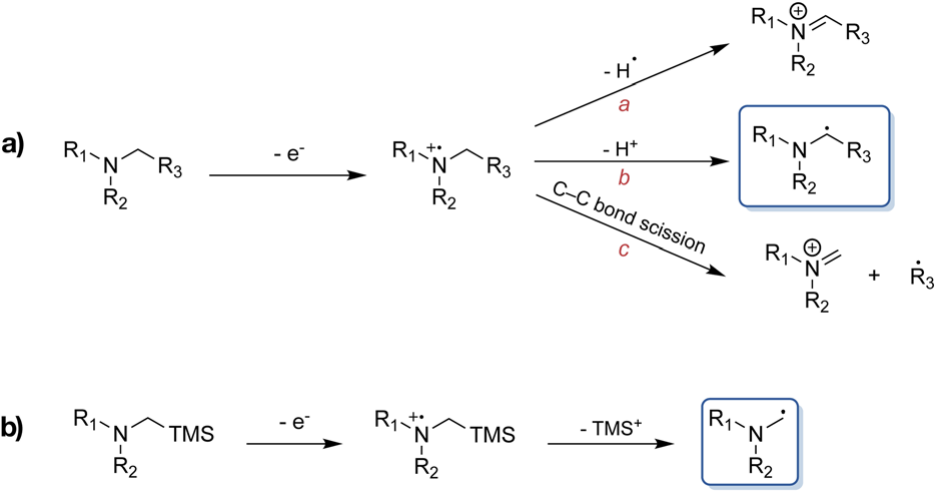
(a) Examples of different reaction pathways of amine radical cations. Pathway a: hydrogen radical abstraction to yield an iminium ion. Pathway b: proton abstraction to afford an α-aminoalkyl radical. Pathway c: C–C bond cleavage resulting in the formation of an iminium ion and an alkyl radical. (b) α-Aminoalkyl radical formation from α-trimethylsilylamines.

Formation of C–C bonds between electron-deficient alkenes and α-aminoalkyl radicals provides a means of rapidly functionalising amines in one step.^1,2^ However, the synthetic utility of α-aminoalkyl radicals as building blocks has not been very extensively explored to date, owing to the fact that these highly reactive nucleophiles tend to undergo further oxidation to the corresponding iminium ions under thermal conditions.^3–5^ Consequently, visible light-mediated photoredox catalysis presents a favoured approach for the generation and use of α-aminoalkyl radicals as ground state reactive intermediates, complementing classical C–N bond formation reactions.^6^ Upon single electron transfer (SET) induced oxidation of an alkylamine, the resulting amine radical cation is known to undergo further reactions, with some of the possible pathways highlighted in Scheme 1a.^7^ Commonly, α-aminoalkyl radicals are accessed through deprotonation of the α-C-atom following amine oxidation *via* SET. Alternatively, they can be afforded through homolytic cleavage of the α-C–H bond by a hydrogen atom transfer catalyst.^8,9^ The nucleophilic radical can then go on to react with the formerly mentioned electrophiles. However, in the presence of multiple α-C–H bonds, regioselectivity-issues can arise. This has led to the exploitation of the favoured fragmentation of *e.g.*, C–Si bonds, where for example α-trialkylsilylamines are used to combat aforementioned issues (Scheme 1b).^10,11^ An additional advantage beyond the improved regioselectivity of the latter is their more facile oxidation (*E*_ox_ ≤ +0.4 *vs.* Fc) compared to the corresponding regular amines.^10^

There have been several reports of α-trialkylsilylamines, which can be easily accessed synthetically, that have been successfully employed as precursors to α-aminoalkyl radicals, generated both by organic as well as noble metal-based photoredox catalysts.^3,6,11–21^ However, the scarcity of the latter along with their requirement for higher energy irradiation due to their absorption maxima commonly being situated in the blue or violet region of the visible light spectrum,^22,23^ have resulted in an increased demand for more sustainable alternatives to the currently still predominantly used ruthenium(ii)^24–28^ and iridium(iii)^28–31^ complexes. Beyond the energetic demands imposed by the necessity of lower wavelength irradiation, unfavourable side reactions caused by excitation of substrates and other reagents through the latter can also occur. While Cu(i) complexes have been used as a more Earth-abundant alternative in photoredox catalysis,^32–37^ and photosensitisers based on Mn(i),^38^ Cr(0) and Cr(iii),^39–42^ Co(iii),^43^ Mo(0)^44^ are on the rise, photoredox catalysts based on iron nevertheless remain attractive candidates and dissociative inner-sphere SET upon ligand-to-metal charge transfer (LMCT) excitation of Fe(iii) currently also receives much attention for its applications in photoredox catalysis.^45–50^ However, so far there are only very few examples of synthetic transformations driven by outer-sphere charge transfer (CT) of iron-based photosensitisers.^48,51^

This can primarily be attributed to the fact that many iron complexes—while being a less expensive, environmentally more benign alternative—have been known to suffer from detrimentally short excited state (ES) lifetimes of their CT states as a result of rapid decay to the ground state (GS) *via* low-lying metal-centred (MC) states.^52–55^

**Scheme 2:**
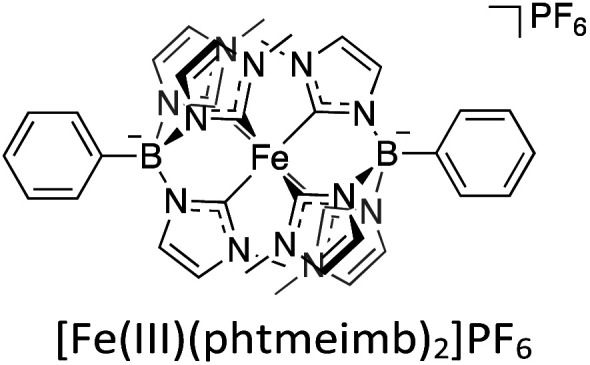
Structure of the iron-based photoredox catalyst used in this study.

Consequently, different strategies have been investigated with the goal of prolonging the CT ES lifetimes of iron-photosensitisers.^56–59^ One very successful approach to extending ES lifetimes of iron-based chromophores has been the introduction of strongly σ-donating N-heterocyclic carbene (NHC) ligands,^60,61^ which results in the MC e_g_-orbitals being destabilized and thus counteracting the rapid deactivation through those states.^54,62,63^ Amongst others, this has brought forth the photoactive compound [Fe(iii)(phtmeimb)_2_]PF_6_ (phtmeimb = phenyltris(3-methylimidazol-2-ylidene)borate) (Scheme 2), a hexa-NHC complex exhibiting room temperature photoluminescence and a ^2^LMCT-lifetime of 2 ns. Owing to the improvement from sub-ps lifetimes to nanoseconds, it was shown that this complex could engage in efficient bimolecular quenching of its ^2^LMCT state. This, along with its favourable GS and ES state redox potentials, makes it a particularly interesting candidate for photocatalytic applications as demonstrated by reports of its use in photoredox catalysis and as photosensitiser in an artificial photosynthesis reaction under green light irradiation.^64–69^ The ^2^LMCT state can act as a strong photooxidant (+0.97 V *vs.* Fc) and is thus capable of oxidising a range of electron donors such as amines, as has previously been studied.^64–66^ The resulting Fe(ii)-GS is moderately reducing at −1.16 V *vs.* Fc.^64^ It has frequently been shown that the catalytic efficiency tends to be limited by low cage escape yields of charge-separated products following the quenching event.^66,68,69^

Here, we employed said iron-based complex as a photoredox catalyst to drive aminomethylations of α,β-unsaturated compounds and other electron-deficient alkenes by formation of α-aminoalkyl radicals from α-trimethylsilylamines under green light irradiation.

Overall, the yields of the reactions under investigation were prevalently fair to good and catalytic efficiencies were comparable to noble metal photoredox catalysts.^3,11,19^ Additionally, we have performed extensive mechanistic investigations to elucidate the underlying mechanisms and give insight into factors which might be limiting the reactivity, opening up for further improvements in the future.

## Results and discussion

### Initial testing and optimisation of reaction conditions

Considering the GS and ES redox potentials and proven ability to engage in efficient bimolecular quenching of its ^2^LMCT state, [Fe(iii)(phtmeimb)_2_]PF_6_ was deemed a promising candidate to effect the desired aminomethylation reactions. Initial attempts at performing the target reaction using regular amines such as triethylamine and *N*-methyldiphenylamine were however not met with success, possibly due to limitations caused by inefficient deprotonation of the amine radical cation generated by the single electron oxidation (see ESI† for further details).^17,70^ This problem could be overcome by the use of α-trimethylsilylamines as precursors to the desired α-aminoalkyl radicals.

**Scheme 3:**
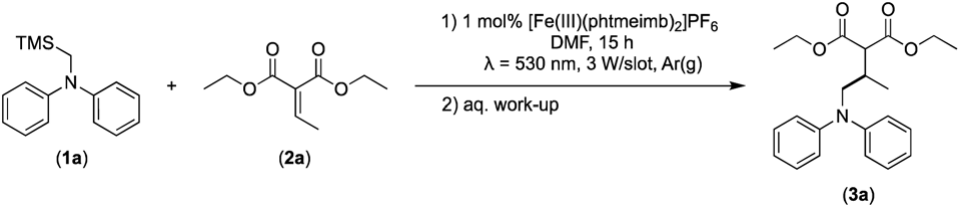
Visible light-driven aminomethylation of electron-deficient alkenes using [Fe(iii)(phtmeimb)_2_]PF_6_ as photoredox catalyst.

The photocatalytic activity of the iron-complex was subsequently investigated using substrate (1a) and diethyl ethylidenemalonate (2a) as the model reaction under irradiation at 530 nm (green light) by a 3 W LED array. It ought to be pointed out that in the case of the aminomethylation of enones, the reaction is likely to first result in the formation of silyl enol ethers, which are however hydrolysed in the work-up step to afford the desired product.^3^ Following optimisations (Fig. S1–S3, ESI†) we found a reliable protocol using mild conditions to afford the desired product (3a) in 15 h using a catalyst loading of 1 mol%, equimolar amounts of (1a) and (2a) and DMF as solvent (Scheme 3).

Control experiments revealed that in the absence of a catalyst or light, as well as when using FeBr_2_ or [Fe(ii)(bpy)_3_](PF_6_)_2_ (bpy = 2,2′-bipyridine) under irradiation, no product (3a) was afforded. When employing [Fe(iii)(btz)_3_](PF_6_)_3_ (btz = 3,3′-dimethyl-1,1′-bis(*p*-tolyl)-4,4′-bis(1,2,3-triazol-5-ylidene)), another Fe-NHC complex that has shown CT state driven photoredox reactivity,^71,72^ as catalyst, only 12% of (3a) were afforded (Table S2, ESI†). The optimised reaction protocol was also employed in an experiment featuring the archetypal [Ru(ii)(bpy)_3_]Cl_2_ as photoredox catalyst under blue light irradiation (*λ* = 450 nm), close to its absorption maximum. [Ru(ii)(bpy)_3_]Cl_2_ has previously been used for similar reactions (in MeCN),^19^ but afforded only 64% of (3a) with our protocol (Table S2, ESI†), which is significantly lower than the yield obtained with our Fe-NHC complex under green light irradiation (92%). The inferior catalytic activity of the former was attributed to rapid photodegradation (Fig. S4, ESI†),^73,74^ which is likely exacerbated by the presence of DMF,^75^ and highlights the advantageous stability of the Fe-NHC complex even under conditions challenging the established noble metal sensitizer.

### Substrate scope

With the optimised reaction conditions in hand, we first set out to explore the applicability of our protocol to a range of α-trimethylsilylamines in a reaction with Michael acceptor (2a) (Table 1). Aromatic amines (1a) as well as (1d–g) could be successfully used to afford the corresponding products (3a) and (3d–g) in fair to good yields, highlighting the opportunity for single-step functionalisation and thus the overall synthetic utility of this reaction. It was noted however, that a somewhat unexpected decrease in yield was observed in the case of product (3e), despite the presumably lower oxidation potential of the corresponding α-trimethylsilylamine. This might be attributed to side reactions such as hydrogen radical abstraction from the methoxy-functionalities. As for product (3d), slight losses in product yield could arise from halogen atom transfer by the *in situ* generated α-aminoalkyl radicals.^76^ Interestingly, for aliphatic amine (1c), the desired product (3c) was obtained only in trace amounts according to LCMS even after extensive efforts towards further optimisations. There, we also observed that after the reaction, only (1c) was still present in appreciable amounts with no distinct side-products in detectable concentrations, indicating decomposition of (2a) over time. Meanwhile (1b), featuring one aliphatic and one aromatic substituent, still afforded the target product (3b) in a yield of 54%. An attempt to employ the secondary amine (1h) as well as its Boc-protected analogue (1i) did not give any product either, likely due to the oxidation of secondary amines and carbamates being more challenging, thus precluding their use in our system.^1^

**Table 1:** Scope and isolated yields of α-trimethylsilylamines used in the visible light-mediated aminomethylation of (2a) under the optimised conditions (1 mol% [Fe(iii)(phtmeimb)_2_]PF_6_, (1a–i) (0.25 mmol), (2a) (0.25 mmol), DMF (2.5 mL))

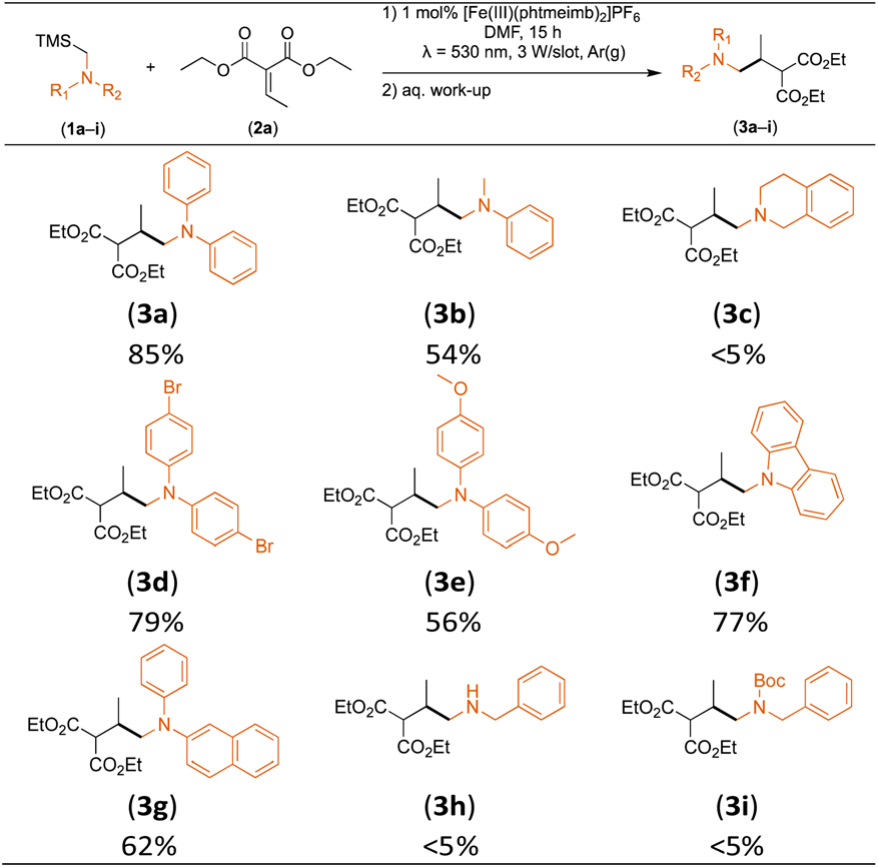

Next, we examined the photocatalytic reactions of α-trimethylsilylamine (1a) with different electron-deficient compounds (2b–f) (Table 2). In the case of benzylidene malononitrile (2b), the desired product (3j) was afforded in good yield (75%). For the substrates (2c–d), yields of the target products (3k–m) were fair, albeit lower than for the model reaction featuring (2a). This can be attributed to the latter being more electron-deficient due to the presence of two electron-withdrawing groups, rendering it more prone to nucleophilic attack by the α-aminoalkyl radical. Further support for this is given by the low yield of 27% for (3n), using substrate (2f), which is structurally similar to (2a) except for an additional electron-donating functionality adjacent to the double bond decreasing its electrophilicity.

**Table 2:** Scope and isolated yields of α,β-unsaturated compounds used in the visible light-mediated aminomethylation using (1a) under the optimised conditions (1 mol% [Fe(iii)(phtmeimb)_2_]PF_6_, (2b–f) (0.25 mmol), (1a) (0.25 mmol), DMF (2.5 mL))

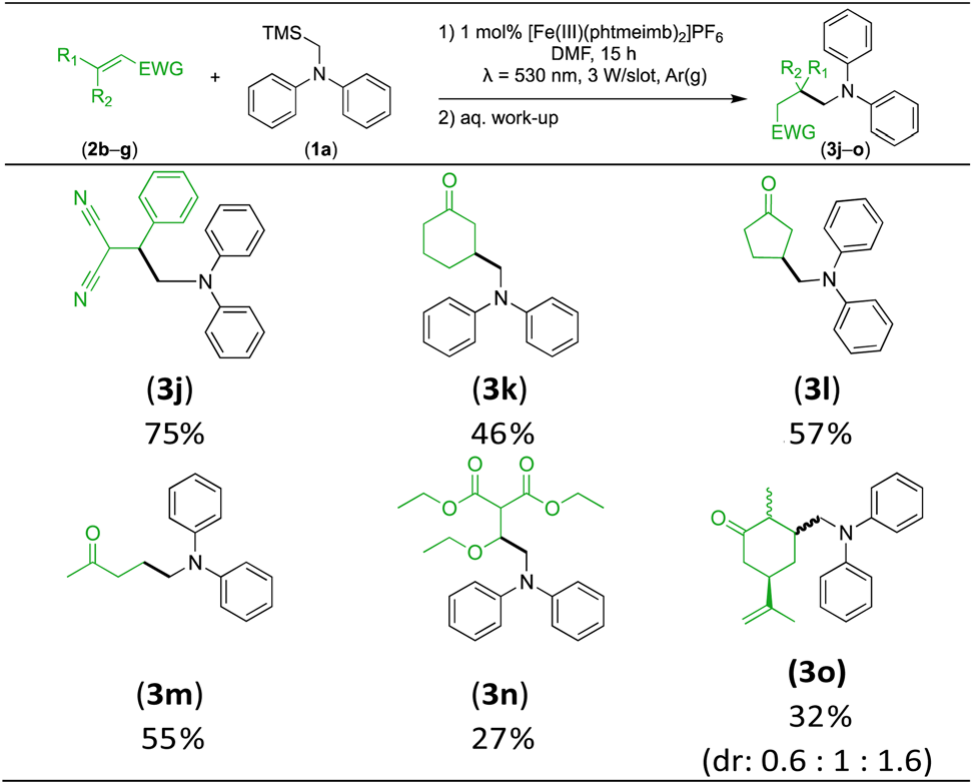

We also investigated *R*-(−)-carvone as substrate, giving rise to a mixture of three diastereomers (3o) in a combined yield of 32%. The decreased yield compared to (3k) can be attributed to the larger steric hindrance, as there was no evidence of addition to the terminal nucleophilic double bond. This also showcases the selectivity of the nucleophilic α-aminoalkyl radical addition.

### Mechanistic investigations

**Scheme 4:**
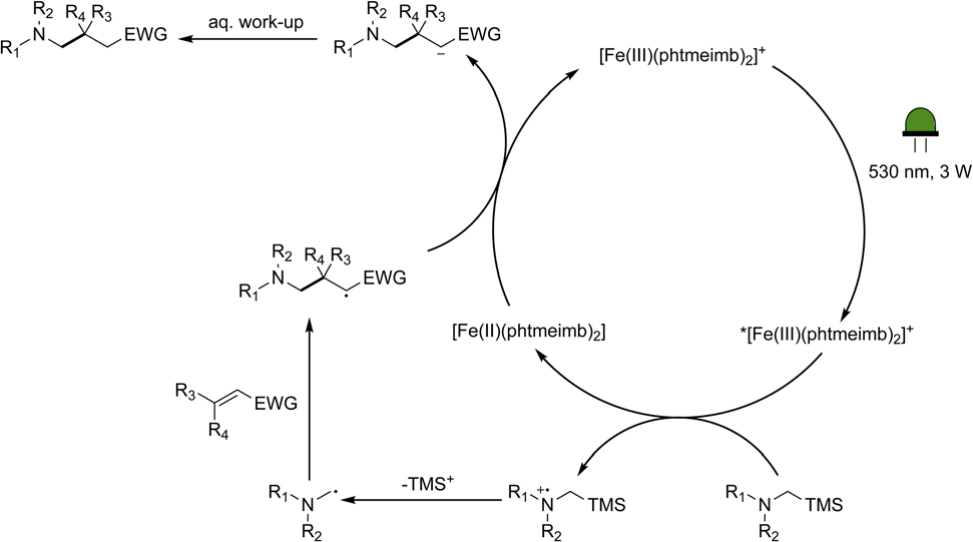
Proposed mechanism for the photocatalytic aminomethylation of α,β-unsaturated compounds using [Fe(iii)(phtmeimb)_2_]PF_6_ as photoredox catalyst.

Following the demonstration of the activity of the iron-based photoredox catalyst in the addition of α-aminoalkyl radicals to α,β-unsaturated compounds, we sought to elucidate the underlying reaction mechanism. In light of the oxidation potentials of α-trialkylsilylamines being sufficiently low (*E*_ox_ ≤ +0.4 *vs.* Fc)^10^ to allow for electron transfer to the ES of our Fe(iii)-complex (+0.97 V *vs.* Fc),^64^ we propose that the reactions under investigation proceed following the mechanism in Scheme 4. The Fe(iii)-complex is excited upon irradiation and the resulting ^2^LMCT state is then reductively quenched by the α-trimethylsilylamine affording the Fe(ii)-GS as well as a radical cation. Through loss of the trimethylsilyl-cation, the desired nucleophilic α-aminoalkyl radical is formed, which can then go on to react with the α,β-unsaturated compound. The newly formed radical is likely to be reduced by the Fe(ii)-GS giving rise to the product and restoring the Fe(iii)-GS. While the reduction potential of the adduct anion is unknown, direct spectroscopic observation of Fe(iii)-recovery (see below) unambiguously demonstrates that reduction of an intermediate formed from the α-aminoalkyl radical and the electron-deficient alkene by the catalyst's Fe(ii)-state (−1.16 V *vs.* Fc in MeCN) is thermodynamically feasible.

**Fig. 1:**
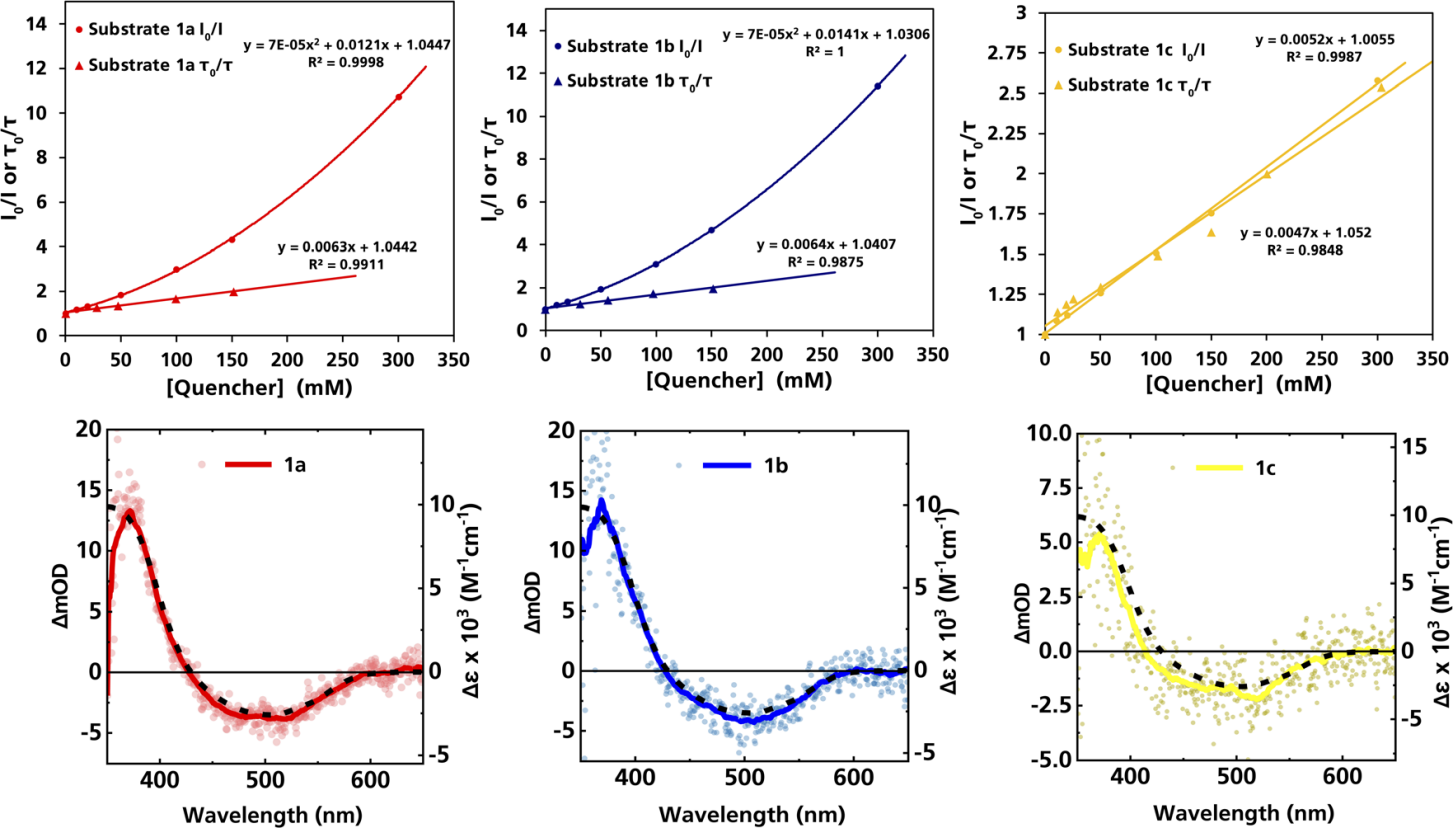
Top row: Stern–Volmer plots derived from the steady-state and time-resolved emission quenching of [Fe(iii)(phtmeimb)_2_]^+^ (0.16 mM) in DMF with substrates (1a–c) with the corresponding emission spectra and TCSPC traces shown in the ESI.† Bottom row: ns-TAS spectra of [Fe(iii)(phtmeimb)_2_]^+^ (0.15 mM) and substrates (1a–c) (104, 101, 88 mM, respectively) in DMF upon 465 nm (30 μs delay, 21.4 ± 0.1 mJ per pulse) excitation (data smoothed by adjacent averaging method) and differential absorption spectrum of the metal-centred reduction based on spectroelectrochemistry in MeCN (---).

The formation of radical species in the reaction was evidenced by the fact that addition of TEMPO (=(2,2,6,6-tetramethylpiperidin-1-yl)oxyl) led to only traces of product being formed in the model reaction and adduct-formation between TEMPO and the α-aminoalkyl radical was observed (Scheme S1, ESI†). With this clear indication that an α-aminoalkyl radical is generated as a result of a visible light-driven reaction, we set out to investigate the proposed first reaction step—the reductive quenching of the ^2^LMCT state leading to oxidation of the α-trimethylsilylamine. The reaction of the excited photoredox catalyst with α-trimethylsilylamines (1a–c) was evidenced by fluorescence quenching (Fig. 1, top row), while any ES reaction with the model Michael acceptor (2a) could be excluded (Fig. S7, ESI†).

For (1a) and (1b), upwards curvatures from the Stern–Volmer plots of the steady-state emission quenching were observed, indicating combinations of both dynamic (diffusion-controlled) and static quenching (association between the quencher and the PS GS, see ESI eqn (1)–(3)† for calculations and Table 3 for association constants). The dynamic quenching rate constants *k*_q_ were independently determined by time-correlated single photon counting (TCSPC) and were in good agreement with the steady-state results (Fig. S9, S11, S13 and S14, ESI†). Although the values of *k*_q_ were similar to previously reported rates for trialkylamines and approximately one order of magnitude slower than results obtained for aromatic amines,^66^ with *k*_q_ = 3.7 × 10^9^ M^−1^ s^−1^ for (1a) and *k*_q_ = 4.0 × 10^9^ M^−1^ s^−1^ for (1b), the combination of both pathways results in higher quenching yields than previously observed (Table 3). For (1c), only diffusion-controlled quenching is observed, with the dynamic quenching rate constant (*k*_q_ = 2.8 × 10^9^ M^−1^ s^−1^) being the slowest of the three. This lower reactivity is tentatively attributed to the presumably higher potential for oxidation of the aliphatic amine. Nevertheless, under the conditions employed in the photocatalysis reactions, efficient quenching, where close to 40% of the excited photocatalyst molecules are quenched, is ensured even for the slowest quencher (1c) (Table 3).

**Table 3:** Excited state quenching data for [Fe(iii)(phtmeimb)_2_]PF_6_ in DMF. (*K*_S_ = static Stern–Volmer quenching constant, *K*_D_ = dynamic Stern–Volmer quenching constant, *k*_q_ = bimolecular quenching rate constant, *η*_q_ = quenching yield, *η*_ce_ = cage escape yield, *η*_q_*η*_ce_ = quantum yield). CEYs calculated under the assumption that the extinction coefficients of Fe(iii) and Fe(ii) in DMF are similar to those in acetonitrile (see details of calculations in ESI)

[Fe(phtmeimb)_2_]PF_6_*τ*_0_ = 1.6 ns (in DMF)						
Quencher	*K*_S_ (M^−1^)	*K*_D_ (M^−1^)	*k*_q_ (10^9^ M^−1^ s^−1^)	*η*_q_ ([Q]/M)	*η*_ce_	*η*_q_ *η*_ce_ ([Q]/M)
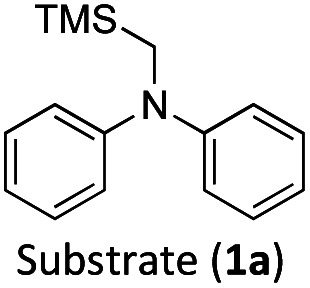	9.9	6.3	3.7	0.67 (0.1)	0.15	0.10 (0.1)
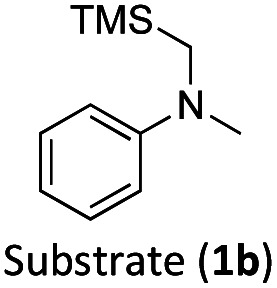	11.3	6.4	4.0	0.68 (0.1)	0.22	0.15 (0.1)
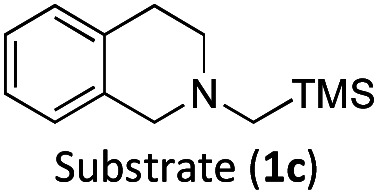	n.a.	5.2	2.8	0.34 (0.1)	0.09	0.03 (0.1)
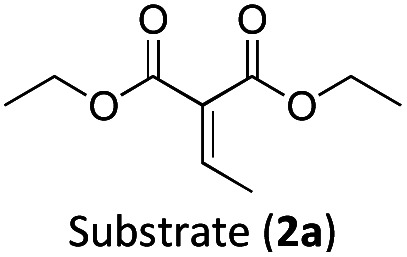	n.a.	n.a.	n.a.	0 (≤0.3)	n.a.	0 (≤0.3)

The products of the quenching reaction were characterised by nanosecond transient absorption spectroscopy (ns-TAS) that monitored the absorption changes induced by excitation at 465 nm (Fig. 1, bottom row). With the short ES lifetime, the quenching reaction is completed within the time resolution (∼10 ns) and the resulting TA-spectra at later time scales (30 μs) show a pronounced product absorption below 400 nm together with a weak bleach centred at 500 nm. These features can be attributed to the Fe(ii)-GS and the Fe(iii)-GS, respectively, which is consistent with reductive quenching of the ES of [Fe(iii)(phtmeimb)_2_]^+^. The TA spectra on the microsecond timescale are in all cases in good agreement with the differential spectrum for the reduction of the complex obtained from spectroelectrochemistry,^64^ while no obvious contributions from the quencher radicals were observed in the spectral range of the TA measurements. TA spectra on the nanosecond timescale however revealed contributions from the short-lived radical cations of the quencher (see below).

The TA signature of the reduced photoredox catalyst remains unchanged over several milliseconds which demonstrates that the α-trimethylsilylamines behave as exemplary sacrificial electron donors without any bimolecular charge recombination between the reduced photoredox catalyst and the oxidised quenchers or their fragments. Quantification of the Fe(ii)-product from its transient absorption and the number of absorbed photons from comparative actinometry (Table S3 and Fig. S17, ESI†) results in cage escape yields (CEYs) of the electron transfer products of 9–22% (Table 3), which is higher than previously observed for the quenching of the same photoredox catalyst by tertiary amines in the same solvent, DMF.^66^ The high CEY of 22% for (1b) was somewhat counterintuitive to the product yield of the reaction, which was significantly lower than for (1a) (Table 1), where a CEY of 15% was determined. It is therefore evident that the different product yields for (1a) and (1b) must be attributed to the efficiency of subsequent steps in the catalytic cycle and their potential competition with unproductive side reactions. Specifically, it is possible that instead of undergoing the desired Michael addition, protodesilylation occurs, which would prohibit product formation as has previously been observed in similar studies.^19^

**Fig. 2:**
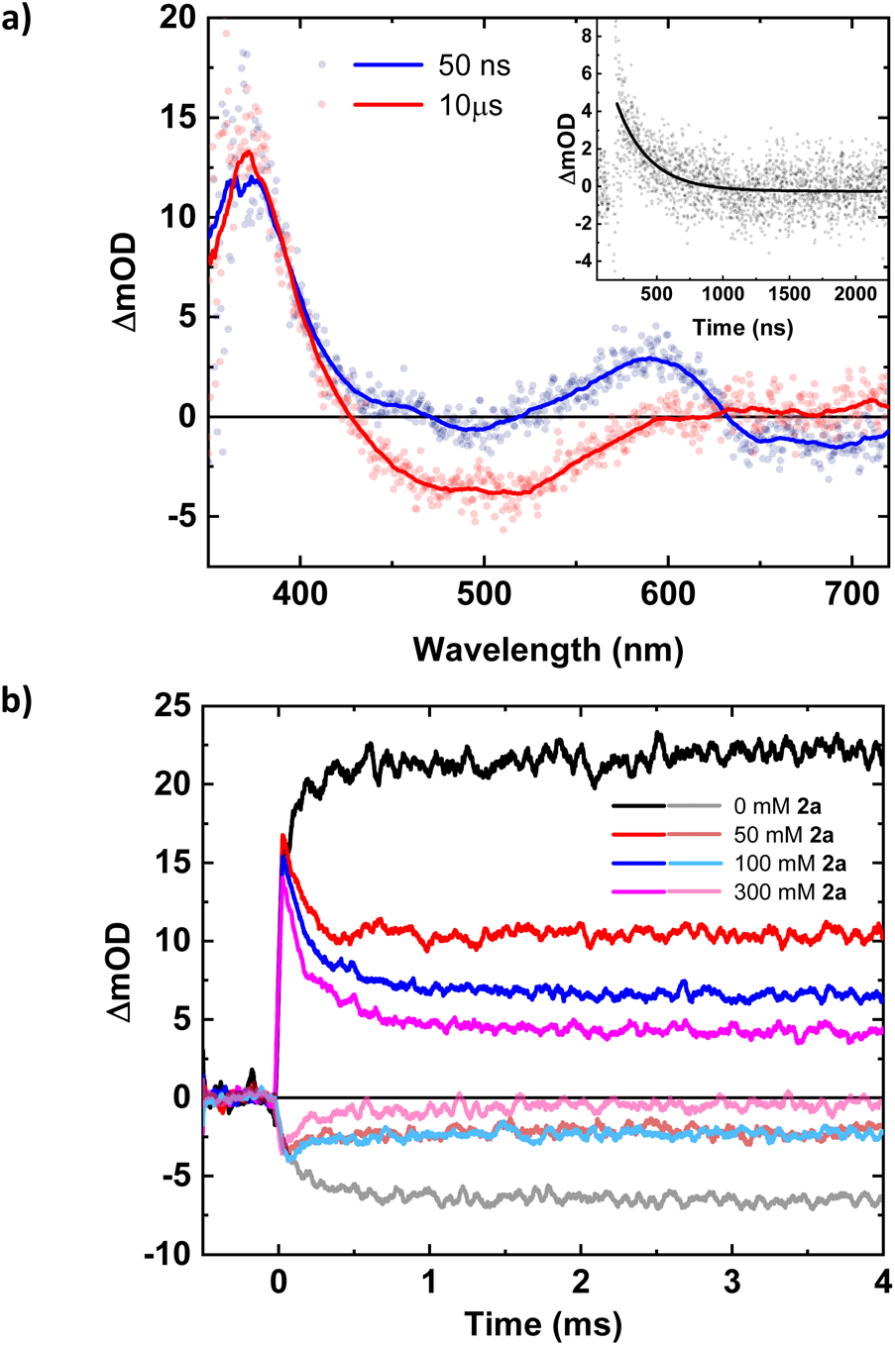
(a) ns-TAS spectra at different time delays of [Fe(iii)(phtmeimb)_2_]^+^ (0.16 mM) and substrate (**1a**) (100 mM). Inset: kinetic trace probed at 600 nm and single exponential fit (*τ* = (244 ± 13) ns). (b) ns-TAS kinetic traces at 375 nm (positive ΔOD) and 500 nm (negative ΔOD) of [Fe(iii)(phtmeimb)_2_]^+^ (0.16 mM) and substrate (1a) (100 mM) with varying concentrations of (2a). All data were collected with excitation at 500 nm (20.0 ± 0.2 mJ per pulse).

To obtain information on the catalytic cycle beyond the initial ES electron transfer step, additional flash photolysis experiments were performed with (1a) with excitation at 500 nm. TA spectra at early time scales (<1 μs) reveal contributions from the oxidized quencher at around 600 nm next to the expected features of the reduced photoredox catalyst at 375 nm (Fig. 2a). The peak at 600 nm decays with a lifetime of 244 ns (Fig. 2a, inset), leaving behind only the Fe(ii)-absorption peak at around 375 nm and the Fe(iii)-ground state bleach (GSB) at 500 nm (Fig. 2a). Consequently, the absorption peak at 600 nm together with additional absorption initially cancelling the GSB around 500 nm could be attributed to the amine radical cation, which then loses its trimethylsilyl-group, generating the desired α-aminoalkyl radical which in turn does not significantly contribute to the transient absorption spectrum. The 244 ns lifetime of the initially observed oxidation product supports the assignment of its decay to an intramolecular process as sub-μs reactions between photoproducts are precluded by their concentrations on the order of 10^−6^ M.

TA data on the millisecond time scale revealed a delayed rise of the 375 nm absorption and a corresponding ∼130 μs component in the GSB at 500 nm (Fig. 2b). Consistent with an additional slow formation of the Fe(ii)-GS, the process is attributed to the reducing α-aminoalkyl radical that might react with the excess of Fe(iii)-GS. This would account for a doubling of the Fe(ii)-yield as long as the radical does not engage in competing reactions. With the photoredox catalyst concentration employed (0.16 mM), the observed kinetics would correspond to a pseudo-first order reaction with a bimolecular rate constant of around 5 × 10^7^ M^−1^ s^−1^. Under catalytic conditions this unproductive reaction of the α-aminoalkyl radical would compete with its desired addition to the Michael acceptor. We therefore investigated the reaction of the photoproducts obtained with quencher (1a) in presence of the Michael acceptor (2a). The TA data (Fig. S18, ESI†) reveals no additional spectral or kinetic features that could be attributed to the expected adduct radical or the product of its reduction. The reactivity of (2a) towards the α-aminoalkyl radical was however clearly evidenced by the observed turnover that could be monitored based on the consumption of reduced photoredox catalyst and the concomitant recovery of its original Fe(iii)-GS. The corresponding kinetic traces at 375 and 500 nm, respectively, reveal two effects of the added Michael acceptor as shown in Fig. 2b: (i) A major part of the previously persistent Fe(ii)-absorption now decays within 0.5 ms and a corresponding part of the Fe(iii)-bleach recovers with the same kinetics. This effect can be attributed to the turnover of the catalyst due to electron transfer to the adduct radical. (ii) A remaining, persistent portion of the TA signal corresponds to the reduced photoredox catalyst formed by the slow reaction with α-aminoalkyl radicals. For this portion of the catalyst, turnover is prevented by the lack of adduct radicals and the magnitude of this portion decreases expectedly with increasing concentration of the Michael acceptor. Efficient competition of the radical addition reaction with the unproductive electron transfer to the Fe(iii) GS requires however concentrations of (2a) on the order of 0.1 M corresponding to a bimolecular rate constant on the order of 10^4^ M^−1^ s^−1^ for the pseudo-first order reaction. The radical addition step led to consumption of Fe(ii) and recovery of Fe(iii) without any delay, and turnover is thus not limited by the final electron transfer step under these conditions. To keep up with the radical addition step, the second order reaction between the adduct radical and the reduced photoredox catalyst, both at comparable concentrations on the order of 10^−6^ M, would have to proceed with a rate constant between 10^9^ and 10^10^ M^−1^ s^−1^, *i.e.* well below the limit given by diffusion of the reactants. With the much lower photon flux in the photoreactor compared to the laser flash photolysis experiments, the concentration of photo-generated species will however be correspondingly lower and the final electron transfer step between the adduct radical and the reduced photoredox catalyst can be expected to become rate-limiting rather than the preceding pseudo-first order reaction with the Michael acceptor. The higher photoredox catalyst concentration used for the synthetic catalytic reactions (1 mM instead of 0.16 mM) will also result in a correspondingly faster reaction with the α-aminoalkyl radicals. The resulting depletion of Fe(iii) would however in turn benefit the desired radical addition.

## Conclusions

Here, we have demonstrated that the Fe-NHC complex [Fe(phtmeimb)_2_]PF_6_ can drive aminomethylation reactions of electron-deficient alkenes *via* photoredox catalysis using green light, thus giving facile access to highly functionalised N-containing compounds, which are of great value, for instance in the pharmaceutical industry. Aromatic amines gave prevalently good yields for the synthetic reactions under investigation, which coincided well with the high yields of the electron-transfer products obtained from the reductive quenching step. However, in the case of aliphatic amine quenchers, no significant product formation was observed despite the appreciable quantum yield of the reduced photocatalyst. There, side reactions such as protodesilylation can occur, resulting in undesired trapping of the α-aminoalkyl radical and subsequent deactivation—an issue that has also been observed in some other reaction systems using organic or noble metal-based photoredox catalysts. Electron-deficient Michael acceptors, such as diethyl ethylidenemalonate afforded the highest yields in our reaction system, but less electrophilic alkenes were also suitable for trapping the highly nucleophilic α-aminoalkyl radicals generated *in situ*. As for the α-trimethylsilylamines, different substituents such as bromo- or methoxy-functionalities were well-tolerated as well as expansion of the aromatic system. Particularly, the good yields obtained with the bromo-substituted precursor for the α-aminoalkyl radical are interesting, since the halide-moieties can act as a handle for further functionalisation.

Through mechanistic investigations, we were able to show that the ^2^LMCT state of the iron photocatalyst is efficiently reduced by α-trialkylsilylamines in a combination of collisional and static quenching and that the products escape geminate recombination with cage escape yields (up to 22%) that compare favourably against the more moderate values often encountered with Fe(iii)-NHC complexes and are on the same order of magnitude as typically found for other photosensitizers.

Beyond that, the formation of the neutral α-aminoalkyl radical and its subsequent addition to a Michael acceptor leading to turnover of the catalyst were monitored spectroscopically for one of the aromatic α-trialkylsilylamines, which enabled us to map the kinetics of the entire catalytic cycle. Such complete studies of photocatalytic cycles are severely underutilised and can afford valuable insights into how photoredox reactions can be further improved. Furthermore, we demonstrated that α-trialkylsilylamines are good quenchers for the Fe(iii)-NHC complex under investigation, which could also be interesting for applications where efficient sacrificial electron donors are needed and the targeted formation of another reducing species, namely α-aminoalkyl radicals, is beneficial, such as in artificial photosynthesis reactions.

This study of the generation and application of α-aminoalkyl radicals as synthetic building blocks expands the currently still rather small range of photoredox catalytic transformations being driven by bimolecular quenching of a CT state of a complex based on Earth-abundant iron, adding to the even smaller number of synthetically useful reactions performed using these systems.

When benchmarking against the archetypal [Ru(bpy)_3_]Cl_2_ using the same reaction conditions and blue light irradiation, we even observed that [Fe(iii)(phtmeimb)_2_]PF_6_ exhibited superior catalytic activity owing to its much higher photostability. Overall, the yields and substrate scope of the herein investigated system showcase that iron photocatalysts can compete with the more established noble metal-based photoredox catalysis, thus contributing to positioning this upcoming field as a viable alternative to the latter.

In light of all these factors, further explorations of synthetically valuable reactions utilising α-aminoalkyl radicals generated by iron photoredox catalysis, *e.g.*, homolytic aromatic substitutions of heterocyclic compounds, are currently under way.

## Data availability

All experimental data is provided in the ESI.†

## Author contributions

A. I. conceived and planned the research, conducted initial testing, substrate syntheses as well as steady-state emission quenching studies, performed data analysis, contributed to the mechanistic interpretation, wrote major parts of the entire manuscript and coordinated the writing. B. R. S. carried out optimisation reactions, control experiments, radical trapping experiments as well as the photocatalytic reactions and product isolations for the scope of α-trimethylsilylamines and contributed to reviewing the manuscript. C. E. J. performed all measurements based on transient absorption and time-resolved emission spectroscopy, determined excited state lifetimes and yields of excited state reactions, and contributed to the mechanistic interpretation, data analysis and the writing of the manuscript. S. H. carried out the photocatalytic reactions and product isolations for the scope of electron-deficient alkenes and contributed to reviewing the manuscript. R. L. devised the spectroscopic reactivity studies, guided the mechanistic interpretation and contributed to the writing of the manuscript. K. W. contributed to the planning of the research and to the writing of the manuscript.

## Conflicts of interest

There are no conflicts to declare.

## Supplementary Material

SC-015-D4SC02612H-s001
