# Supplementary material for: Aminomethylations of electron-deficient compounds—bringing iron photoredox catalysis into play
Source: Chem Sci. 2024 Jun 28;15(30):12077–85. doi: 10.1039/d4sc02612h (PMC11290444; doi:10.1039/d4sc02612h)
Supplement: SC-015-D4SC02612H-s001 [file SC-015-D4SC02612H-s001.pdf]

Electronic Supplementary Material (ESI) for Chemical Science.
This journal is © The Royal Society of Chemistry 2024

Supporting information

Aminomethylations of Electron-Deficient Compounds—Bringing Iron Photoredox Catalysis into Play

Aleksandra Ilic[a], Benjamin R. Strücker[a], Catherine E. Johnson[b], Simon Hainz[a], Reiner Lomoth[b]*, Kenneth Wärnmark[a]*

[a]Centre for Analysis and Synthesis (CAS), Department of Chemistry, Lund University, SE-22100 Lund, Sweden;
[b]Department of Chemistry–Ångström Laboratory, Uppsala University, SE-75120 Uppsala, Sweden.

***Corresponding Author**

E-mail: reiner.lomoth@kemi.uu.se
E-mail: kenneth.warnmark@chem.lu.se

Table of Contents

Aminomethylations of Electron-Deficient Compounds—Bringing Iron Photoredox Catalysis into Play **.....1**

General Information **.....4**

Materials and Instruments.....4

Synthesis of α-Trimethylsilylamines **.....6**

***N*-Phenyl-*N*-((trimethylsilyl)methyl)aniline (1a) 7**

***N*-Methyl-*N*-((trimethylsilyl)methyl)aniline (1b).....7**

***N*-Phenyl-*N*-((trimethylsilyl)methyl)aniline (1c).....8**

4-Bromo-*N*-(4-bromophenyl)-*N*-((trimethylsilyl)methyl)aniline (1d).....9

4-Methoxy-*N*-(4-methoxyphenyl)-*N*-((trimethylsilyl)methyl)aniline (1e) 9

9-((Trimethylsilyl)methyl)-9*H*-carbazole (1f).....10

9-((Trimethylsilyl)methyl)-9*H*-carbazole (1g) 11

***N*-benzyl-1-(trimethylsilyl)methanamine (1h) 11**

***Tert*-butylbenzyl((trimethylsilyl)methyl)carbamate (1i).....12**

Visible light-mediated addition of α-amino alkyl radicals to electron-poor double bonds via reductive quenching of [Fe(phtmeimb)$_2$]PF$_6$ **.....13**

General Procedure for the Photocatalytic Aminomethylation of Electron-Deficient Systems.....13

Optimisations of the reaction conditions 13

Control Experiments 14

Optimised Procedure for the Photocatalytic Aminomethylation of Electron-Deficient Systems.14

Scope of different TMS-methylsilanes using the Optimised Procedure.....15

Diethyl 2-(1-(diphenylamino)propan-2-yl)malonate (**3a**) 15

Diethyl 2-(1-(methyl(phenyl)amino)propan-2-yl)malonate (**3b**).....15

Diethyl 2-(1-(bis(4-bromophenyl)amino)propan-2-yl)malonate (**3d**) 16

Diethyl 2-(1-(bis(4-methoxyphenyl)amino)propan-2-yl)malonate (**3e**) 17

Diethyl 2-(1-(9*H*-carbazol-9-yl)propan-2-yl)malonate (**3f**) 17

Diethyl 2-(1-(naphthalen-2-yl(phenyl)amino)propan-2-yl)malonate (**3g**).....18

Scope of different Michael Acceptors using the Optimised Procedure **18**
2-(2-(Diphenylamino)-1-phenylethyl)malononitrile 18
3-((Diphenylamino)methyl)cyclohexan-1-one (**3k**) 19
3-((Diphenylamino)methyl)cyclopentan-1-one (**3l**) 19
5-(Diphenylamino)pentan-2-one (**3m**) 20
Diethyl 2-(2-(diphenylamino)-1-ethoxyethyl)malonate (**3n**) 20
(5*R*)-3-((diphenylamino)methyl)-2-methyl-5-(prop-1-en-2-yl)cyclohexan-1-one (**3o**) 21
Mechanistic Investigations ***23***
The effect of radical scavengers **23**
Photodegradation Studies **23**
Stern–Volmer quenching studies **24**
Nanosecond transient absorption measurements ***30***
Cage Escape Yield Measurements **30**
Recovery of [Fe(III)(phtmeimb)$_2$]PF$_6$ **32**
NMR Spectra ***33***
References ***54***

General Information

Materials and Instruments

Materials. All solvents used in work-up procedures, for silica gel column chromatography, and/or for purification were obtained from commercial suppliers and used without further purification. Reagents *i.e.,* commercially available amines, methylsilylation reagents, electron-deficient substrates and additives, $[Ru(bpy)_3]Cl_2{\bullet}6H_2O$ as well as deuterated solvents for NMR-spectroscopy were purchased from Sigma-Aldrich or Acros Organics and used without further purification. $[Fe(III)(phtmeimb)_2]PF_6$,[1] $[Fe(btz)_3](PF_6)_3$,[2] and $[Fe(bpy)_3](PF_6)_2$[3] were prepared according to literature protocol.

Photoreactions. Generally, photoreactions were performed in a TAK120 AC photoreactor purchased from HK Testsysteme GmbH. The irradiation was performed using the green LED array (λ=530 nm, 3.03 W/vial) in 6 mL clear glass crimp top vials with septum. The temperature during reaction was maintained at 27–30 °C using air cooling.

Chromatography. Precoated Merck silica gel 60 F254 plates were used for thin-layer-chromatography (TLC) analysis and products were visualised using UV light or ethanolic $KMnO_4$ solution and heat as the developing agent. Flash silica gel chromatography was performed using Merck silica gel (pore size 60 Å, 230–400 mesh particle size, particle size 0.043-0.063 mm).

Characterisation. NMR spectra were recorded at ambient temperature on a BrukerAvance II 400 MHz NMR spectrometer (400/101 MHz $^1H/^{13}C$). Chemical shifts (δ) for ^1H and ^{13}C NMR spectra are reported in parts per million (ppm), relative to the residual solvent peak of the respective NMR solvent ($CDCl_3$ (δ_H = 7.26 and δ_C = 77.16 ppm). Coupling constants (*J*) are given in Hertz (Hz), with the multiplicities being denoted as follows: singlet (s), doublet (d), triplet (t), quartet (q), quintet (qi), multiplet (m), broad (br). NMR spectra for ^{13}C were recorded with decoupling from ^1H. Electrospray ionization–high resolution mass spectrometry (ESI–HRMS) and atmospheric pressure chemical ionization (APCI) for mass spectrometry were recorded on a Waters Micromass Q-Tof micro mass spectrometer.

Elemental analyses were performed by Mikroanalytisches Laboratorium KOLBE (Mülheim an der Ruhr, Germany).

Steady State Absorption Spectroscopy. UV-vis absorption measurements were performed on an Agilent Cary 60 spectrophotometer. Samples were contained in a 10 × 10 mm

fluorescence quartz cuvette with a screw cap and PTFE septum or a 10 mm absorption quartz cuvette.

Stern–Volmer Quenching Studies. Steady-state emission measurements were performed on an Agilent Varian Eclipse fluorimeter using 502 nm as the excitation wavelength with slit widths set to 5 nm spectral resolution using a 10 x 10 mm fluorescence cuvette with a screw cap and PTFE septum. A solution of $[Fe(III)(phtmeimb)_2]PF_6$ (0.16 mM) was prepared in DMF and the emission was recorded at varying quencher concentrations in a range of 10–300 mM following degassing of the solution with argon. UV-vis absorption and two emission spectra were taken for each quencher concentration and emission intensities were corrected for minor differences in absorbance at the excitation wavelength. The shown emission spectra are all averages of two measurements taken for each quencher concentration and were background-substracted. Stern-Volmer plots were obtained by taking the emission values at 642 nm after correction for minor differences in absorbance and all data points result from the average of the two measurements.

Emission lifetimes were determined by Time-Correlated Single Photon Counting (TCSPC) performed with the FS5 (Edinburgh Instruments) fluorimeter. Samples were excited with a 470 nm picosecond pulsed laser diode (EPL Series). Emission decays at 650 nm were recorded with counts of around 133,000. The instrument response function (IRF) was collected with a scattering sample (LUDOX). The kinetic decays were fitted along with the IRF using the in-built Fluoracle® software.

Nanosecond transient absorption measurements. Nanosecond transient absorption measurements were performed with a LP920-S laser flash photolysis spectrometer (Edinburgh Instruments) equipped with an iStar CCD camera (Andor Technology) for transient spectra and a LP920-K PMT detector connected to a TDS 3052 500 MHz 5 GS/s oscilloscope (Tektronix) for single wavelength kinetics. For timescales within 200 μs, the probe light was provided by a pulsed XBO 450 W Xenon Arc Lamp (Osram); for longer timescale measurements, the same probe light was used but set to continuous wave probing rather than pulsed. The samples were excited at 465 nm or 500 nm with 8 ns pulses provided by a frequency tripled Q-switched Nd:YAG laser (EKSPLA NT342B) combined with an optical parametric oscillator (OPO). All measurements were performed at right angle in a 10 × 10 mm quartz cuvette with samples deaerated by purging with argon and an absorbance of around 0.5 at the excitation wavelengths.

Initial Attempts at Utilising Native Amines

In light of the suitable GS and ES redox potentials of our PC $[Fe(III)(phtmeimb)_2]PF_6$, regular amines were intially investigated as precursors to the desired α-aminoalkyl radicals, with triethylamine (TEA) and *N*-methyldiphenylamine (NMDPA) being used primarily.

When using one equivalent or a slight excess of NMDPA or TEA and the Michael acceptor diethyl ethylidenemalonate (**2a**), no conversion to the target product was observed. Only upon incorporating a vast excess of triethylamine (80 equivalents), which was not feasible for *N*-methyldiphenylamine as a result of solubility issues, did we obtain the aminomethylation product, albeit in comparatively low yield of 46 %. The need for such a high concentration of the amine was attributed to the deprotonation of the amine radical cation generated by the single electron oxidation not being as facile as commonly stated in the literature, which has also been stated in selected reports.[4, 5] Consequently, we were able to reduce the concentration of triethylamine to 40 equivalents by introduction of an additional base ($CsCO_3$), resulting in similar yields being obtained. Considering the poor atom economy of this approach, we decided to investigate the use of α-trimethylsilylamines as precursors to the desired α-aminoalkyl radicals.

Table S1: Selected examples for the initial testing of TEA and NMDPA as precursors to α-aminoalkyl radicals, using $[Fe(III)(phtmeimb)_2]PF_6$ as PC (1 mol%, 0.025 mmol, 2.16 mg) and diethyl ethylidenemalonate (**2a**) in 2.5 mL of the indicated solvent. Irradiation at 530 nm for 18 h. ([a] NMR yields determined after work-up and concentration under reduced pressure using mesitylene as internal standard , [b] nr = no reaction, [c] Cs_2CO_3 (1.25 mmol) added as external base).

Entry	Amine	Amount of Amine (mmol)	Solvent	NMR yield (%)[a,b]
**1**	TEA	0.375	MeCN	nr
**2**	TEA	0.3	DCM	nr
**3**	TEA	0.5	DCM	nr
**4**	TEA	21.5	DCM	47
**5**	TEA	2.5	DCM	nr
**6**	TEA	5	DCM	nr
**7**	TEA	10	DCM	nr
**8**[c]	TEA	10	DCM	43
**9**	NMDPA	0.375	NMP	nr
**10**	NMDPA	0.375	DCM	nr
**11**	NMDPA	0.5	DCM	nr
**12**	NMDPA	0.375	DCM	nr
**13**	NMDPA	5	NMP	nr

Synthesis of α-Trimethylsilylamines

(**1a**), (**1b**) and (**1d–g**) were synthesised using a slightly modified literature procedure from Ref 6, (**1c**) was prepared according to Ref 7 and (**1h**) as well as (**1i**) were obtained following a procedure from Ref 8.

N-Phenyl-*N*-((trimethylsilyl)methyl)aniline (1a)

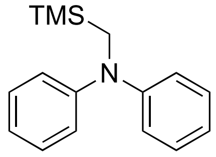

(**1a**)

To a solution of diphenylamine (3.39 g, 20 mmol) in anhydrous THF (0.4 M, 50 mL), *n*-BuLi (2.5 M in hexanes, 8 mL, 20 mmol) was added dropwise at 0 °C. The reaction mixture was stirred for 30 min at 0 °C before removing the ice bath and continuing stirring at room temperature for an additional hour. The reaction was then cooled to 0 °C again and (iodomethyl)trimethylsilane (6.42 g, 4.4 mL, 30 mmol) was added dropwise. Following complete addition, the yellow solution was left to stir at room temperature overnight.

Then water was added and the aqueous phase was extracted thrice with hexane. The organic phase was dried over anhydrous Na_2SO_4 and the solvent was removed *in vacuo*. The product was purified by silica gel column chromatography (14 cm x 4 cm, petroleum ether) giving a colourless oil (**1a**) (4.0 g, 78 %).

R_f = 0.54 (petroleum ether, visualisation by UV). ^{1}H NMR (400 MHz, $CDCl_3$): δ (ppm) = 7.30–7.24 (m, 4H), 7.06–7.00 (m, 4H), 6.95 (tt, J = 7.3, 1.1 Hz, 2H), 3.33 (s, 2H), -0.04 (s, 9H). ^{13}C NMR (101 MHz, $CDCl_3$): δ (ppm) = 149.7, 129.2, 121.2, 121.1, 43.8, -1.2. ESI-HRMS calculated m/z = 256.1522, found m/z = 256.1523 for $[C_{16}H_{21}NSi + H]^+$. Elemental analysis for $C_{16}H_{21}NSi$ (% calc'd, % found): C (75.23, 75.21), H (8.29, 8.28), N (5.48, 5.46).

N-Methyl-*N*-((trimethylsilyl)methyl)aniline (1b)

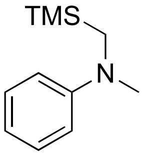

(**1b**)

To a solution of *N*-methylaniline (3.21 g, 3.25 mL, 30 mmol) in anhydrous THF (0.4 M, 70 mL), *n*-BuLi (2.5 M in hexanes, 14.4 mL, 36 mmol) was added dropwise at 0 °C affording a yellow suspension. The reaction mixture was stirred for 30 min at 0 °C before removing the

ice bath and continuing stirring at room temperature for an additional hour. The reaction was then cooled to 0 °C again and (iodomethyl)trimethylsilane (7.71 g, 5.4 mL, 36 mmol) was added dropwise. Following complete addition, the orange solution was left to stir at 50 °C overnight.

Then water was added and the aqueous phase was extracted thrice with hexane and EtOAc respectively. The organic phase was dried over anhydrous Na_2SO_4 and the solvent was removed *in vacuo*. The product was purified by silica gel column chromatography (14 cm x 4 cm, petroleum ether → petroleum ether:EtOAc, 4:1 v/v) giving a dark yellow oil (**1b**) (4.6 g, 79 %).

R_f = 0.6 (petroleum ether:EtOAc, 15:1 v/v, visualisation by UV). ^{1}H NMR (400 MHz, $CDCl_3$): δ (ppm) = 7.23–7.17 (m, 2H), 6.69–6.60 (m, 3H), 2.93 (s, 3H), 2.86 (s, 2H), 0.09 (s, 9H). ^{13}C NMR (101 MHz, $CDCl_3$): δ (ppm) = 150.7, 129.0, 115.3, 112.0, 44.1, 40.3, -1.1. ESI-HRMS calculated m/z = 194.1365, found m/z = 194.1370 for $[C_{11}H_{19}NSi + H]^+$. Elemental analysis for $C_{11}H_{19}NSi$ (% calc'd, % found): C (68.33, 68.19), H (9.90, 9.81), N (7.24, 7.19).

N-Phenyl-*N*-((trimethylsilyl)methyl)aniline (**1c**)

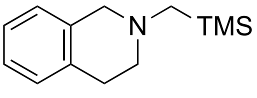

(**1c**)

A solution of 1,2,3,4-tetrahydroisoquinoline (5.32 g, 5 mL, 40 mmol) and (chloromethyl)trimethylsilane (2.46 g, 2.8 mL, 20 mmol) in DMF (20 mL) was heated to 90 °C overnight.

After cooling to room temperature, water (50 mL) was added and the mixture was stirred for an hour. The aqueous phase was extracted thrice with Et_2O, the combined organic phases were washed with water thrice and dried over anhydrous Na_2SO_4. The solvent was removed *in vacuo* and the product was purified by silica gel column chromatography (15 cm x 3 cm, petroleum ether:EtOAc, 4:1 v/v) affording (**1c**) as a light yellow oil. (3.30 g, 75 %)

R_f = 0.6 (petroleum ether:EtOAc, 4:1 v/v, visualisation by UV). ^{1}H NMR (400 MHz, $CDCl_3$): δ (ppm) = 7.31–7.25 (m, 3H), 7.23–7.10 (m, 1H), 3.76 (s, 2H), 3.07 (t, J = 5.9 Hz, 2H), 2.86 (t, J = 5.9 Hz, 2H), 2.26 (s, 2H), 0.30 (s, 9H). ^{13}C NMR (101 MHz, $CDCl_3$): δ (ppm) 135.6, 134.2, 128.7, 126.5, 126.1, 125.6, 60.3, 54.5, 50.7, 29.5, -1.0. ESI-HRMS calculated m/z = 220.1522, found m/z = 220.1519 for $[C_{13}H_{21}NSi + H]^+$. Elemental analysis for $C_{13}H_{21}NSi$ (% calc'd, % found): C (71.17, 70.98), H (9.65, 9.78), N (6.38, 6.26).

4-Bromo-*N*-(4-bromophenyl)-*N*-((trimethylsilyl)methyl)aniline (**1d**)

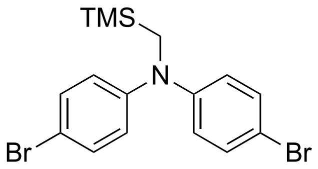

(**1d**)

To a solution of bis(4-bromophenyl)amine (3.27 g, 10 mmol) in anhydrous THF (0.4 M, 25 mL), *n*-BuLi (2.5 M in hexanes, 4 mL, 10 mmol) was added dropwise at 0 °C. The reaction mixture was stirred for 30 min at that temperature before removing the ice bath and continuing stirring at room temperature for an additional hour. The reaction was then cooled to 0 °C again and (iodomethyl)trimethylsilane (3.21 g, 2.22 mL, 15 mmol) was added dropwise. Following complete addition, the green solution was left to stir at room temperature overnight.

Then water was added and the aqueous phase was extracted thrice with hexane. The organic phase was dried over anhydrous Na_2SO_4 and the solvent was removed *in vacuo*. The product was purified by silica gel column chromatography (15 cm x 3 cm, petroleum ether) affording a colourless oil (**1d**) (3.5 g, 85 %).

R_f = 0.48 (petroleum ether, visualisation by UV). ^{1}H NMR (400 MHz, $CDCl_3$): δ (ppm) = 7.37–7.30 (m, 4H), 6.90–6.80 (m, 4H), 3.24 (s, 2H), -0.03 (s, 9H).

^{13}C NMR (101 MHz, $CDCl_3$): δ 148.3, 132.2, 122.8, 113.8, 44.1, -1.1. ESI-HRMS calculated *m/z* = 464.9700, found *m/z* = 464.9698 for $[C_{16}H_{19}NBr_2Si + Na]^+$. Elemental analysis for $C_{16}H_{19}Br_2NSi$ (% calc'd, % found): C (46.51, 46.30), H (4.63, 4.57), N (3.39, 3.36).

4-Methoxy-*N*-(4-methoxyphenyl)-*N*-((trimethylsilyl)methyl)aniline (**1e**)

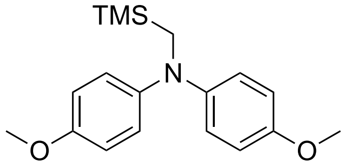

(**1e**)

To a solution of 4,4'-dimethoxydiphenylamine (2.29 g, 10 mmol) in anhydrous THF (0.4 M, 25 mL), *n*-BuLi (2.5 M in hexanes, 4 mL, 10 mmol) was added dropwise at 0 °C. The reaction mixture was stirred for 30 min at that temperature before removing the ice bath and continuing stirring at room temperature for an additional hour. The reaction was then cooled to 0 °C again and (iodomethyl)trimethylsilane (3.21 g, 2.22 mL, 15 mmol) was added dropwise. Following complete addition, the green solution was left to stir at 60 °C overnight.

Then water was added and the aqueous phase was extracted thrice with hexane. The organic phase was dried over anhydrous Na_2SO_4 and the solvent was removed *in vacuo*. The product was purified by silica gel column chromatography (16 cm x 3 cm, petroleum ether:EtOAc, 9:1 v/v) affording a yellow oil (**1e**) (2.35 g, 75 %).

R_f = 0.45 (petroleum ether:EtOAc, 9:1, visualisation by UV). ^{1}H NMR (400 MHz, $CDCl_3$): δ (ppm) = 6.92–6.85 (m, 4H), 6.85–6.78 (m, 4H), 3.78 (s, 6H), 3.16 (s, 2H), -0.07 (s, 9H). ^{13}C NMR (101 MHz, $CDCl_3$): δ 154.3, 144.5, 122.4, 114.6, 55.7, 44.4, -1.3. ESI-HRMS calculated *m/z* = 315.1655, found *m/z* = 315.1648 for [$C_{18}H_{25}NO_2Si$]. Elemental analysis for $C_{18}H_{25}NO_2Si$·0.1 EtOAc (% calc'd, % found): C (68.15, 68.05), H (8.02, 8.03), N (4.32, 4.55).

9-((Trimethylsilyl)methyl)-9*H*-carbazole (**1f**)

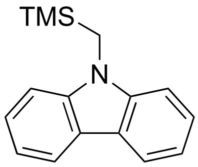

(**1f**)

To a solution of 9*H*-carbazole (1.67 g, 10 mmol) in anhydrous THF (0.4 M, 25 mL), *n*-BuLi (2.5 M in hexanes, 4 mL, 10 mmol) was added dropwise at 0 °C. The reaction mixture was stirred for 30 min at that temperature before removing the ice bath and continuing stirring at room temperature for an additional hour. The reaction was then cooled to 0 °C again and (iodomethyl)trimethylsilane (3.21 g, 2.22 mL, 15 mmol) was added dropwise. Following complete addition, the beige suspension was left to stir at 60 °C overnight.

Then water was added and the aqueous phase was extracted thrice with hexane. The organic phase was dried over anhydrous Na_2SO_4 and the solvent was removed *in vacuo*. The product was purified by silica gel column chromatography (16 cm x 3 cm, petroleum ether) affording a colourless oil that turned into a waxy solid upon standing (**1f**) (1.95 g, 77 %).

R_f = 0.2 (petroleum ether, visualisation by UV). ^{1}H NMR (400 MHz, $CDCl_3$): δ (ppm) = 8.11 (ddd, *J* = 7.8, 1.2, 0.7 Hz, 2H), 7.46 (ddd, *J* = 8.3, 7.1, 1.2 Hz, 2H), 7.34 (dt, *J* = 8.3, 0.9 Hz, 2H), 7.21 (ddd, *J* = 7.9, 7.1, 1.0 Hz, 2H), 3.87 (s, 2H), 0.08 (s, 9H). ^{13}C NMR (101 MHz, $CDCl_3$): δ 140.8, 125.5, 122.6, 120.3, 118.4, 109.0, 34.4, -1.2. ESI-HRMS calculated *m/z* = 254.1365, found *m/z* = 254.1365 for [$C_{16}H_{19}NSi$ + H]$^+$. Elemental analysis for $C_{16}H_{19}NSi$ (% calc'd, % found): C (75.83, 75.62), H (7.56, 7.53), N (5.53, 5.51).

9-((Trimethylsilyl)methyl)-9*H*-carbazole (**1g**)

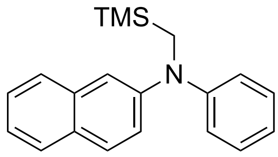

(**1g**)

To a solution of *N*-phenylnaphthalen-2-amine (2.19 g, 10 mmol) in anhydrous THF (0.4 M, 25 mL), *n*-BuLi (2.5 M in hexanes, 4 mL, 10 mmol) was added dropwise at 0 °C. The reaction mixture was stirred for 30 min at that temperature before removing the ice bath and continuing stirring at room temperature for an additional hour. The reaction was then cooled to 0 °C again and (iodomethyl)trimethylsilane (3.21 g, 2.22 mL, 15 mmol) was added dropwise. Following complete addition, the suspension was left to stir at 60 °C overnight.

Then water was added and the aqueous phase was extracted thrice with hexane. The organic phase was dried over anhydrous Na_2SO_4 and the solvent was removed *in vacuo*. The product was purified by silica gel column chromatography (15 cm x 3 cm, *n*-heptane) affording a beige waxy solid (**1g**) (1.83 g, 60 %).

R_f = 0.27 (*n*-heptane, visualisation by UV). ^{1}H NMR (400 MHz, $CDCl_3$): δ (ppm) = 7.70–7.58 (m, 3H), 7.37 (ddd, *J* = 8.2, 6.8, 1.3 Hz, 1H), 7.29–7.23 (m, 4H), 7.15 (dd, *J* = 8.9, 2.4 Hz, 1H), 7.08–7.03 (m, 2H), 6.99–6.92 (m, 1H), 3.41 (s, 2H), -0.02 (s, 9H). ^{13}C NMR (101 MHz, $CDCl_3$): δ (ppm) = 149.7, 147.1, 134.8, 129.3, 129.0, 128.6, 127.6, 126.7, 126.3, 123.6, 122.3, 121.9, 115.0, 44.3, -1.0. ESI-HRMS calculated *m/z* = 306.1678, found *m/z* = 306.1681 for [$C_{20}H_{23}NSi + H^+$]. Elemental analysis for $C_{20}H_{23}NSi \cdot 0.5\ H_2O$ (% calc'd, % found): C (78.40, 78.06), H (7.60, 7.45), N (4.57, 4.51).

N-benzyl-1-(trimethylsilyl)methanamine (**1h**)

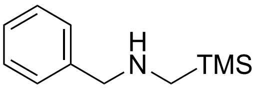

(**1h**)

A solution of benzylamine (0.54 g, 0.55 mL, 5 mmol), (iodomethyl)trimethylsilane (1.07 g, 0.74 mL, 5 mmol) and K_2CO_3 (1.38 g, 10 mmol) in anhydrous MeCN (100 mL) was heated to 80 °C overnight.

After cooling to room temperature, water was added to the reaction mixture until all solids had dissolved and the mixture was extracted thrice with DCM. The organic phase was dried over anhydrous $MgSO_4$ and concentrated *in vacuo*. The product was purified by silica gel column

chromatography (14 cm x 3 cm, petroleum ether:EtOAc 9:1 → 7:1 v/v) affording a colourless oil (**1h**) (673 mg, 70 %).

R_f = 0.27 (petroleum ether:EtOAc 9:1 v/v, visualisation by UV). ^{1}H NMR (400 MHz, $CDCl_3$): δ (ppm) = 7.30–7.23 (m, 4H), 7.22–7.16 (m, 1H), 3.75 (s, 2H), 1.24 (s, 1H), 0.00 (s, 9H). ^{13}C NMR (101 MHz, $CDCl_3$): δ 140.6, 128.3, 128.2, 126.8, 58.1, 39.5, -2.5. ESI-HRMS calculated *m/z* = 194.1365, found *m/z* = 194.1369 for $[C_{11}H_{19}NSi + H]^+$. Elemental analysis for $C_{11}H_{19}NSi$ (% calc'd, % found): C (68.33, 68.16), H (9.90, 9.92), N (7.24, 7.20).

Tert-butylbenzyl((trimethylsilyl)methyl)carbamate (**1i**)

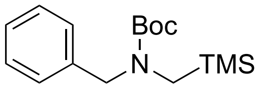

(**1i**)

To a solution of *N*-benzyl-1-(trimethylsilyl)methanamine (**1h**) (963 mg, 4.98 mmol) in anhydrous MeCN (10 mL), di-*tert*-butyl decarbonate (1.07 g, 1.12 mL, 4.88 mmol) was added dropwise at 0 °C and the reaction mixture was stirred at room temperature for 2 h.

The reaction was then quenched by addition of a saturated aqueous solution of NH_4Cl (20 mL) and the product was extracted thrice with DCM. The organic phase was dried over anhydrous $MgSO_4$ and the solvent was removed *in vacuo*. The product was purified by silica gel column chromatography (18 x 3 cm, petroleum ether:EtOAc 9:1 v/v) affording a colourless oil (**1i**) (964 mg, 66 %).

R_f = 0.55 (petroleum ether:EtOAc, 9:1 v/v, visualisation by UV). ^{1}H NMR (400 MHz, $CDCl_3$) (rotamers): δ 7.41–7.22 (m, 5H), 4.49 & 4.45 (s, 2H), 2.76 & 2.72 (s, 2H), 1.56 & 1.49 (s, 2H), 0.09 (s, 9H). ^{13}C NMR (101 MHz, $CDCl_3$) (rotamers): δ 156.2 & 155.5, 138.5 & 138.2, 128.5, 127.8, 127.3, 127.2, 79.5 & 79.3, 52.8 & 51.7, 37.9 & 37.3, 28.6 & 28.5, 27.47, -1.38. ESI-HRMS calculated *m/z* = 293.1811, found *m/z* = 293.1821 for $[C_{16}H_{27}NO_2Si]$. Elemental analysis for $C_{16}H_{27}NO_2Si$ (% calc'd, % found): C (65.48, 65.10), H (9.27, 9.31), N (4.77, 4.73).

Visible light-mediated addition of α-amino alkyl radicals to electron-poor double bonds *via* reductive quenching of $[Fe(phtmeimb)_2]PF_6$

General Procedure for the Photocatalytic Aminomethylation of Electron-Deficient Systems

Photochemical reactions were conducted in a TAK120 AC photoreactor purchased from HK Testsysteme GmbH, unless otherwise specified. A solution of the photocatalyst (PC), electron-deficient alkene substrate (0.25 mmol) and TMS-methylamine in the indicated solvent (2.5 mL) was charged to a 6 mL clear glass crimp top vial equipped with a magnetic stir bar. The reaction vessel was sealed with an aluminium cap with septum and degassed with argon for 5 min. The reaction mixture was then irradiated in the photoreactor at 530 nm (3.15 W per slot) for 15 h. Following irradiation, the reaction mixture was added to aq. $NaHCO_3$ (sat.) and the product was extracted thrice with DCM. The combined organic phases were concentrated under reduced pressure and the product was obtained by isolation via silica gel column chromatography affording elemental analysis pure products.

Optimisations of the reaction conditions

Optimisations were conducted following the General Procedure described above using diethyl ethylidenemalonate (**2a**) and *N*-phenyl-*N*-((trimethylsilyl)methyl)aniline (**1a**) as starting materials. All equivalent amounts and catalyst loadings were given in relation to (**2a**) and performed in the indicated solvent unless otherwise stated. NMR yields were determined following work-up and concentration under reduced pressure using mesitylene as internal standard.

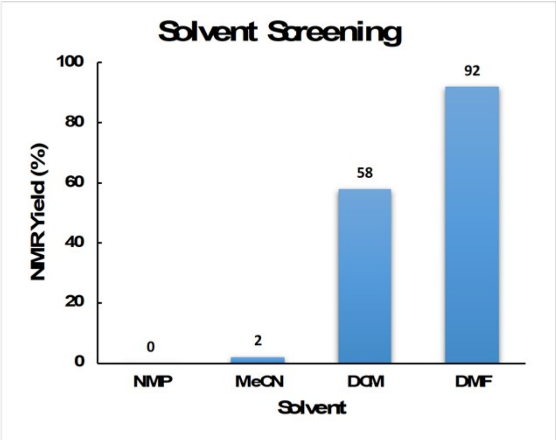

Figure S1: Screening of different solvents using 1 mol% $[Fe(III)(phtmeimb)_2]PF_6$ and 1 equivalent of TMS-methylamine.

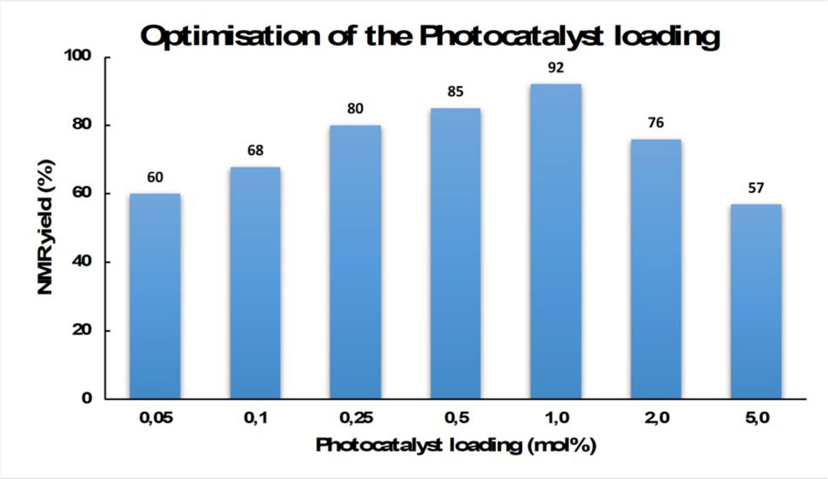

Figure S2: Optimisation of the photocatalyst loading using 1 equivalent of TMS-methylamine in DMF.

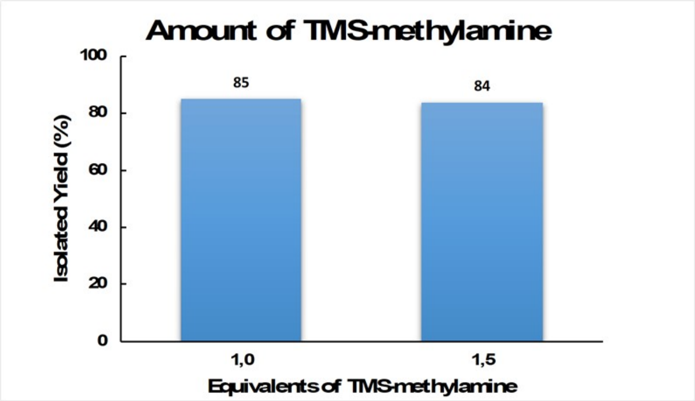

Figure S3: Variation of the amount of TMS-methylamine using 1 mol% $[Fe(III)(phtmeimb)_2]PF_6$ in DMF.

Control Experiments

Table S2: Control experiments with different photocatalysts (1 mol%) using 1 equivalent of TMS-methylamine in DMF, reaction time = 19 h. ([a] NMR yields determined after work-up and concentration under reduced pressure using mesitylene as internal standard , [b] no reaction, [c] no photocatalyst, [d] no photocatalyst and no light).

Entry	Photocatalyst	Irradiation wavelength (nm)	NMR yield (%)[a]
**1**	$[Fe(btz)_3](PF_6)_3$	530	12
**2**	$[Fe(bpy)_3](PF_6)_2$	530	nr[b]
**3**	$FeBr_2$	530	nr
**4**	$[Ru(bpy)_3]Cl_2 \cdot 6\ H_2O$	450	64
**6**[c]	-	530	nr
**7**[d]	-	-	nr

Optimised Procedure for the Photocatalytic Aminomethylation of Electron-Deficient Systems

Through optimisation reactions the following protocol for the photocatalytic aminomethylation of electron-deficient systems was established:

A solution of $[Fe(III)(phtmeimb)_2]PF_6$ (PC) (1 mol%, 2.5 μmol, 2.16 mg), electron-deficient alkene substrate (0.25 mmol) and TMS-methylamine (0.25 mmol, 1 equiv.) in DMF (2.5 mL) was charged to a 6 mL clear glass crimp top vial equipped with a magnetic stir bar. The reaction vessel was sealed with an aluminium cap with septum and degassed with argon for 5 min. The reaction solution was then irradiated in the TAK120 AC photoreactor at 530 nm (3.15 W per slot) for 15 h. Following irradiation, the reaction mixture was added to aq. $NaHCO_3$ (sat.) and the product was extracted thrice with DCM. The combined organic phases were concentrated under reduced pressure and the product was obtained by isolation via silica gel column chromatography affording elemental analysis pure products.

Scope of different TMS-methylsilanes using the Optimised Procedure

Diethyl 2-(1-(diphenylamino)propan-2-yl)malonate (**3a**)

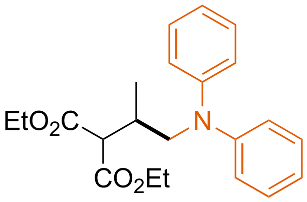

(**3a**)

The photocatalytic reaction was performed according to the Optimised Procedure and purification by silica gel column chromatography (15 cm x 3 cm, petroleum ether/EA = 60:1–8:2) afforded (**3a**) as a dark-yellow oil (157 mg, 85 %).

R_f = 0.26 (petroleum ether:EtOAc, 15:1 v/v, visualization by UV). ^1H NMR (400 MHz, $CDCl_3$): δ (ppm) = 7.31–7.19 (m, 4H), 7.07–7.01 (m, 4H), 6.96 (tt, J = 7.3, 1.1 Hz, 2H), 4.26–4.10 (m, 4H), 3.92 (dd, J = 14.6, 6.3 Hz, 1H), 3.62 (dd, J = 14.6, 8.7 Hz, 1H), 3.45 (d, J = 6.3 Hz, 1H), 2.72 (dh, J = 8.8, 6.7 Hz, 1H), 1.26 (td, J = 7.1, 2.9 Hz, 6H), 1.11 (d, J = 6.9 Hz, 3H). ^{13}C NMR (101 MHz, $CDCl_3$): δ (ppm) = 169.0, 168.7, 148.8, 129.4, 121.7, 121.6, 61.5, 56.3, 54.9, 32.5, 15.4, 14.2. ESI-HRMS calculated m/z = 370.2018, found m/z = 370.2018 for $[C_{22}H_{27}NO_4 + H]^+$. Elemental analysis for $C_{22}H_{27}NO_4$ (% calc'd, % found): C (71.52, 71.24), H (7.37, 3.77), N (3.79, 3.77).

Diethyl 2-(1-(methyl(phenyl)amino)propan-2-yl)malonate (**3b**)

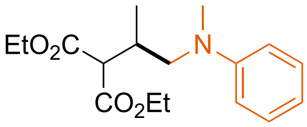

(**3b**)

The photocatalytic reaction was performed according to the Optimised Procedure and purification by silica gel column chromatography (15 cm x 3 cm, petroleum ether:EtOAc, 15:1–8:2 v/v) afforded (**3b**) as a brown oil (41.6 mg, 54 %).

R_f = 0.20 (*n*-heptane:EtOAc, 15:1 v/v, visualization by UV). ^{1}H NMR (400 MHz, $CDCl_3$): δ (ppm) = 7.25 – 7.13 (m, 2H), 6.78 – 6.68 (m, 3H), 4.25 – 4.07 (m, 4H), 3.44 (dd, *J* = 14.5, 7.1 Hz, 1H), 3.35 (d, *J* = 6.8 Hz, 1H), 3.16 (dd, *J* = 14.6, 8.1 Hz, 1H), 2.93 (s, 3H), 2.75 (p, *J* = 7.1 Hz, 1H), 1.24 (dt, *J* = 11.8, 7.1 Hz, 6H), 1.04 (d, *J* = 6.9 Hz, 3H). ^{13}C NMR (101 MHz, $CDCl_3$): δ (ppm) = 168.9, 168.7, 149.8, 129.2, 116.6, 112.5, 61.4, 57.3, 55.2, 39.5, 32.8, 15.5, 14.2, 14.1. ESI-HRMS calculated *m/z* = 308.1862, found *m/z* = 308.1864 for $[C_{22}H_{25}NO_4 + H]^+$. Elemental analysis for $C_{17}H_{25}NO_4$ (% calc'd, % found): C (66.43, 66.19), H (8.20, 8.24), N (4.56, 4.51).

Diethyl 2-(1-(bis(4-bromophenyl)amino)propan-2-yl)malonate (**3d**)

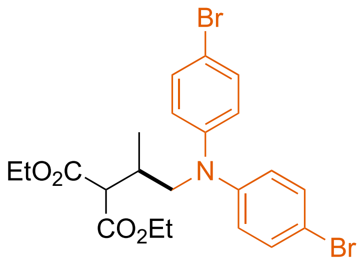

(**3d**)

The photocatalytic reaction was performed according to the Optimised Procedure and purification by silica gel column chromatography (15 cm x 3 cm, petroleum ether:EtOAc, 30:1–7:1 v/v) afforded (**3d**) as an orange-red oil (207 mg, 79 %).

R_f = 0.15 (petroleum ether:EtOAc, 20:1 v/v, visualisation by UV). ^{1}H NMR (400 MHz, $CDCl_3$): δ (ppm) = 7.41–7.29 (m, 4H), 6.96–6.84 (m, 4H), 4.18 (qt, *J* = 7.1, 4.1 Hz, 4H), 3.86 (dd, *J* = 14.6, 6.2 Hz, 1H), 3.53 (dd, *J* = 14.7, 9.0 Hz, 1H), 3.38 (d, *J* = 6.3 Hz, 1H), 2.65 (dh, *J* = 9.0, 6.8 Hz, 1H), 1.25 (td, *J* = 7.1, 1.3 Hz, 6H), 1.07 (d, *J* = 6.8 Hz, 3H). ^{13}C NMR (101 MHz, $CDCl_3$): δ (ppm) = 168.7, 168.5, 147.4, 132.4, 123.2, 114.6, 61.6, 61.6, 56.2, 54.8, 32.1, 15.5, 14.2. ESI-HRMS calculated *m/z* = 548.0048, found *m/z* = 548.0034 for $[C_{22}H_{25}Br_2NO_4 + Na]^+$. Elemental analysis for $C_{22}H_{25}Br_2NO_4$ (% calc'd, % found): C (50.12, 50.12), H (4.78, 4.76), (2.66, 2.65).

Diethyl 2-(1-(bis(4-methoxyphenyl)amino)propan-2-yl)malonate (**3e**)

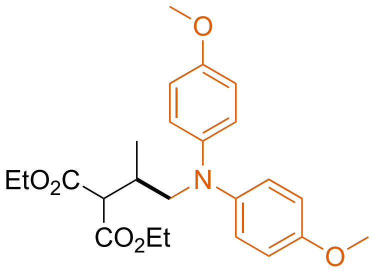

(**3e**)

The photocatalytic reaction was performed according to the Optimised Procedure and purification by silica gel column chromatography (15 cm x 3 cm, petroleum ether:EtOAc, 15:1–3:7 v/v) afforded (**3e**) as a yellow oil (121 mg, 56 %).

R_f = 0.16 (petroleum ether:EtOAc, 15:1 v/v, visualization by UV). ^{1}H NMR (400 MHz, $CDCl_3$): δ (ppm) = 6.95–6.87 (m, 4H), 6.85–6.76 (m, 4H), 4.17 (qd, *J* = 7.2, 1.9 Hz, 4H), 3.77 (s, 6H), 3.50–3.40 (m, 2H), 2.63 (dt, *J* = 14.0, 6.9 Hz, 1H), 1.24 (td, *J* = 7.2, 0.9 Hz, 6H), 1.09 (d, *J* = 6.9 Hz, 3H). ^{13}C NMR (101 MHz, $CDCl_3$): δ (ppm) = 169.1, 168.7, 154.6, 143.2, 122.8, 114.7, 61.4, 61.31, 57.2, 55.7, 54.8, 32.4, 15.4, 14.2. ESI-HRMS calculated *m/z* = 430.2230, found *m/z* = 430.2235 for $[C_{24}H_{31}NO_6 + H]^+$. Elemental analysis for $C_{24}H_{31}NO_6{\cdot}0.1\ H_2O$ (% calc'd, % found): C (66.83, 66.59), H (7.29, 7.37), (3.25, 3.21).

Diethyl 2-(1-(9*H*-carbazol-9-yl)propan-2-yl)malonate (**3f**)

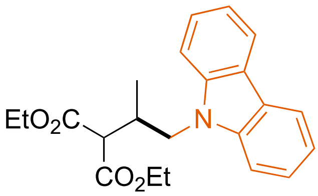

(**3f**)

The photocatalytic reaction was performed according to the Optimised Procedure and purification by silica gel column chromatography (15 cm x 3 cm, petroleum ether:EtOAc, 30:1–7:2 v/v) afforded (**3f**) as a colourless solid (141 mg, 77 %).

R_f = 0.39 (petroleum ether:EtOAc, 15:1 v/v, visualisation by UV). ^{1}H NMR (400 MHz, $CDCl_3$): δ (ppm) = 8.37 (dt, *J* = 7.8, 1.0 Hz, 2H), 7.82 (dt, *J* = 8.3, 1.0 Hz, 2H), 7.75 (ddd, *J* = 8.2, 7.1, 1.2 Hz, 2H), 7.52 (ddd, *J* = 8.0, 6.1, 1.0 Hz, 2H), 4.79 (dd, *J* = 14.6, 5.2 Hz, 1H), 4.60–4.44 (m, 5H), 3.70 (dd, *J* = 6.9, 0.8 Hz, 1H), 3.41–3.26 (m, 1H), 1.58 (td, *J* = 7.1, 1.0 Hz, 6H), 1.27 (d, *J* = 6.9 Hz, 3H). ^{13}C NMR (101 MHz, $CDCl_3$): δ (ppm) = 168.6, 168.5, 140.9, 125.9, 123.0, 120.4, 119.2, 109.3, 61.7, 61.7, 55.3, 46.8, 33.5, 15.9, 14.3, 14.2. ESI-HRMS calculated

m/z = 390.1681, found m/z = 390.1678 for $[C_{22}H_{25}NO_4 + Na]^+$. Elemental analysis for $C_{22}H_{25}NO_4$ (% calc'd, % found): C (71.91, 71.86), H (6.86, 6.86), N (3.81, 3.79).

Diethyl 2-(1-(naphthalen-2-yl(phenyl)amino)propan-2-yl)malonate (**3g**)

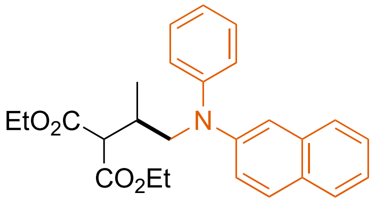

(**3g**)

The photocatalytic reaction was performed according to the Optimised Procedure and purification by silica gel column chromatography (15 cm x 3 cm, n-heptane:EtOAc, 20:1–8:2 v/v) afforded (**3g**) as a yellow oil (131 mg, 62 %).

R_f = 0.22 (*n*-heptane:EtOAc, 15:1 v/v, visualization by UV). ^{1}H NMR (400 MHz, $CDCl_3$): δ (ppm) = 7.72 (dd, J = 17.2, 8.3 Hz, 3H), 7.47–7.37 (m, 2H), 7.36–7.24 (m, 4H), 7.22–7.15 (m, 1H), 7.09 (d, J = 7.6 Hz, 2H), 7.00 (td, J = 7.3, 1.2 Hz, 1H), 4.26–4.10 (m, 4H), 4.04 (dd, J = 14.6, 6.3 Hz, 1H), 3.73 (dd, J = 14.6, 8.7 Hz, 1H), 3.49 (dd, J = 6.3, 1.3 Hz, 1H), 2.80 (dq, J = 9.0, 6.5 Hz, 1H), 1.25 (qd, J = 7.2, 1.3 Hz, 6H), 1.15 (dd, J = 6.8, 1.3 Hz, 3H). ^{13}C NMR (101 MHz, $CDCl_3$): δ (ppm) = 169.0, 168.7, 148.9, 146.0, 134.7, 129.4, 128.9, 127.6, 127.1, 126.4, 124.1, 123.0, 122.1, 121.9, 116.8, 61.5 (d, J = 6.4 Hz), 56.4, 54.9, 32.3, 15.5, 14.2 (d, J = 2.4 Hz). ESI-HRMS calculated m/z = 420.2175, found m/z = 420.2184 for $[C_{26}H_{29}NO_4 + H]^+$. Elemental analysis for $C_{26}H_{29}NO_4$ (% calc'd, % found): C (74.44, 74.21), H (6.97, 6.99), N (3.34, 3.33).

Scope of different Michael Acceptors using the Optimised Procedure

2-(2-(Diphenylamino)-1-phenylethyl)malononitrile

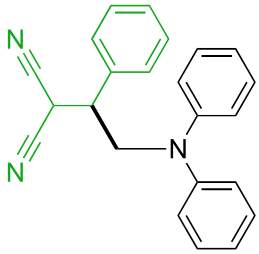

(**3j**)

The photocatalytic reaction was performed according to the Optimised Procedure and purification by silica gel column chromatography (15 cm x 3 cm, petroleum ether:EtOAc, 18:1–8:2 v/v) afforded (**3j**) as a yellow waxy solid (126 mg, 75 %).

R_f = 0.29 (petroleum ether:EtOAc, 15:1 v/v, visualization by UV). ^{1}H NMR (400 MHz, $CDCl_3$): δ (ppm) = 7.47–7.40 (m, 3H), 7.40–7.35 (m, 2H), 7.34–7.28 (m, 4H), 7.07 (td, *J* = 7.4, 1.1 Hz, 2H), 7.01–6.93 (m, 4H), 4.40 (d, *J* = 4.5 Hz, 1H), 4.30 (dd, *J* = 15.0, 10.1 Hz, 1H), 4.15 (dd, *J* = 14.9, 5.1 Hz, 1H), 3.62 (dt, *J* = 9.8, 4.8 Hz, 1H).

^{13}C NMR (101 MHz, $CDCl_3$): δ (ppm) = 147.9, 134.7, 129.9, 129.54, 129.48, 128.4, 123.3, 122.0, 112.2, 111.7, 54.5, 44.9, 27.2. ESI-HRMS calculated *m/z* = 337.1579, found *m/z* = 337.1584 for $[C_{23}H_{19}N_3 + H]^+$. Elemental analysis for $C_{23}H_{19}N_3$ (% calc'd, % found): C (81.87, 81.52), H (5.68, 5.59), N (12.45, 12.39).

3-((Diphenylamino)methyl)cyclohexan-1-one (**3k**)

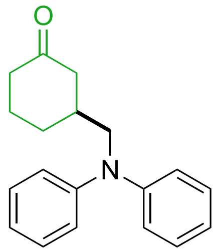

(**3k**)

The photocatalytic reaction was performed according to the Optimised Procedure and purification by silica gel column chromatography (25 cm x 3 cm, *n*-heptane:EtOAc, 10:1 v/v) afforded (**3k**) as a pale-yellow oil (33 mg, 47 %).

R_f = 0.14 (*n*-heptane/EtOAc, 10:1, visualisation by UV). ^{1}H NMR (400 MHz, $CDCl_3$) δ 7.29–7.19 (m, 4H), 7.08–6.79 (m, 6H), 3.65 (qd, *J* = 14.7, 7.0 Hz, 2H), 2.58–2.47 (m, 1H), 2.41–2.30 (m, 1H), 2.30–2.18 (m, 1H), 2.14–1.95 (m, 3H), 1.64–1.56 (m, 1H), 1.44–1.32 (m, 1H). ^{13}C-NMR (101 MHz, CDCl3) δ (ppm) = 211.0, 148.6, 129.4, 121.7, 121.3, 58.3, 46.1, 41.6, 38.2, 29.6, 25.2. ESI-HRMS calculated *m/z* = 280.1701, found *m/z* = 280.1691 for $[C_{19}H_{21}NO + H]^+$. Elemental analysis for $C_{19}H_{21}NO$ (% calc'd, % found): C (81.68, 81.61), H (7.58, 7,60), N (5.01, 4,99).

3-((Diphenylamino)methyl)cyclopentan-1-one (**3l**)

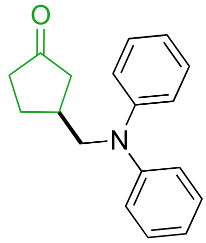

(**3l**)

The photocatalytic reaction was performed according to the Optimised Procedure and purification by silica gel column chromatography (*n*-heptane:EtOAc, 10:1 v/v) afforded a colourless oil (37.5 mg, 57 %).

R_f = 0.33 (*n*-heptane/EtOAc, 5:1 v/v, visualisation by UV). ^{1}H-NMR (400 MHz, $CDCl_3$): δ (ppm) = 7.34–7.24 (m, 4H), 7.08–6.92 (m, 6H), 3.88–3.73 (m, 2H), 2.78–2.62 (m, 1H), 2.45–2.26 (m, 2H), 2.26–2.09 (m, 2H), 1.93 (ddd, *J* = 18.4, 9.9, 1.4 Hz, 1H), 1.73–1.57 (m, 1H). ^{13}C-NMR (101 MHz, CDCl3) δ (ppm) = 218.7, 148.5, 129.6, 121.9, 121.3, 57.1, 43.7, 38.2, 36.4, 27.7. ESI-HRMS calculated *m/z* = 266.1545, found *m/z* = 266.1525 for $[C_{18}H_{19}NO + H]^+$. Elemental analysis for $C_{18}H_{19}NO$ (% calc'd, % found): C (81.47, 81.44), H (7.22, 7.20), N (5.28, 5.28).

5-(Diphenylamino)pentan-2-one (**3m**)

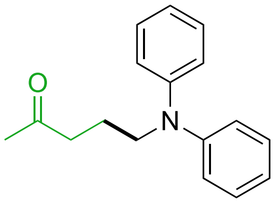

(3m)

The photocatalytic reaction was performed according to the Optimised Procedure and purification by silica gel column chromatography (15 cm x 3 cm, petroleum ether:EtOAc, 30:1→15:1 v/v) afforded (**3m**) as a brown oil (35 mg, 55 %).

R_f = 0.26 (petroleum ether:EtOAc, 20:1 v/v, visualisation by UV). ^{1}H-NMR (400 MHz, $CDCl_3$): δ (ppm) = 7.28–7.24 (m, 4H), 7.05–6.98 (m, 4H), 6.97–6.92 (m, 2H), 3.76–3.68 (m, 2H), 2.49 (t, *J* = 7.0 Hz, 2H), 2.11 (s, 3H), 1.93 (p, *J* = 7.1 Hz, 2H). ^{13}C-NMR (101 MHz, CDCl3) δ (ppm) = 208.4, 148.1, 129.4, 121.5, 121.1, 51.4, 40.8, 30.1, 21.8. ESI-HRMS calculated *m/z* = 254.1545, found *m/z* = 254.1541 for $[C_{17}H_{19}NO + H]^+$. Elemental analysis for $C_{17}H_{19}NO{\cdot}0.15\ H_2O$ (% calc'd, % found): C (79.75, 79.35), H (7.60, 7.51), N (5.47, 5.45).

Diethyl 2-(2-(diphenylamino)-1-ethoxyethyl)malonate (**3n**)

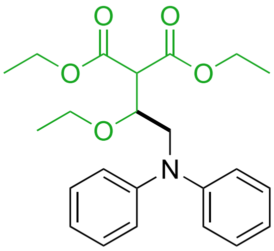

(3n)

The photocatalytic reaction was performed according to the Optimised Procedure and purification by silica gel column chromatography (petroleum ether:diethyl ether, 40:1→10:1 v/v) afforded (**3n**) as a colourless oil (27 mg, 27 %) after two rounds of purification.

R_f = 0.39 (petroleum ether:diethyl ether, 5:1 v/v, visualisation by UV). ^{1}H-NMR (400 MHz, $CDCl_3$): δ (ppm) = 7.35 – 7.23 (m, 4H), 7.20 – 7.12 (m, 4H), 6.96 (tt, J = 7.3, 1.1 Hz, 2H), 4.35 – 4.08 (m, 6H), 3.84 (dd, J = 15.2, 9.2 Hz, 1H), 3.69 – 3.56 (m, 2H), 3.51 (dq, J = 9.0, 7.0 Hz, 1H), 1.29 (td, J = 7.2, 5.9 Hz, 6H), 1.04 (t, J = 7.0 Hz, 3H). ^{13}C-NMR (101 MHz, CDC13) δ (ppm) = 167.5, 148.3, 129.3, 121.5, 121.3, 75.6, 68.0, 61.7 (d, J = 7.1 Hz), 55.9 (d, J = 8.8 Hz), 15.7, 14.2 (d, J = 6.5 Hz). ESI-HRMS calculated m/z = 400.2124, found m/z = 400.2131 for $[C_{23}H_{29}NO_5 + H]^+$. Elemental analysis for $C_{23}H_{29}NO_5$ (% calc'd, % found): C (69.15, 68.85), H (7.32, 7.39), N (3.51, 3.47).

(5*R*)-3-((diphenylamino)methyl)-2-methyl-5-(prop-1-en-2-yl)cyclohexan-1-one (3o)

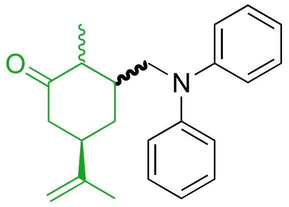

(**3o**)

The photocatalytic reaction was performed according to the Optimised Procedure and purification by silica gel column chromatography (*n*-heptane:EtOAc, 50:1→20:1 v/v) afforded (**3o**) as a mixture of diastereomers in the form of a colourless oil (26.5 mg, 32 %, d.r.: 0.6 (Diastereomer **1**):1 (Diastereomer **2**) :1.6 (Diastereomer **3**)).

R_f = 0.33 (*n*-heptane:EtOAc, 20:1 v/v, visualisation by UV).

Characterisations of the purified mixture:

^{1}H-NMR (400 MHz, $CDCl_3$): δ (ppm) = 7.32–7.2 (m, 4H), 7.03–6.91 (m, 6H), 4.74 (s, 1H, diastereomers **1** and **2**), 4.69 (s, 1H, diastereomer **3**), 4.67 (s, 1 H, diastereomer **1**), 4.61 (s, 1H, diastereomer **2**), 4.54 (s, 1H, diastereomer **3**), 4.14 (dd, J = 3.7 Hz, 14.7 Hz, 1H, diastereomer **1**), 3.90–3.77 (m, 1H, diastereomer **2** & **3**), 3.67–3.43 (m, 1H, diastereomer **1** & **3**), 2.81–2.71 (m, 2H, diastereomer **3**), 2.63–2.53 (m, 2H, diastereomer **2**), 2.51–2.06 (m, 4H), 2.02–1.89 (m, 1H, diastereomer **1**), 1.81–1.65 (m, 1H), 1.81 (m, 1H, diastereomer **2** & **3**), 1.69 (s, diastereomer **1**), 1.56 (s, 3H, diastereomer **2**), 1.54 (s, 3H, diastereomer **3**), 1.22–1.14 (m, 3H). ^{13}C-NMR (101 MHz, CDC13) δ (ppm) = 213.4, 212.7, 211.5, 148.7, 148.7, 147.5, 147.1, 146.7, 129.5, 129.4, 121.8, 121.7, 121.4, 121.3, 111.4, 110.4, 109.8, 57.2, 56.2, 50.8, 48.3, 47.9, 47.5, 46.7, 46.2, 44.6, 43.8, 43.5, 41.5, 40.7, 40.2, 39.4, 35.8, 31.9, 30.0, 29.8, 21.2, 20.7, 20.5, 14.7,

11.7, 11.6. ESI-HRMS calculated m/z = 334.2171, found m/z = 334.2181 for $[C_{23}H_{27}NO + H]^+$. Elemental analysis for $C_{23}H_{27}NO$ (% calc'd, % found): C (82.84, 82.62), H (8.16, 8.14), N (4.20, 4.18).

Mechanistic Investigations

The effect of radical scavengers

Addition of TEMPO (2,2,6,6-tetramethylpiperidin-1-yl)oxyl) to the reaction performed using the Optimised Procedure supported the presence of a radical mechanism due to the adduct between the α-aminoalkyl radical and TEMPO being observed in the reaction and the expected reaction product (**3a**) only being afforded in trace amounts.

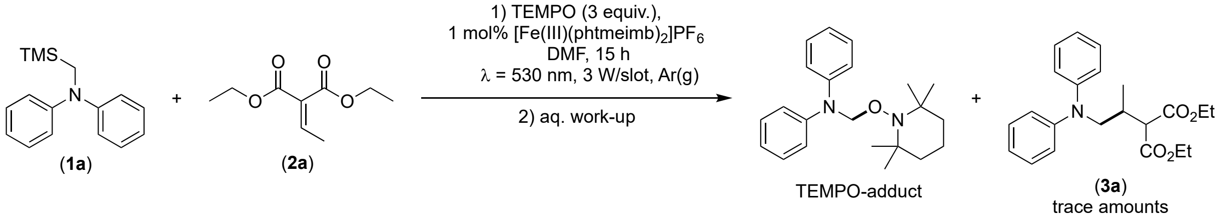

Scheme S.1: Investigation of the formation of α-aminoalkyl radicals in the visible light-mediated aminomethylation of (**2a**) using TEMPO (2,2,6,6-tetramethylpiperidin-1-yl)oxyl) as radical scavenger.

Photodegradation Studies

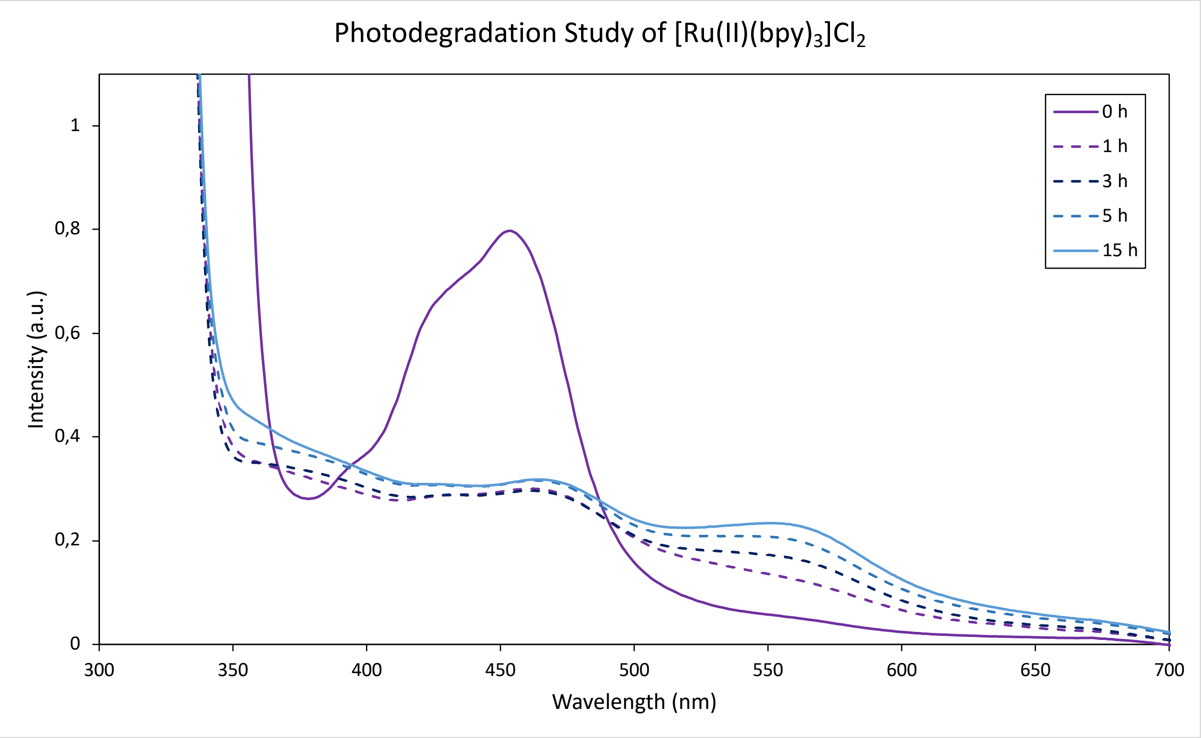

Figure S4: Study of the photodegradation of $[Ru(II)(bpy)_3]Cl_2$ during the model reaction of (**1a**) and (**2a**) using the General Procedure under irradiation at 450 nm.

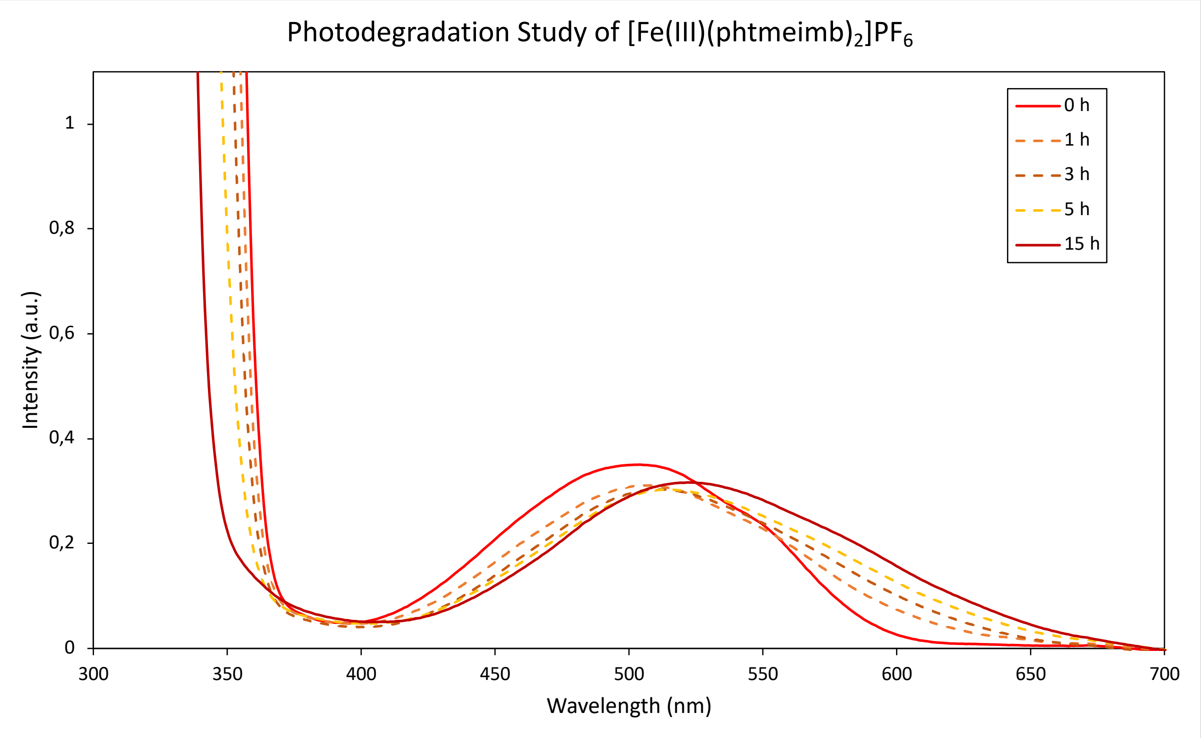

Figure S5: Study of the photodegradation of [Fe(III)(phtmeimb)$_2$]PF$_6$ during the model reaction of (**1a**) and (**2a**) using the General Procedure under irradiation at 530 nm.

Stern–Volmer quenching studies

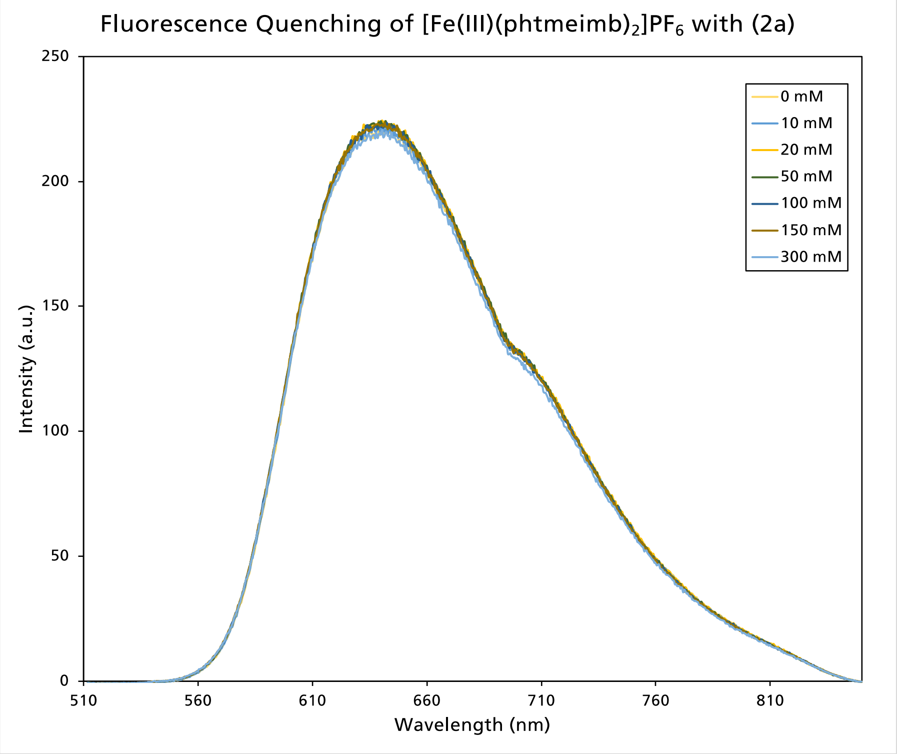

Figure S6: Emission quenching of [Fe(III)(phtmeimb)$_2$]PF$_6$ by substrate (**2a**) in DMF.

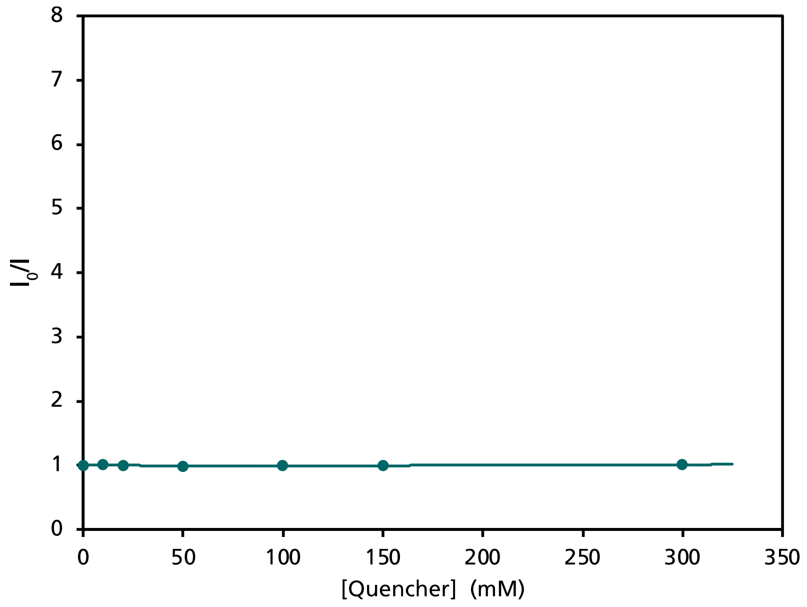

Figure S7: Stern–Volmer plot derived from steady-state emission quenching of [Fe(III)(phtmeimb)$_2$]PF$_6$ (0.16 mM) by substrate (**2a**) in DMF.

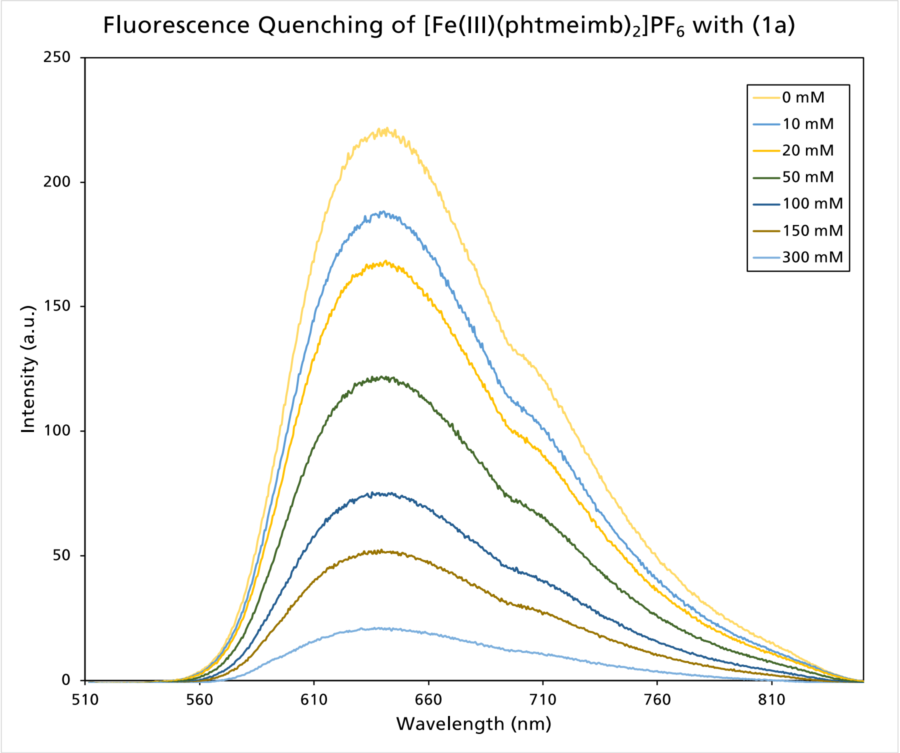

Figure S8: Emission quenching of [Fe(III)(phtmeimb)$_2$]PF$_6$ by substrate (**1a**) in DMF.

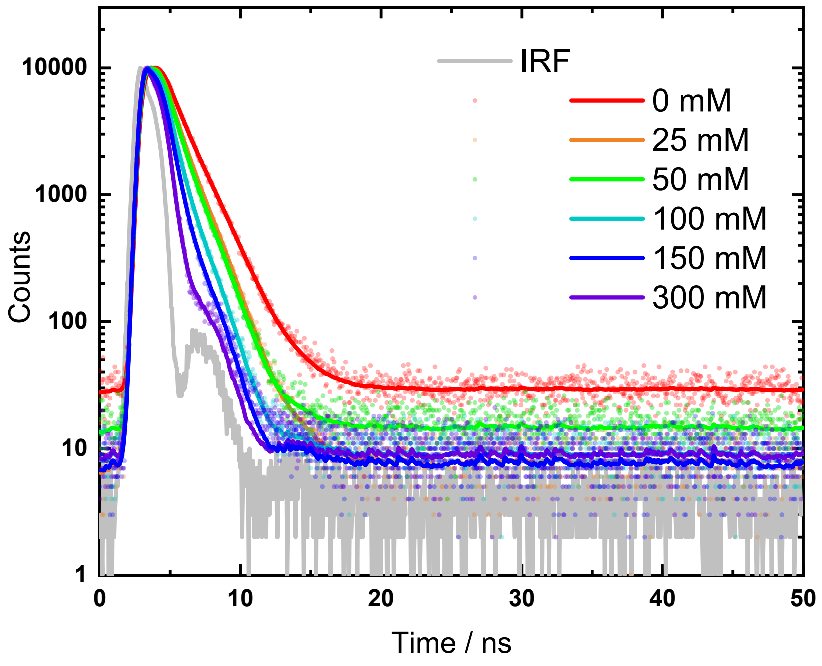

Figure S9: TCSPC measurements of quenching of [Fe(III)(phtmeimb)$_2$]PF$_6$ by substrate (**1a**) in DMF.

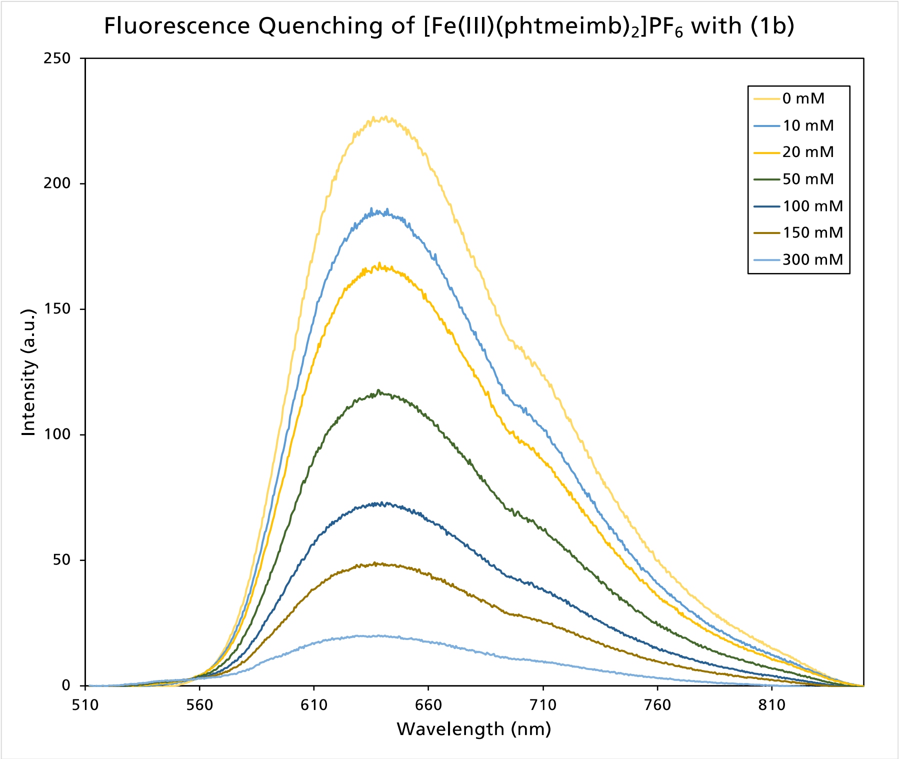

Figure S10: Emission quenching of [Fe(III)(phtmeimb)$_2$]PF$_6$ by substrate (**1b**) in DMF.

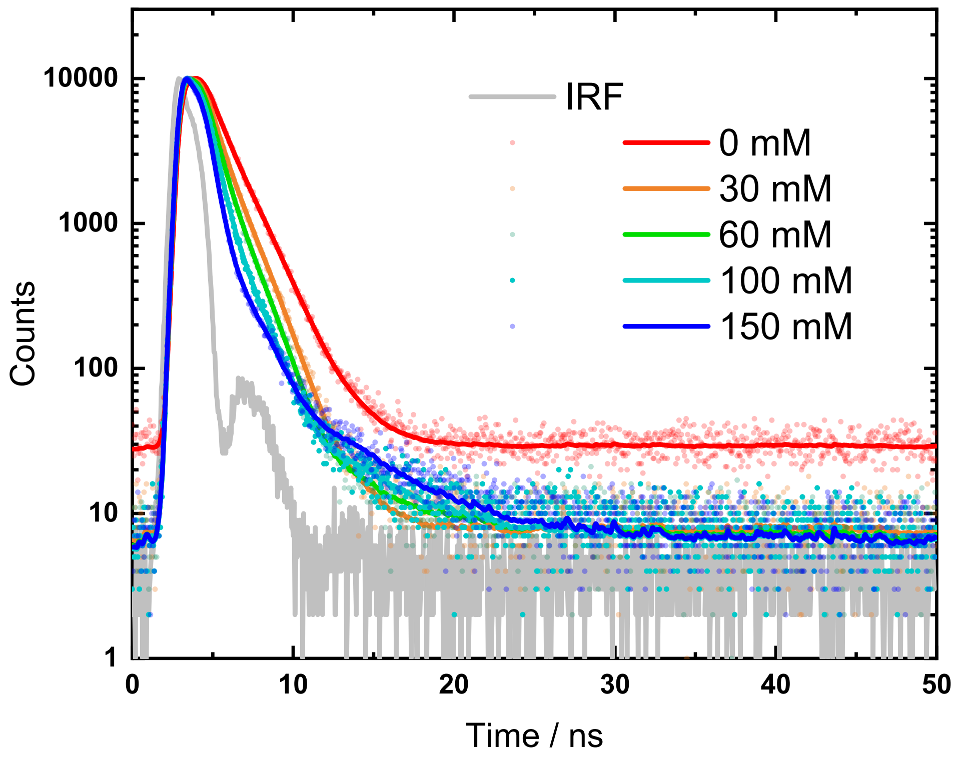

Figure S11: TCSPC measurements of quenching of [Fe(III)(phtmeimb)$_2$]PF$_6$ by substrate (**1b**) in DMF.

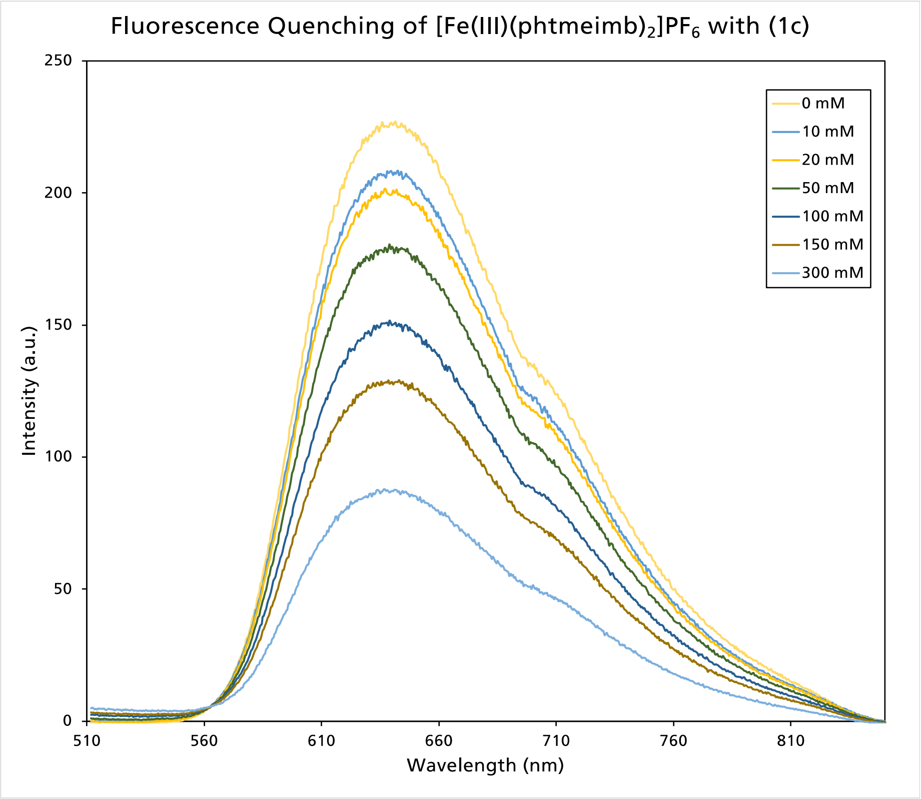

Figure S12: Emission quenching of [Fe(III)(phtmeimb)$_2$]PF$_6$ by substrate (**1c**) in DMF.

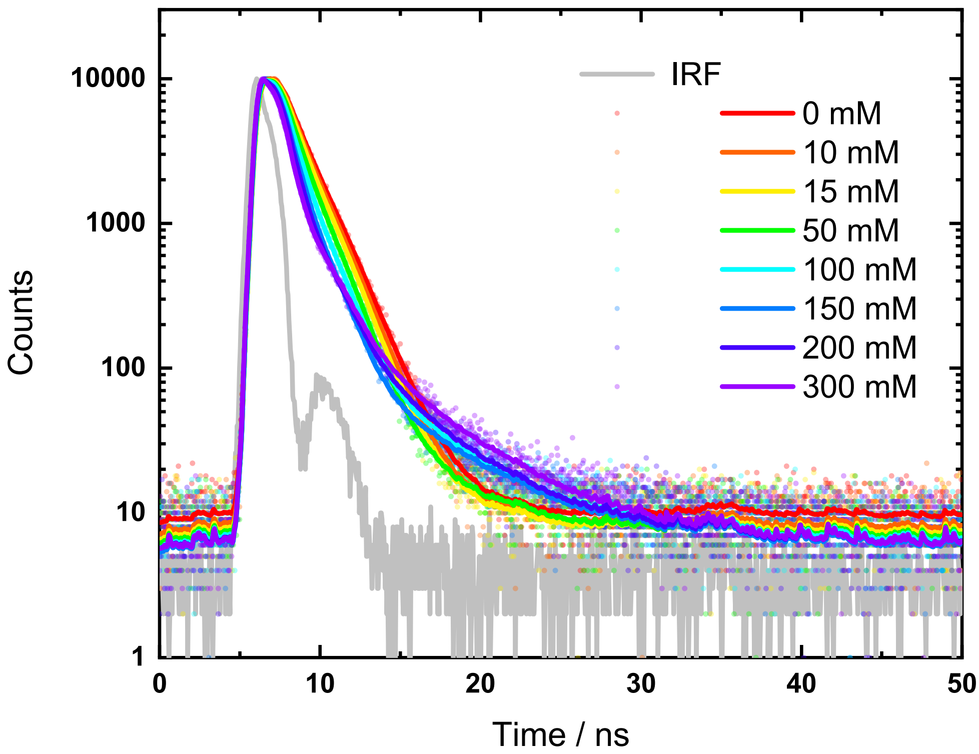

Figure S13: TCSPC measurements of quenching of [Fe(III)(phtmeimb)$_2$]PF$_6$ by substrate (**1c**) in DMF.

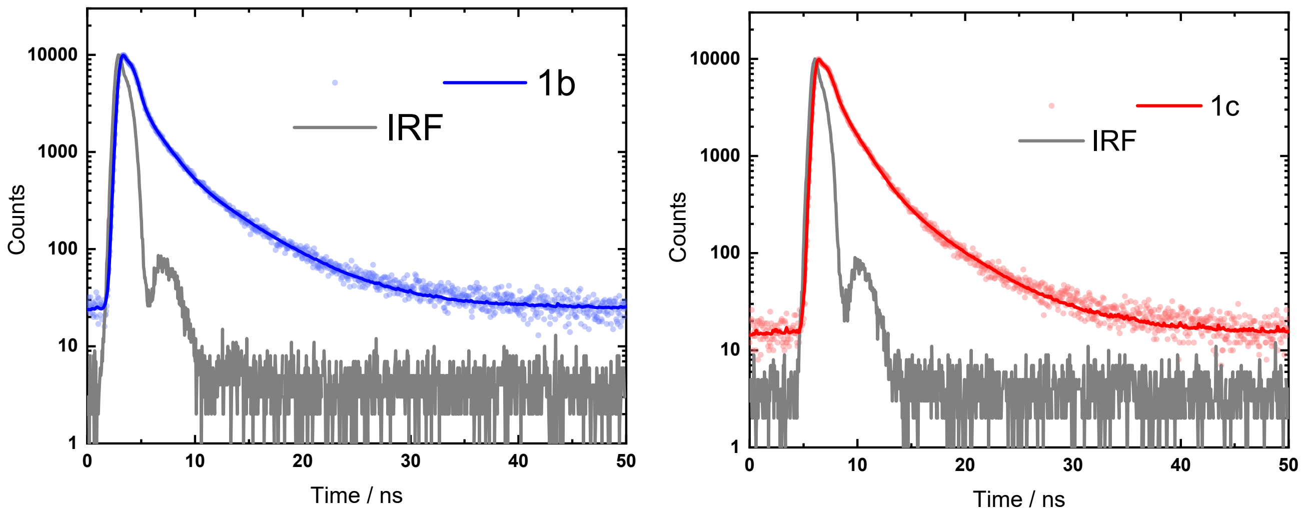

Figure S14: TCSPC measurements of neat (**1b**) and (**1c**) in DMF.

With excitation at 470 nm, substrates **(1b)** and **(1c)** could also be excited. Hence lifetime measurements of neat samples of both **(1b)** and **(1c)** under the same experimental conditions were conducted. This gave rise to lifetimes with weighted averages of 2.14 ns (1.45 (83 %) and 5.52 (17 %) ns) and 2.00 ns (1.59 (89 %) and 5.28 (11 %) ns), respectively. These lifetimes were fixed while trying to fit the TCSPC decay curves of the quenching studies to yield the lifetime of [Fe(III)(phtmeimb)$_2$]PF$_6$.

Due to the combination of static and dynamic quenching observed for (**1a**) and (**1b**), polynomial fits of the data obtained from steady-state emission quenching were performed. This quadratic dependence is described in Eq. 1, where I_0 is the emission intensity without quencher Q present, I is the emission intensity in presence of the quencher, K_D (M^{-1}) corresponds to the dynamic quenching constant, K_S (M^{-1}) to the static quenching constant and [Q] (M) to the quencher concentration. Introduction of K_{app}, the apparent quenching constant, (Eq. 2), this quadratic expression can be simplified and thus utilised to graphically separate K_S and K_D. By plotting K_{app}/[Q] against [Q] a linear plot is obtained where K_D+K_S corresponds to the intercept A and K_DK_S to the slope B (**Figure S15** & **Figure S16**). As the K_D values had been obtained from the TCSPC data, the K_S were obtained from the two solutions of the quadratic expression in Eq. 3, with the solution most consistent with the K_D value.

$$\frac{I_0}{I} = 1 + (K_D + K_S)[Q] + K_D K_S [Q]^2 = 1 + K_{app}[Q] \qquad \text{(Eq. 1)}$$

$$K_{app} = (K_D + K_S) + K_D K_S [Q] = \frac{I_0}{I} - 1 \qquad \text{(Eq. 2)}$$

$$K_S^2 - K_S A + B = 0 \qquad \text{(Eq. 3)}$$

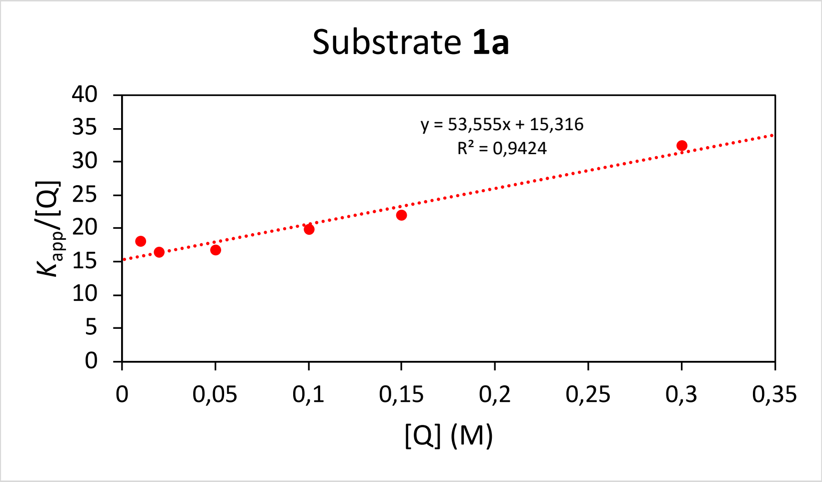

Figure S15: Plot of K_{app}/[Q] vs [Q] for the graphical separation of K_S and K_D of substrate (**1a**) using the intercept A and the slope B.

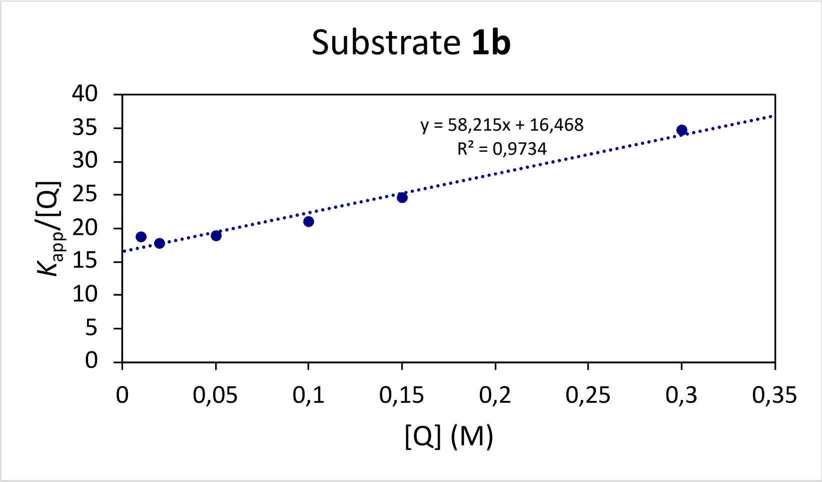

Figure S16: Plot of K_{app}/[Q] vs [Q] for the graphical separation of K_S and K_D of substrate (**1a**) using the intercept A and the slope B.

Nanosecond transient absorption measurements

Nanosecond transient absorption measurements were employed to measure the cage escape yields (CEYs) of the reductive quenching process and to probe the recovery of [Fe(III)(phtmeimb)$_2$]PF$_6$ through the oxidation of the Fe(II) species.

Cage Escape Yield Measurements

For CEY measurements, the quenchers were dissolved in spectroscopic grade DMF (≥99.8%, Merck) with concentrations of 104 mM (Substrate **1a**), 101 mM (Substrate **1b**) and 88 mM (Substrate **1c**). A solution of [Ru(bpy)$_3$](PF$_6$)$_2$ in DMF matching the absorbance of [Fe(phtmeimb)$_2$]PF$_6$ at the excitation wavelength and excited with the same energy was used as actinometer for the determination of the cage escape yields. Cage escape yields of electron transfer products were calculated using the transient absorption of the Fe(III)→Fe(II) reduction of [Fe(phtmeimb)$_2$]$^+$ ($\Delta\varepsilon$ = 8724 M^{-1}cm^{-1} at 375 nm, in acetonitrile)[1]and of the ^{3}MLCT excited state of [Ru(bpy)$_3$]$^{2+}$ ($\Delta\varepsilon$ = -1.1×10^4 M^{-1}cm^{-1} at 452 nm, in acetonitrile).[9] The differences in extinction coefficients for [Fe(phtmeimb)$_2$]$^+$ and [Ru(bpy)$_3$]$^{2+}$ in DMF are not known, hence it is hereby assumed that the differences are similar to those for acetonitrile while determining the cage escape yields for experiments in DMF.

Table S3: Calculations for cage escape yields of quenching products. [a] Sample absorbance at the excitation wavelength (465 nm), [b] Photo-induced absorbance change of the sample at 375 nm, [c] Photo-generated concentration of Fe(III/II) based on $\Delta\varepsilon$ = 8724 $M^{-1}cm^{-1}$ at 375 nm in acetonitrile, [d] Actinometer absorbance at the excitation wavelength, [e] Photo-induced absorbance change of the actinometer at 452 nm, [f] Photo generated concentration of $*[Ru(bpy)_3]^{2+}$ based on $\Delta\varepsilon$ = -1.1×10^4 $M^{-1}cm^{-1}$ at 452 nm, [g] Correction factor for absorbance difference between sample and actinometer $f = \left(1-10^{-A_{Ru}(\lambda_{ex})}\right)/\left(1-10^{-A_{Fe}(\lambda_{ex})}\right)$, [h] Quantum yield of electron transfer products $\phi = (\Delta[Fe]/\Delta[Ru])f$, [i] Quenching yield from steady state emission quenching, [j] Cage escape yield $\eta_{ce} = \phi/\eta_q$

Quencher	$A_{Fe}(\lambda_{ex})$[a]	ΔA_{Fe} (375)[b]	Δ[Fe]/M [c]	$A_{Ru}(\lambda_{ex})$[d]	ΔA_{Ru} (452)[e]	Δ[Ru]/M[f]	f[g]	ϕ ([Q]/mM)[h]	η_q[i]	η_{ce}[j]
Substrate **1a**	0.362	0.0122	1.40×10^{-6}	0.356	−0.148	1.35×10^{-5}	0.99	0.103 (104)	0.67	0.15
Substrate **1b**	0.362	0.0183	2.10×10^{-6}				0.99	0.154 (101)	0.69	0.22
Substrate **1c**	0.362	0.0036	4.13×10^{-7}				0.99	0.030 (88)	0.43	0.09

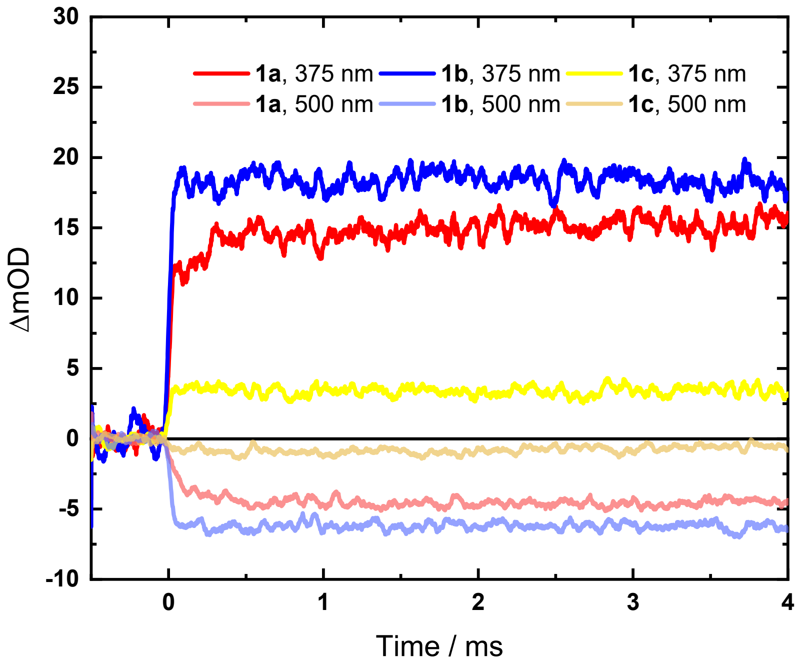

Figure S17: Nanosecond transient absorption kinetic traces at indicated wavelengths of the substrates in DMF.

Recovery of [Fe(III)(phtmeimb)$_2$]PF_6

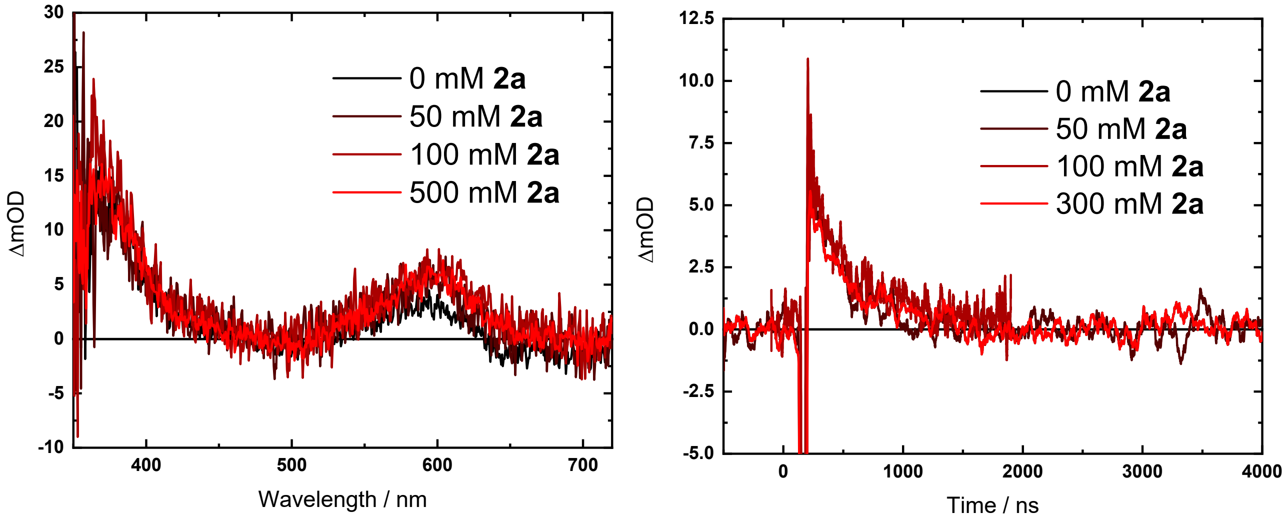

Figure S18: Left: ns-TAS spectra at 50 ns time delay of [Fe(III)(phtmeimb)$_2$]$^+$ (0.16 mM) and substrate **1a** (100 mM), with varying concentrations of **2a**. Right: ns-TAS kinetic traces at 600 nm of [Fe(III)(phtmeimb)$_2$]$^+$ (0.16 mM) and substrate **1a** (100 mM), with varying concentrations of **2a**.

NMR Spectra

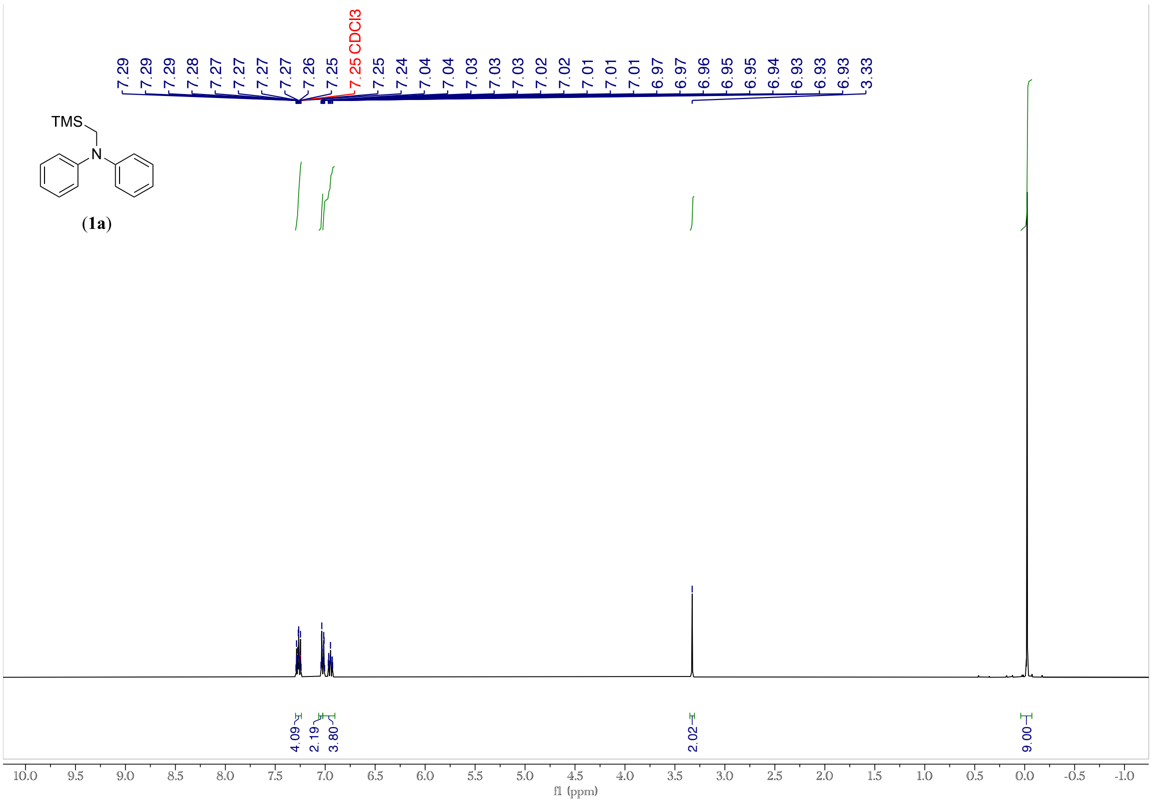

F

igure S19: ^{1}H NMR spectrum (400 MHz) of **(1a)** in $CDCl_3$.

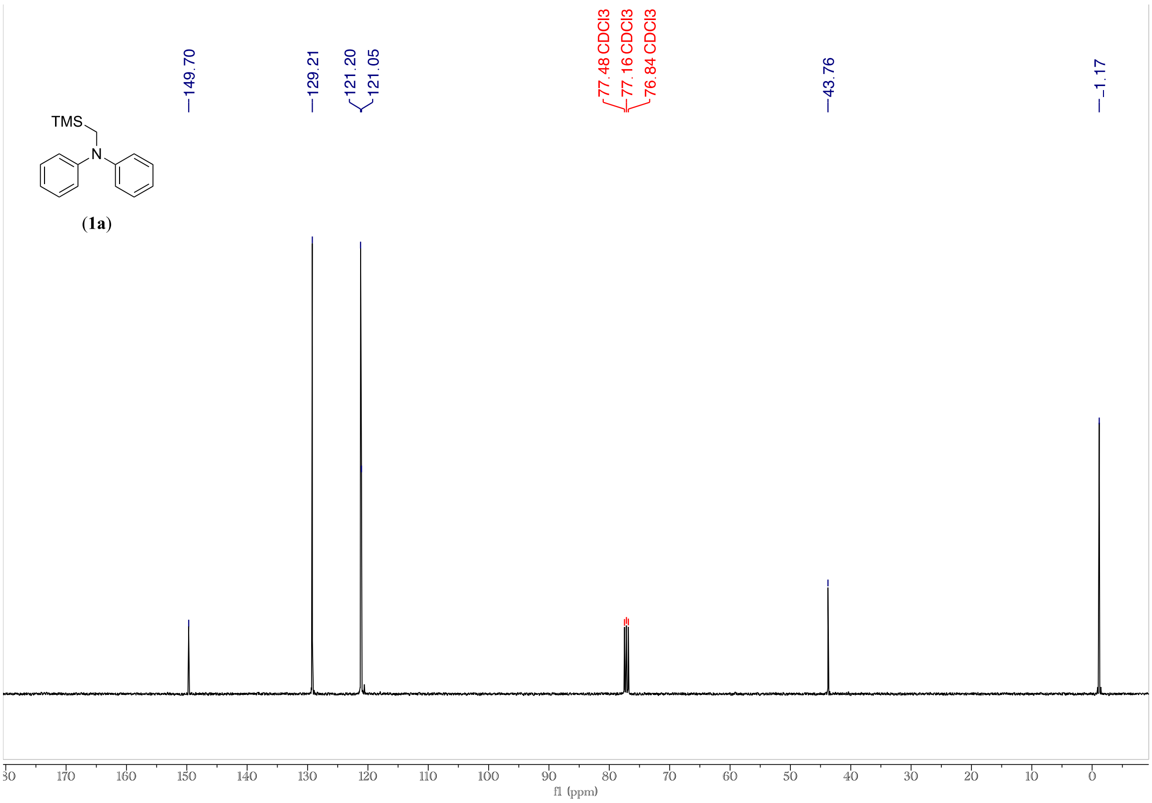

F

igure S20: ^{13}C NMR spectrum (101 MHz) of **(1a)** in $CDCl_3$.

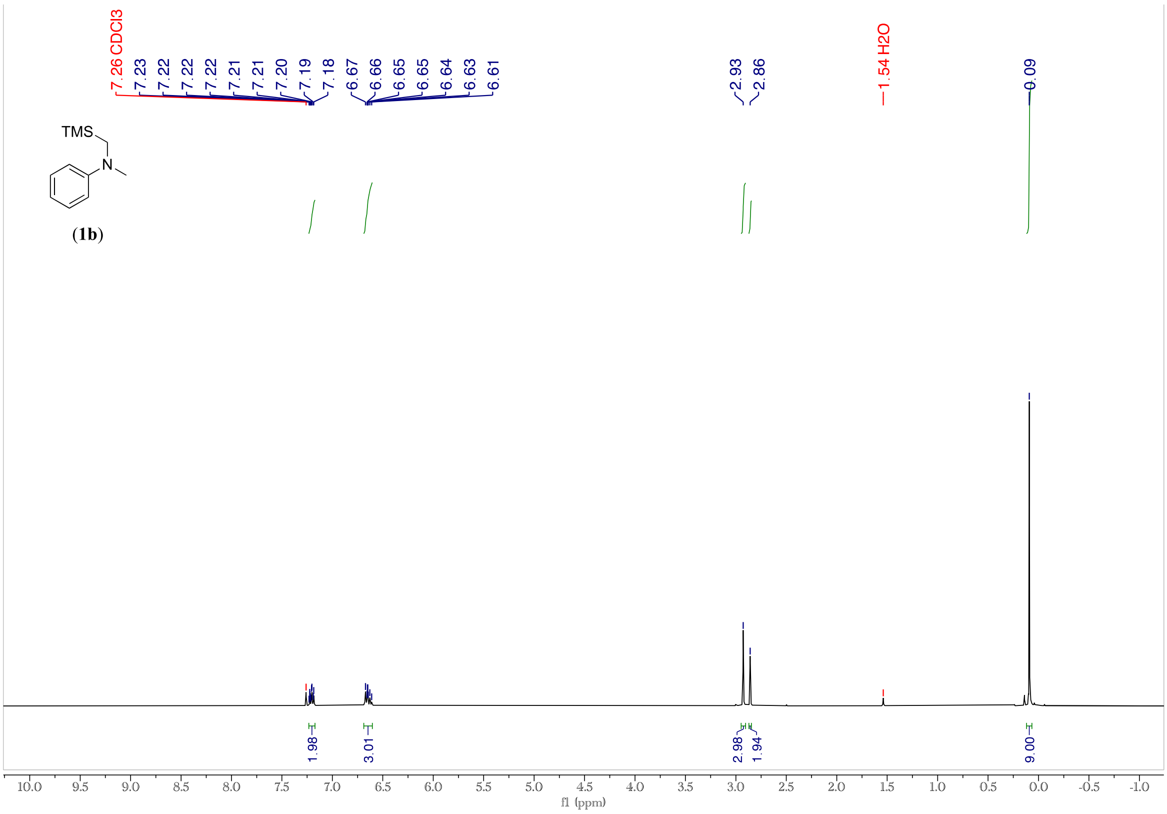

Figure S21: ^{1}H NMR spectrum (400 MHz) of **(1b)** in $CDCl_3$.

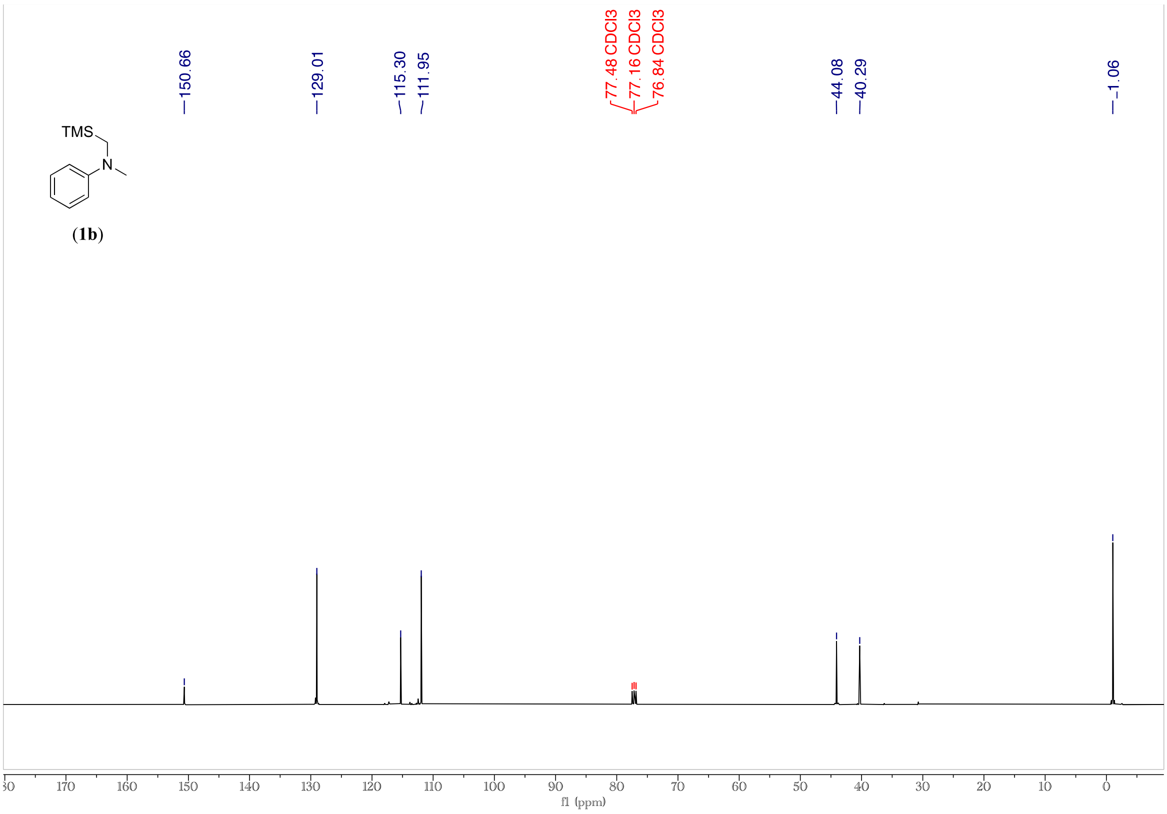

Figure S22: ^{13}C NMR spectrum (101 MHz) of **(1b)** in $CDCl_3$.

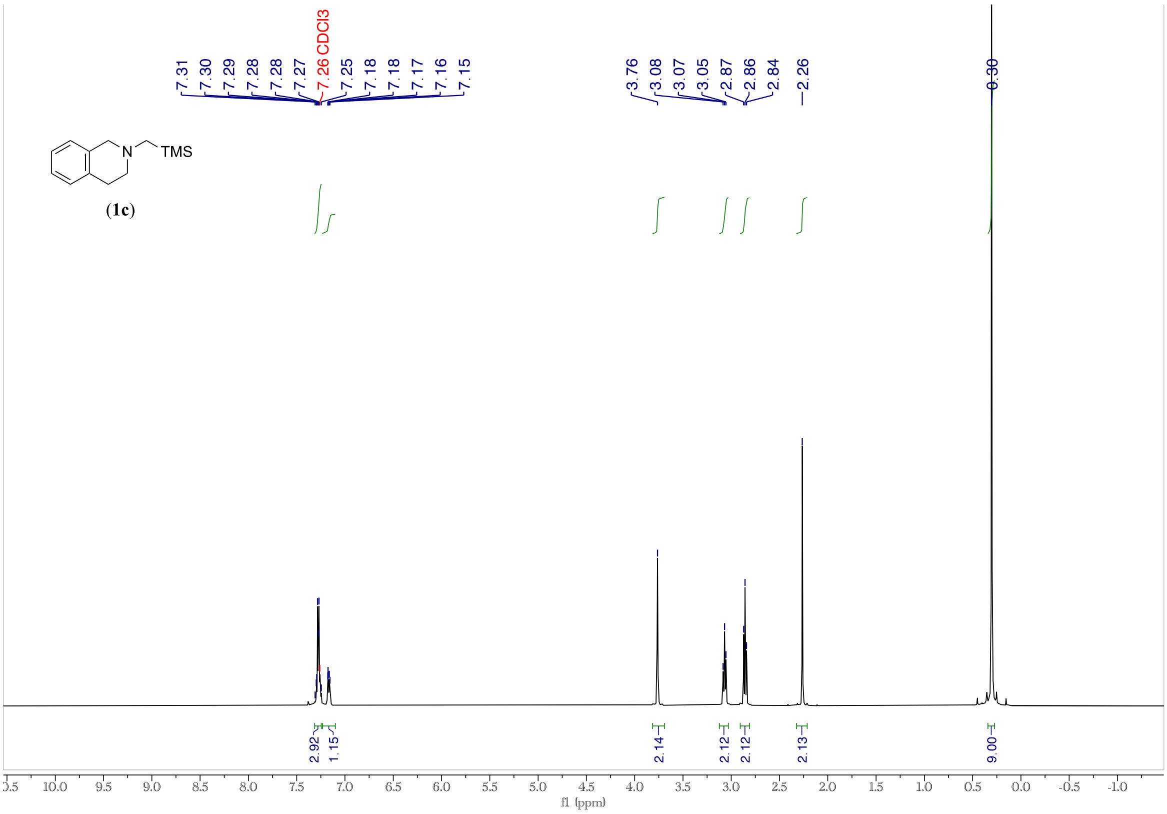

Figure S23: ^{1}H NMR spectrum (400 MHz) of **(1c)** in $CDCl_3$.

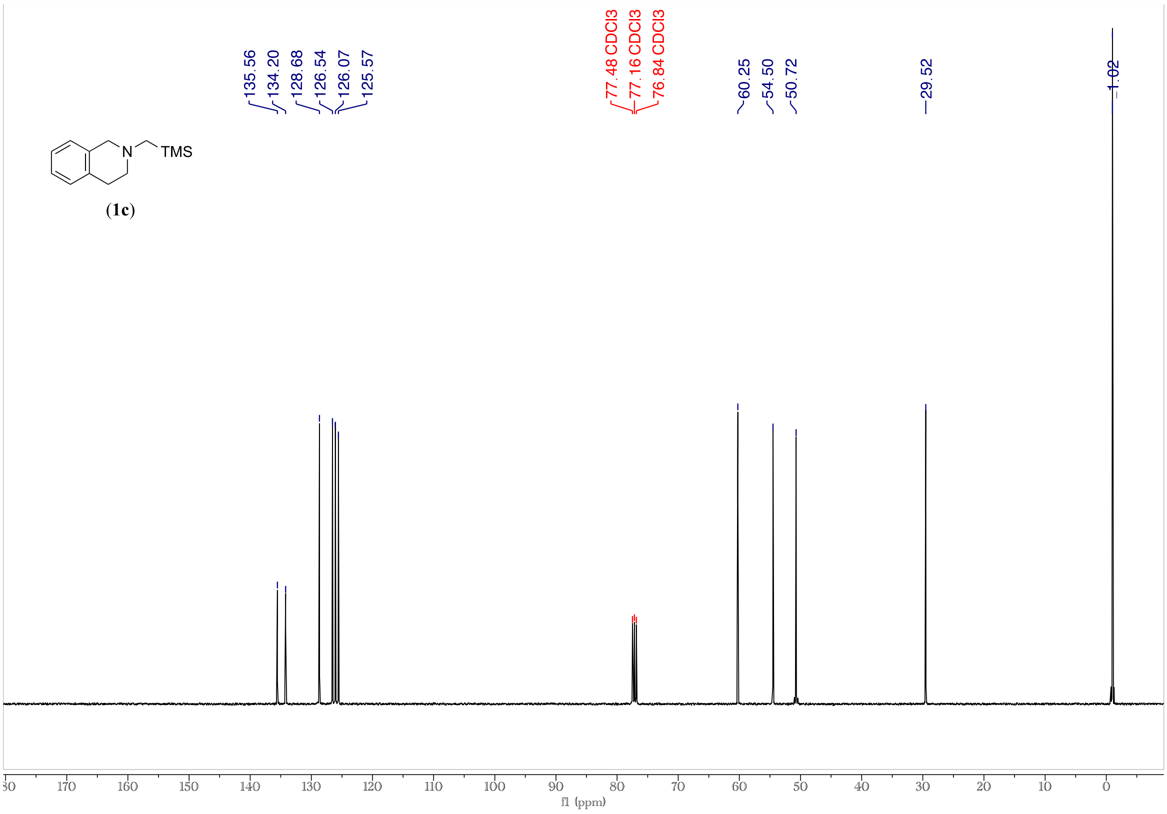

Figure S24: ^{13}C NMR spectrum (101 MHz) of **(1c)** in $CDCl_3$.

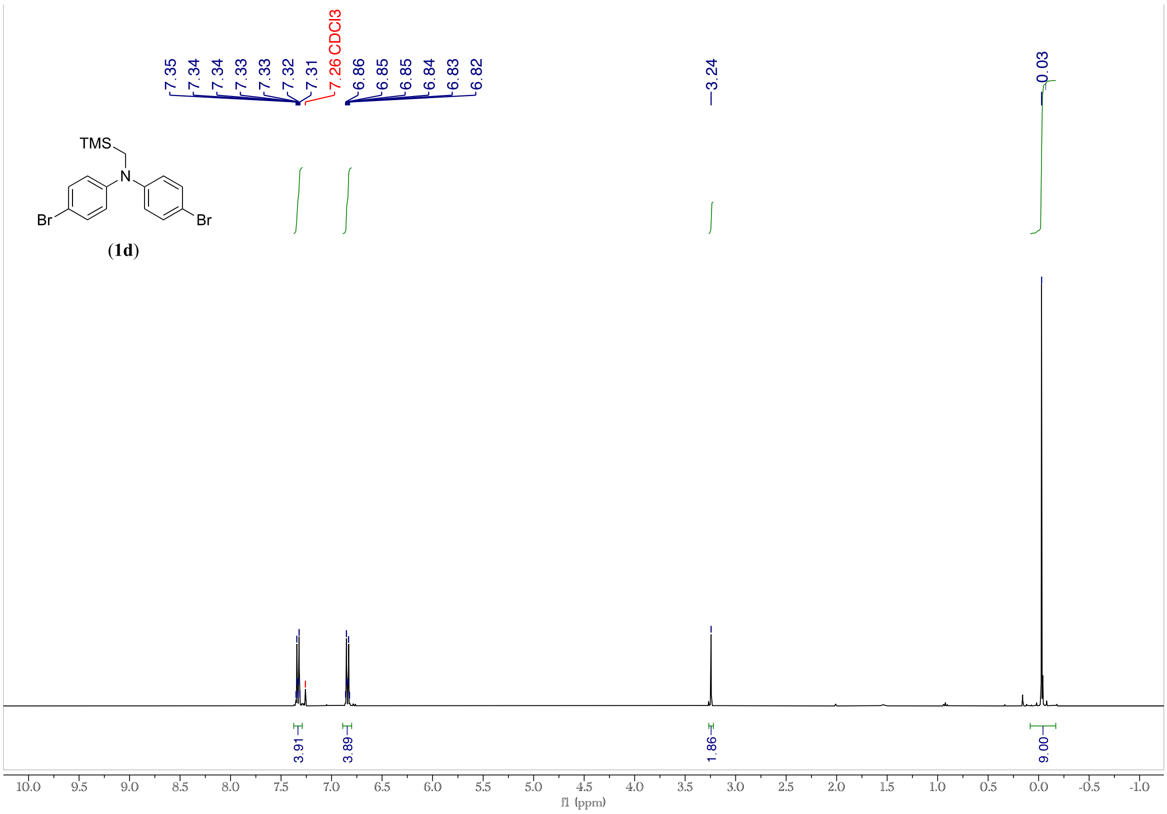

Figure S25: ^{1}H NMR spectrum (400 MHz) of **(1d)** in $CDCl_3$.

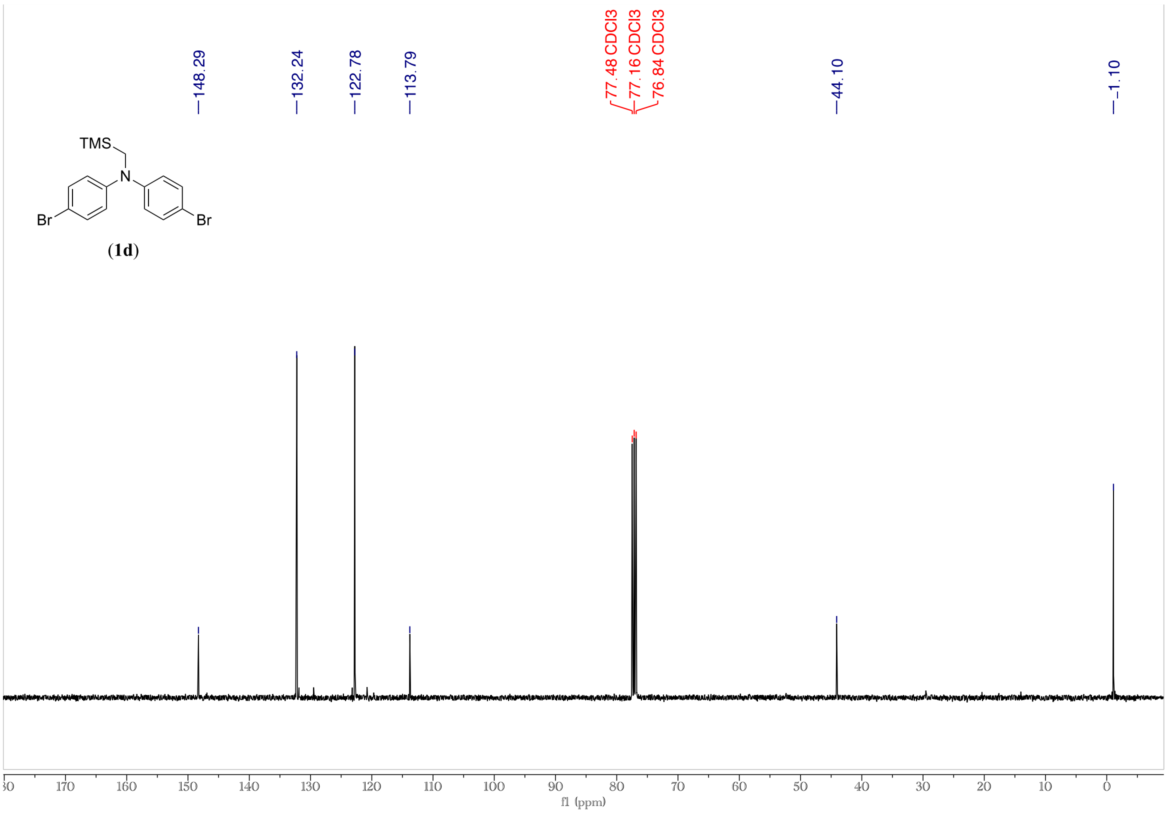

Figure S26: ^{13}C NMR spectrum (101 MHz) of **(1d)** in $CDCl_3$.

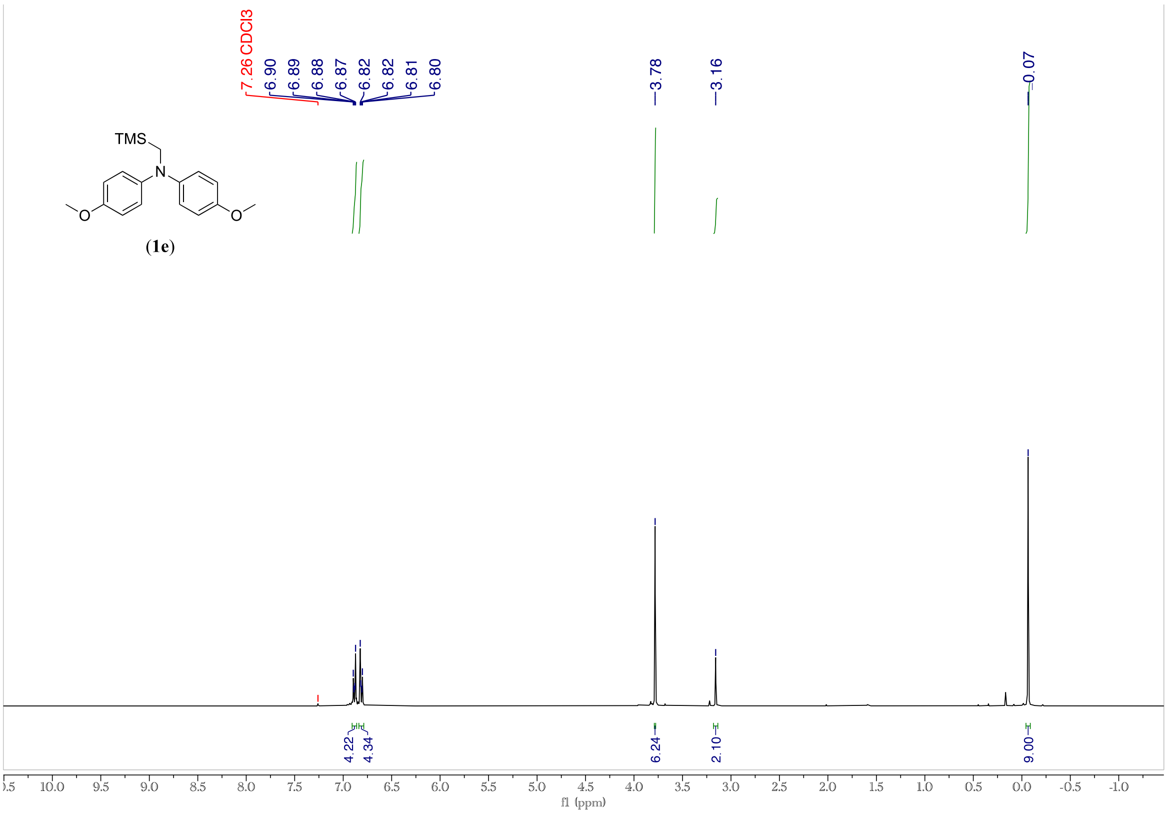

Figure S27: ^{1}H NMR spectrum (400 MHz) of **(1e)** in $CDCl_3$.

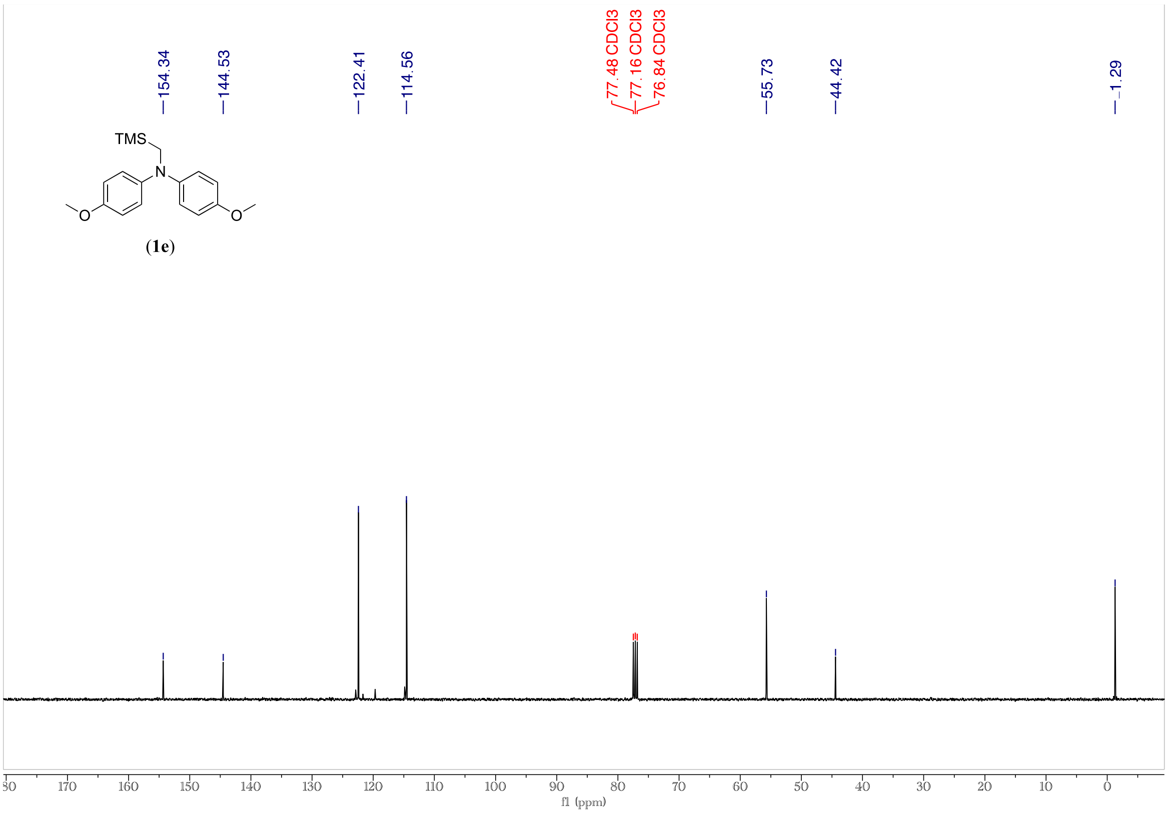

Figure S28: ^{13}C NMR spectrum (101 MHz) of **(1e)** in $CDCl_3$.

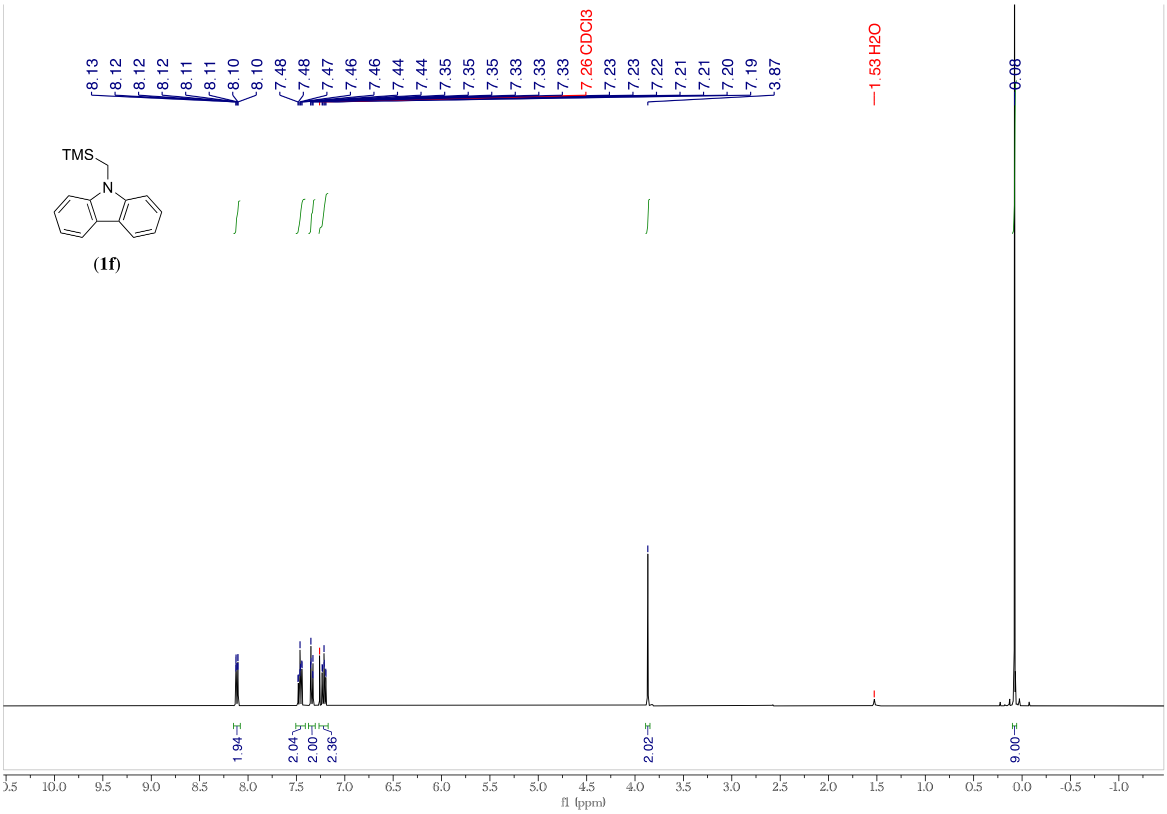

Figure S29: ^{1}H NMR spectrum (400 MHz) of **(1f)** in $CDCl_3$.

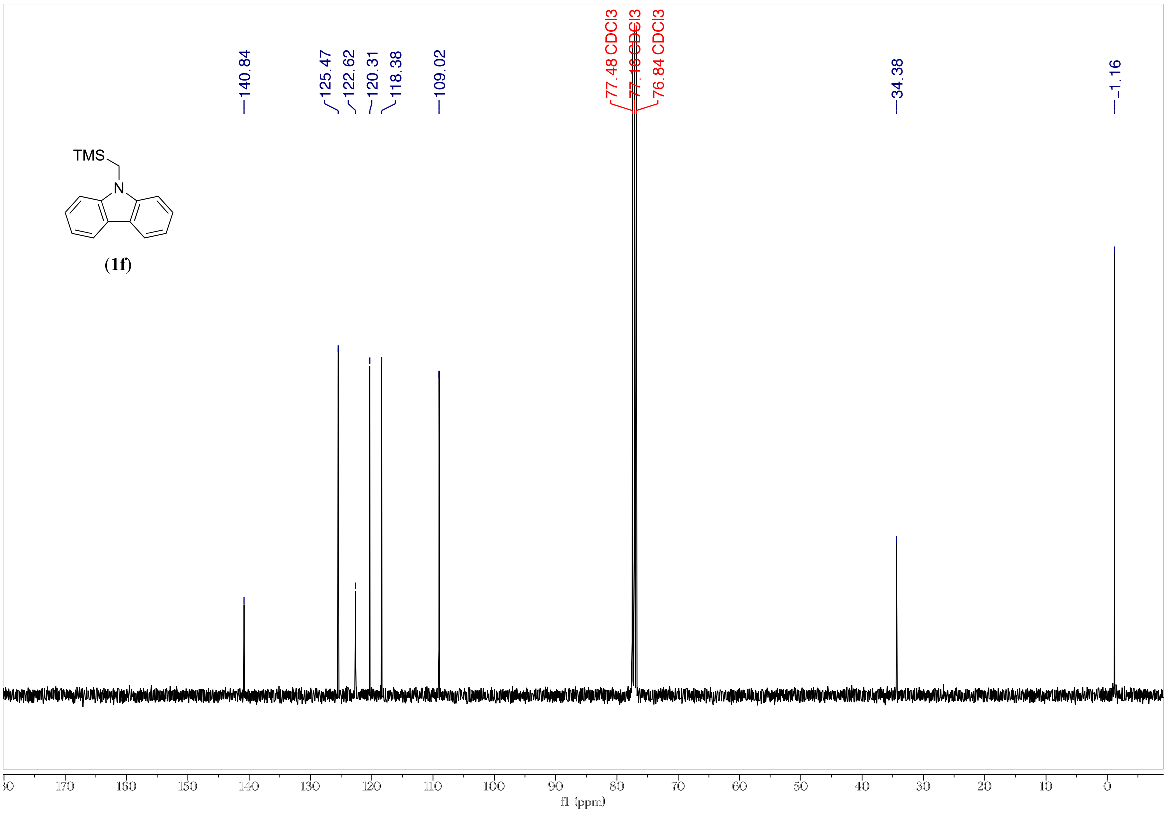

Figure S30: ^{13}C NMR spectrum (101 MHz) of **(1f)** in $CDCl_3$.

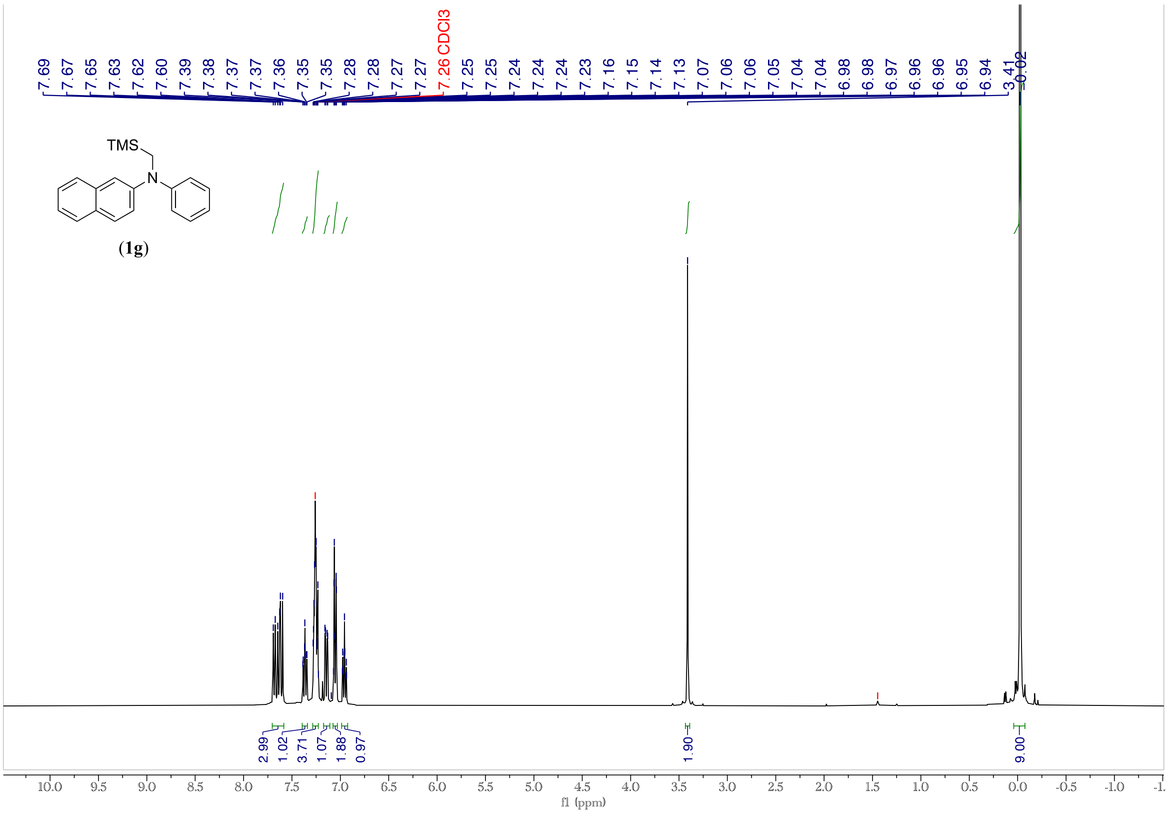

Figure S31: ^{1}H NMR spectrum (400 MHz) of **(1g)** in $CDCl_3$.

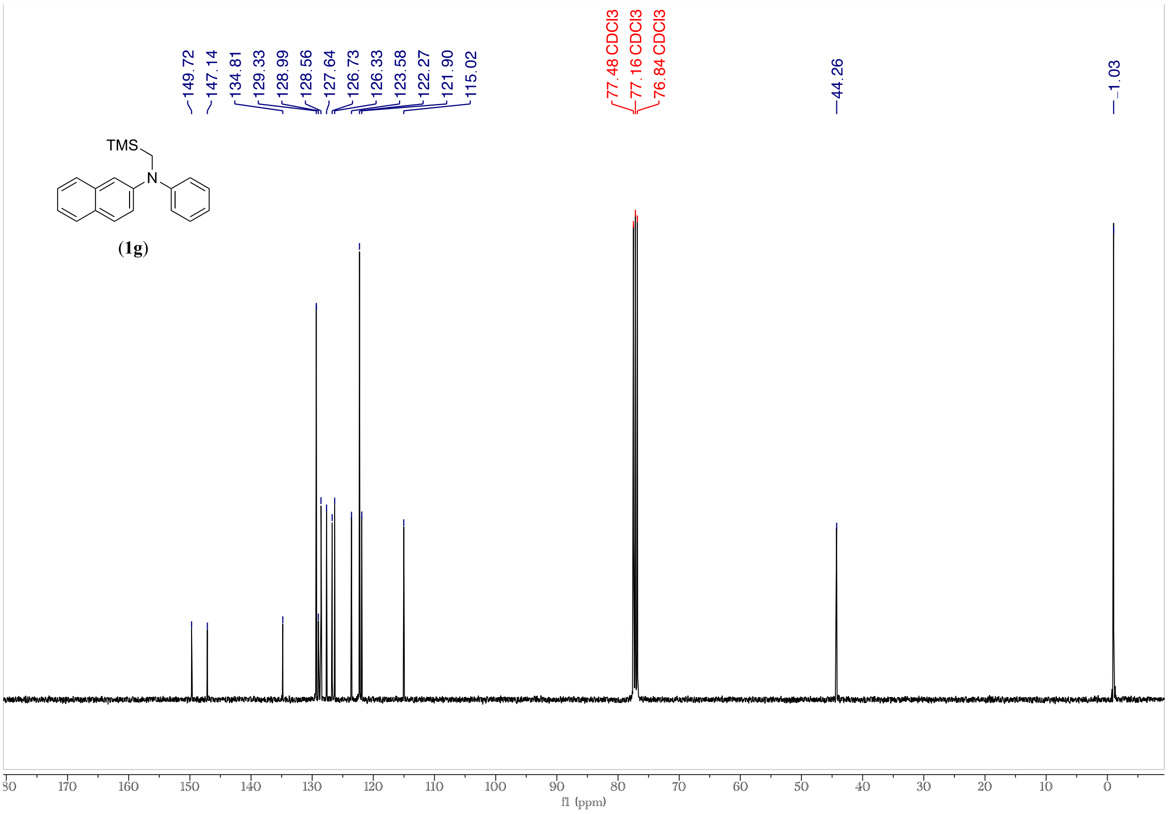

Figure S32: ^{13}C NMR spectrum (101 MHz) of **(1g)** in $CDCl_3$.

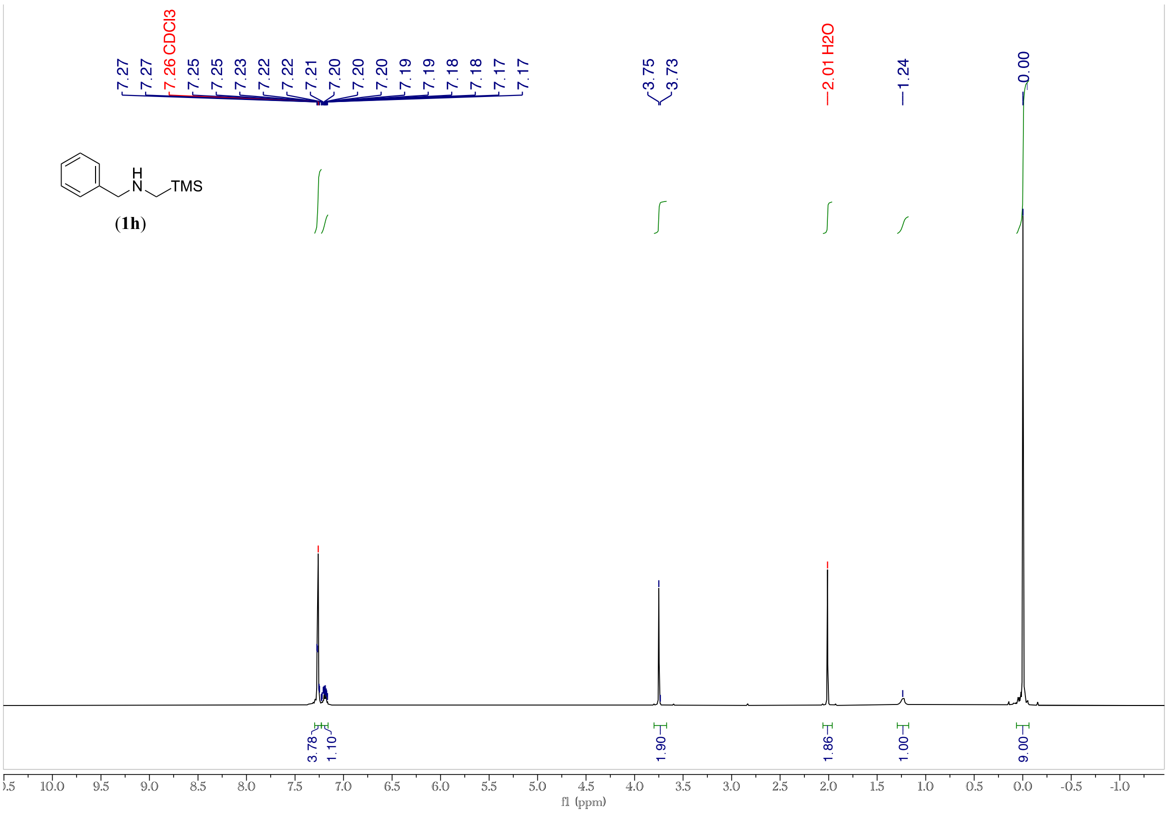

Figure S33: ^{1}H NMR spectrum (400 MHz) of **(1h)** in $CDCl_3$.

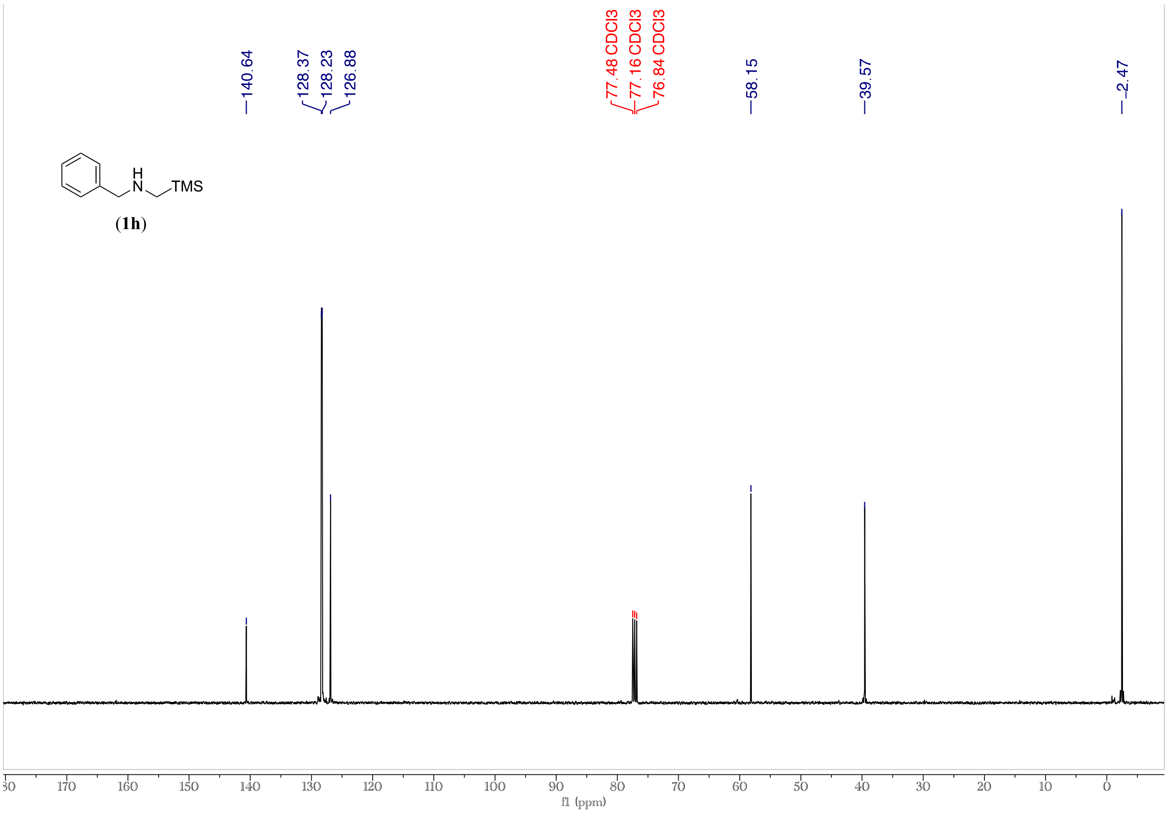

Figure S34: ^{13}C NMR spectrum (101 MHz) of **(1h)** in $CDCl_3$.

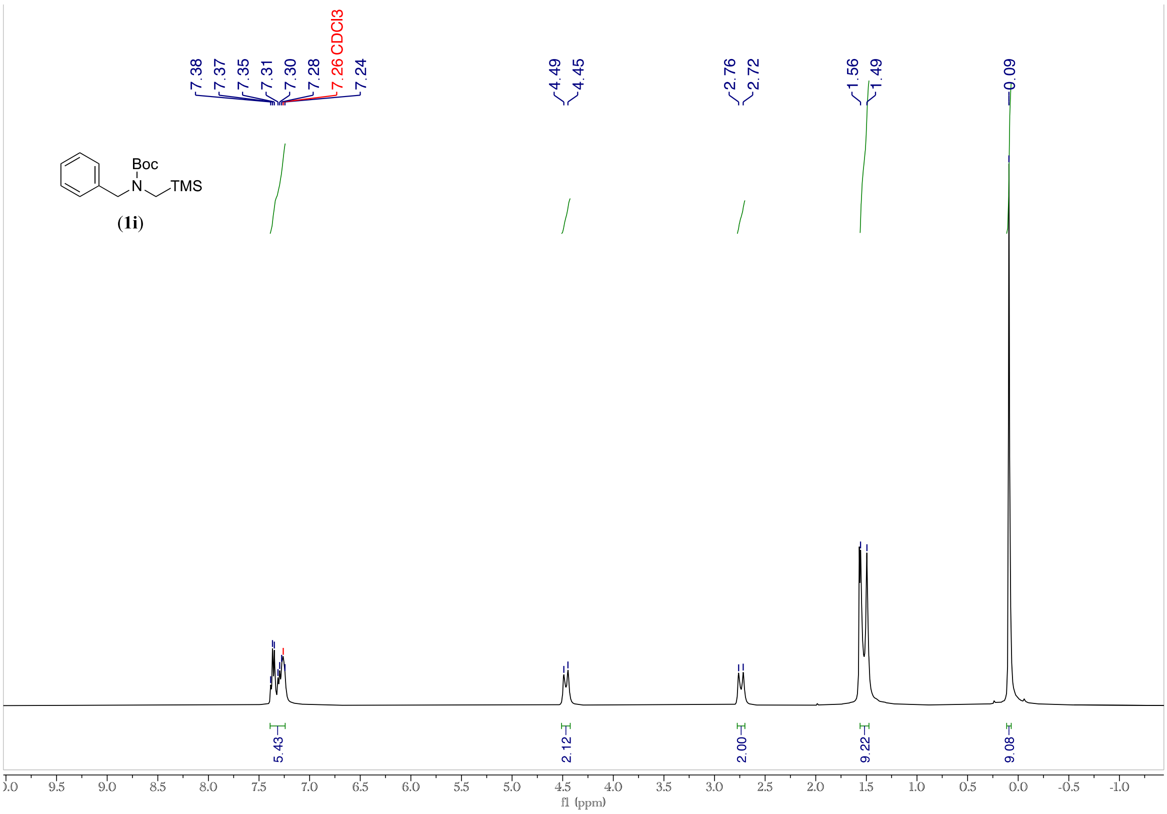

Figure S35: ^{1}H NMR spectrum (400 MHz) of **(1i)** in $CDCl_3$.

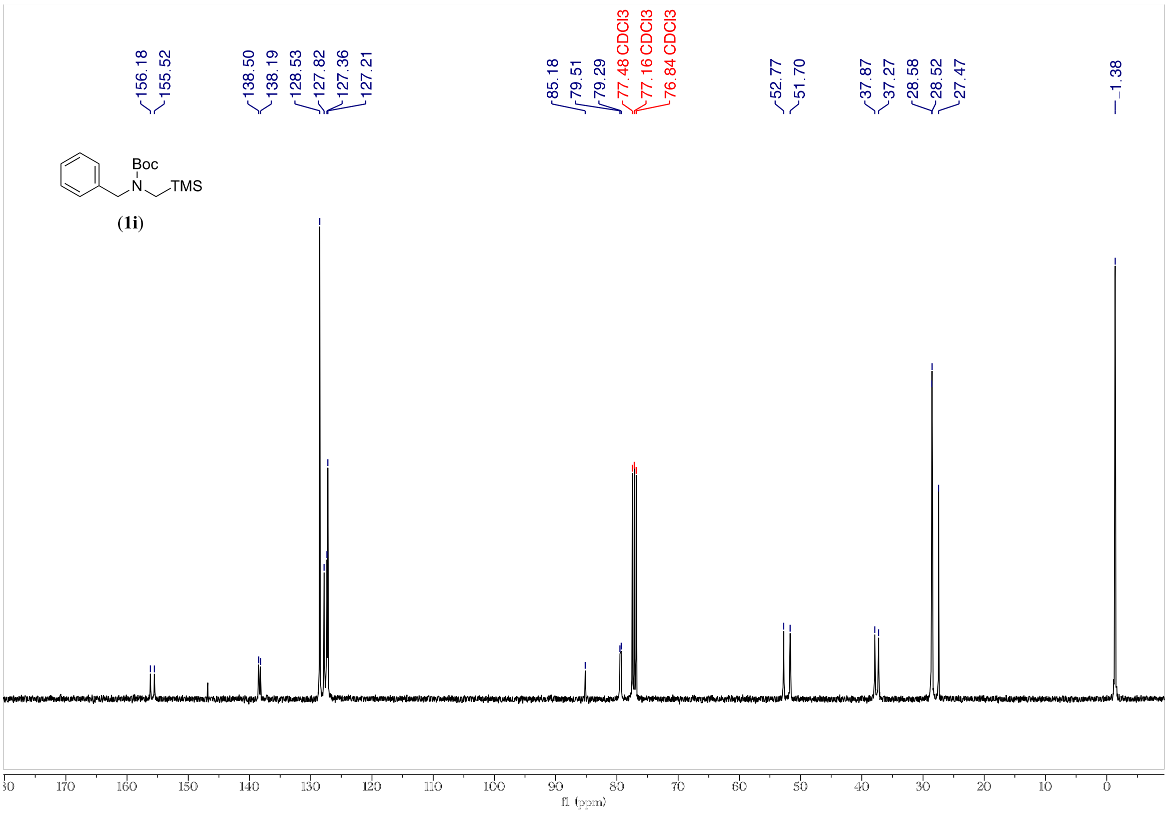

Figure S36: ^{13}C NMR spectrum (101 MHz) of **(1i)** in $CDCl_3$.

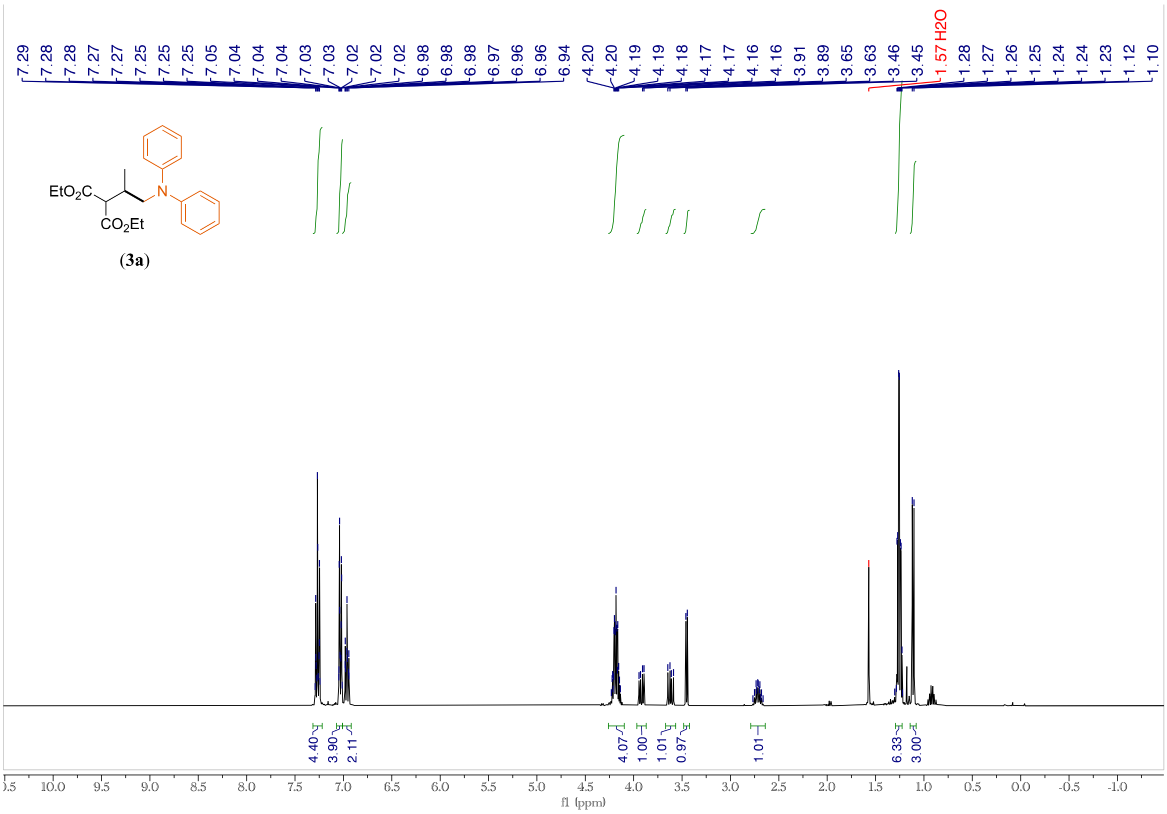

Figure S37: ^{1}H NMR spectrum (400 MHz) of **(3a)** in $CDCl_3$.

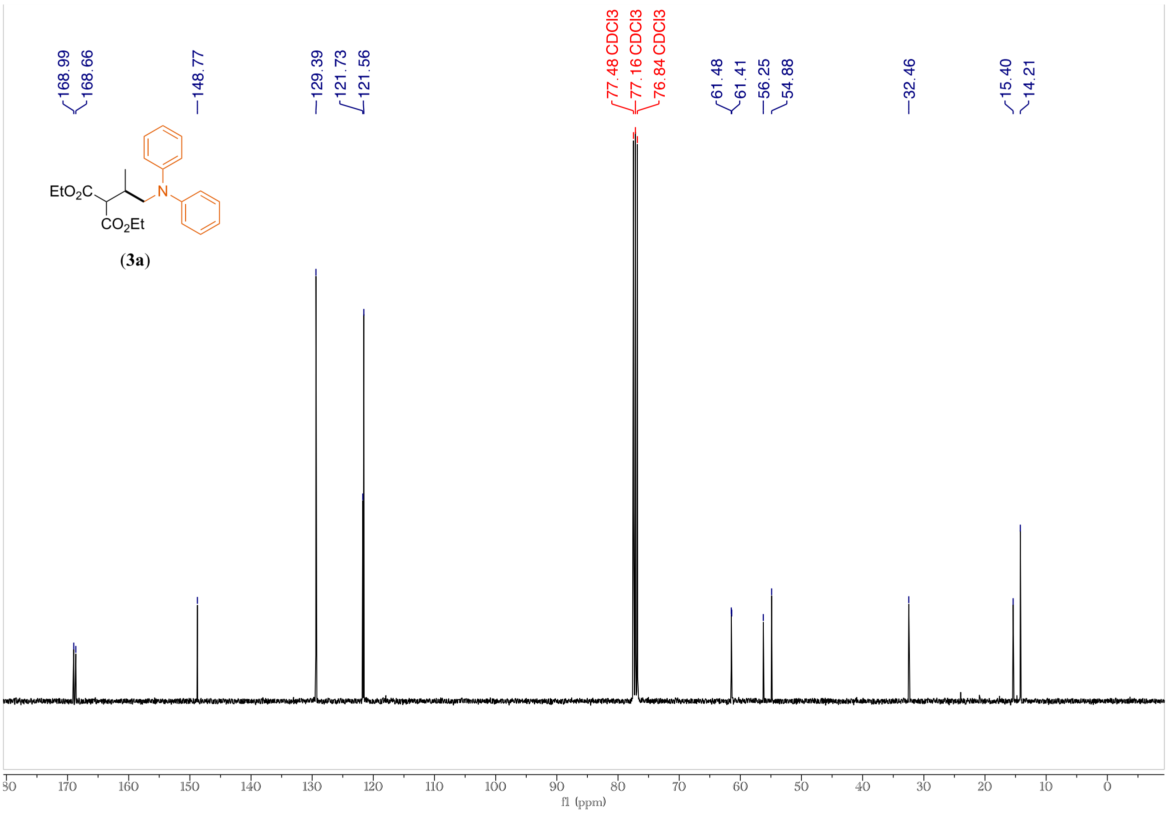

Figure S38: ^{13}C NMR spectrum (101 MHz) of (**3a**) in CDCl3.

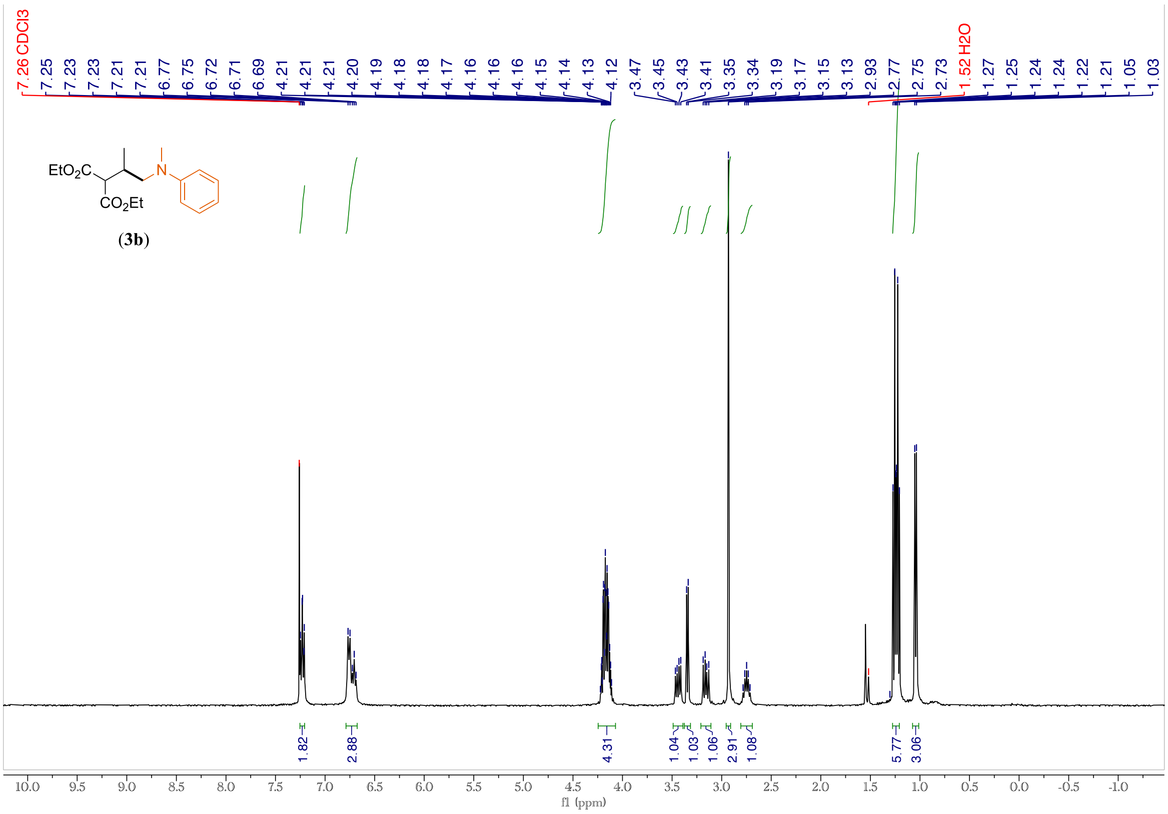

Figure S39: ^{1}H NMR spectrum (400 MHz) of **(3b)** in $CDCl_3$.

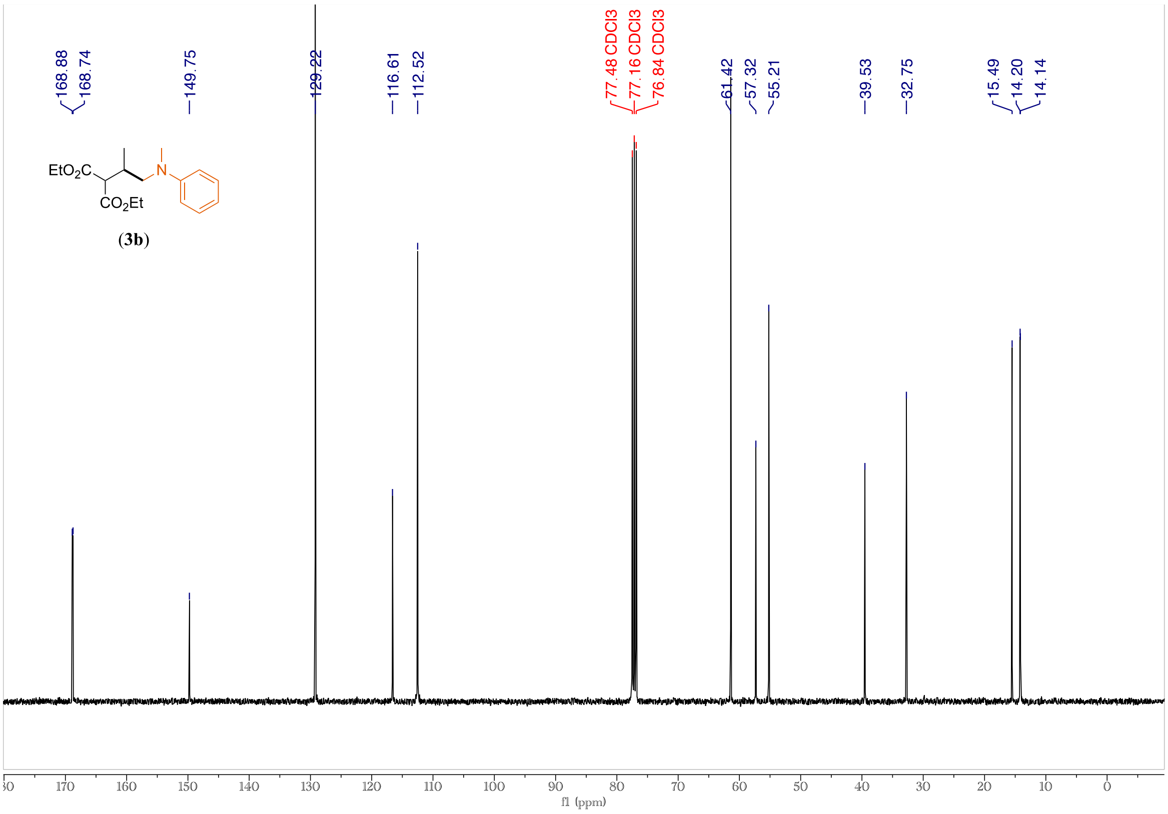

Figure S40: ^{13}C NMR spectrum (101 MHz) of (**3b**) in CDCl3.

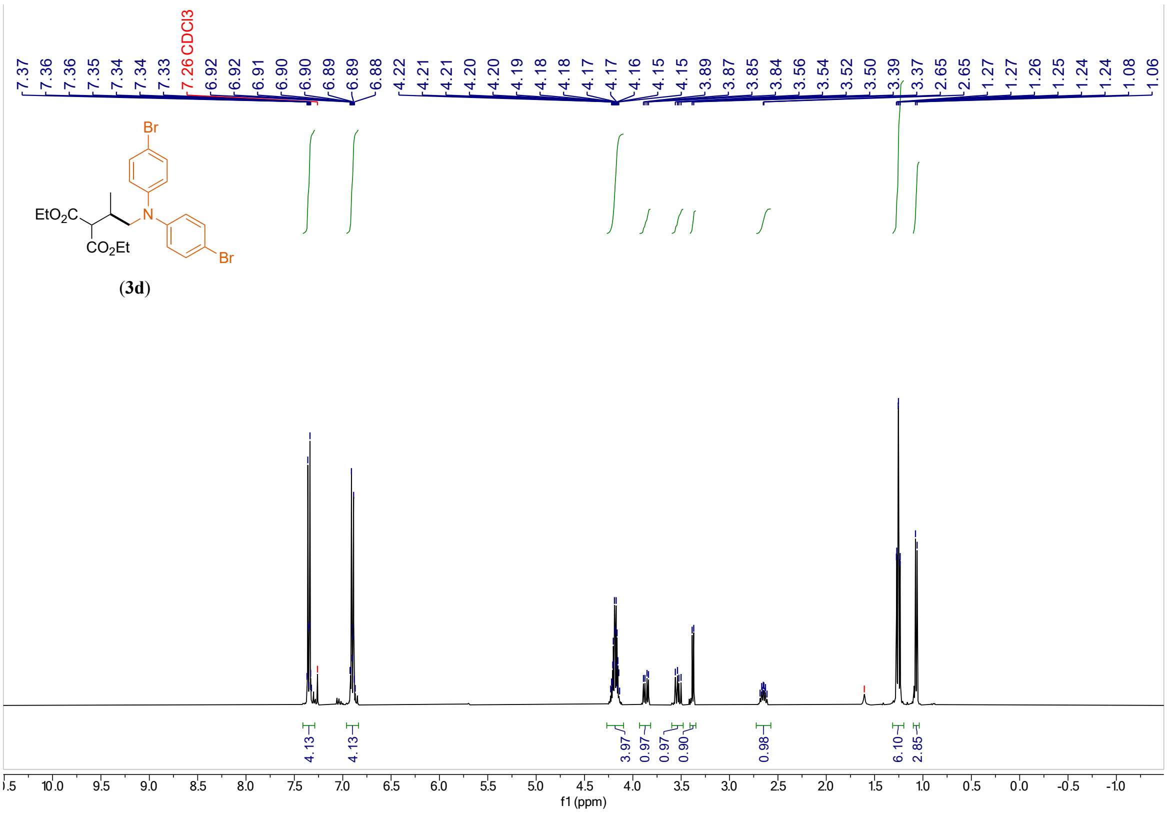

Figure S41: ^{1}H NMR spectrum (400 MHz) of **(3d)** in $CDCl_3$.

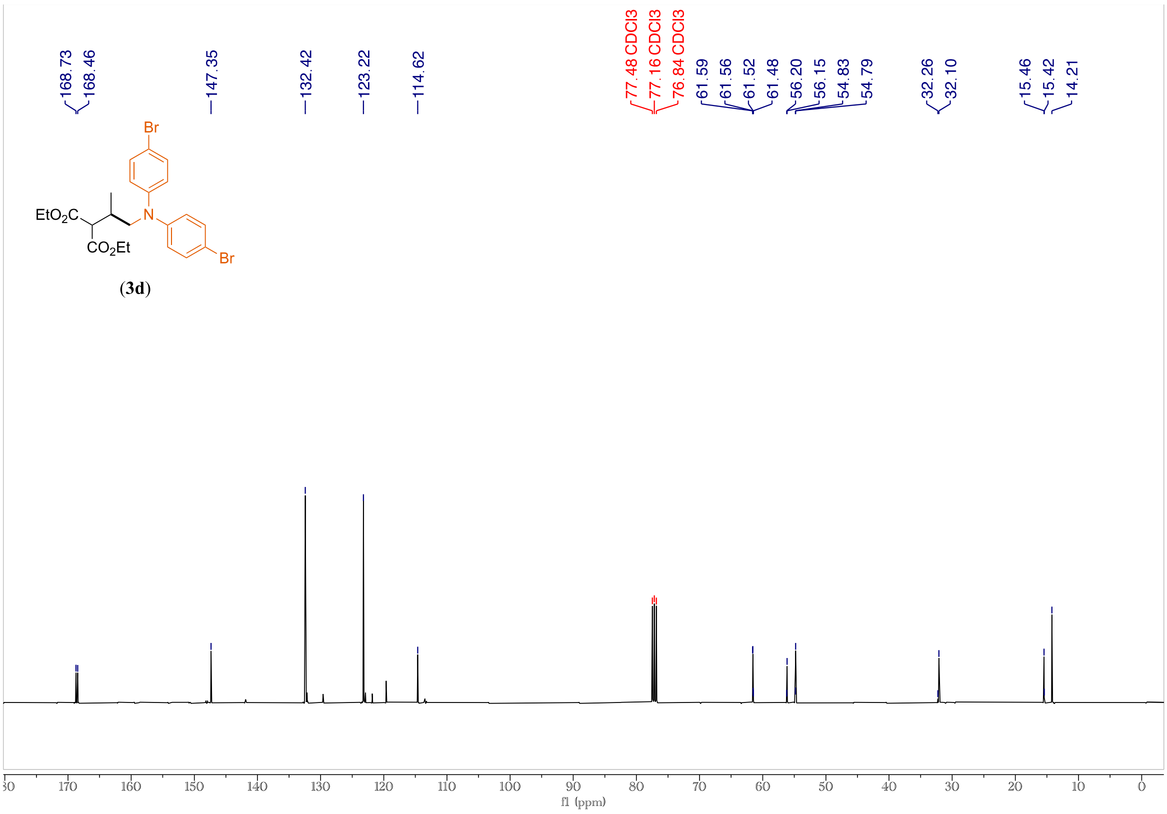

Figure S42: ^{13}C NMR spectrum (101 MHz) of (**3d**) in CDCl3.

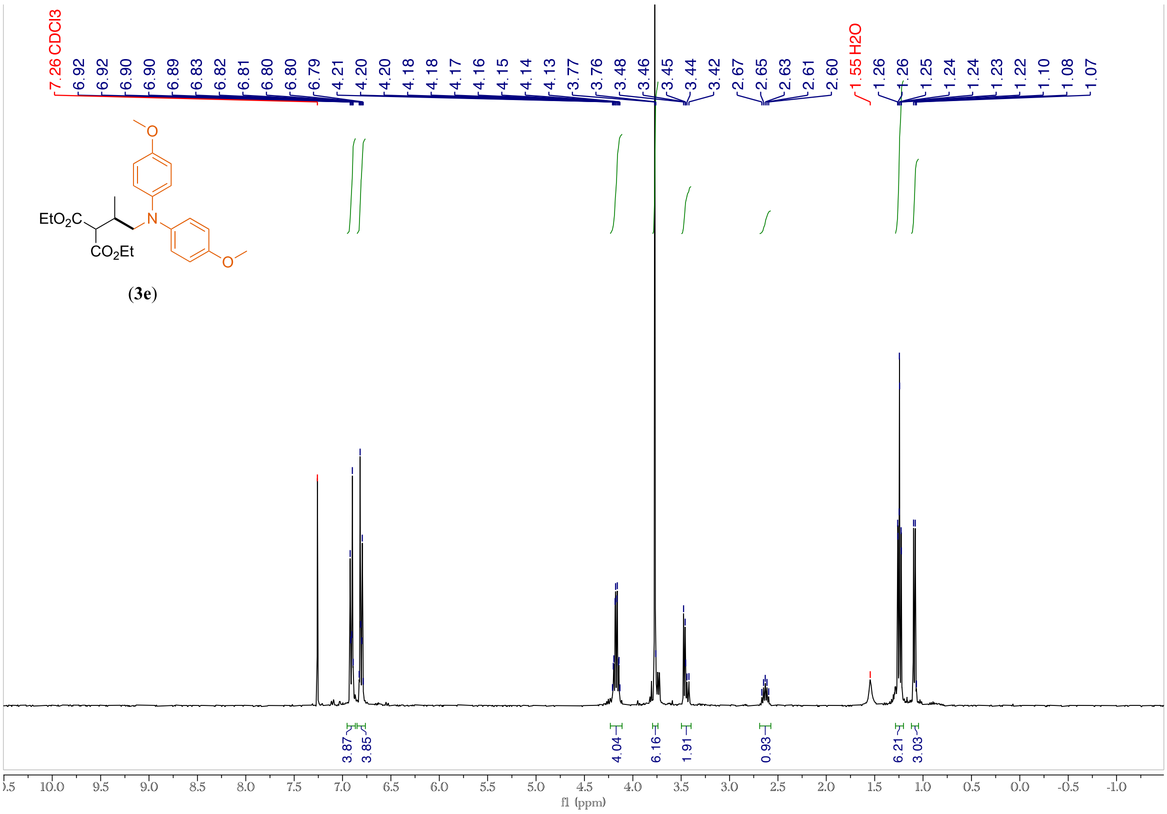

Figure S43: ^{1}H NMR spectrum (400 MHz) of **(3e)** in $CDCl_3$.

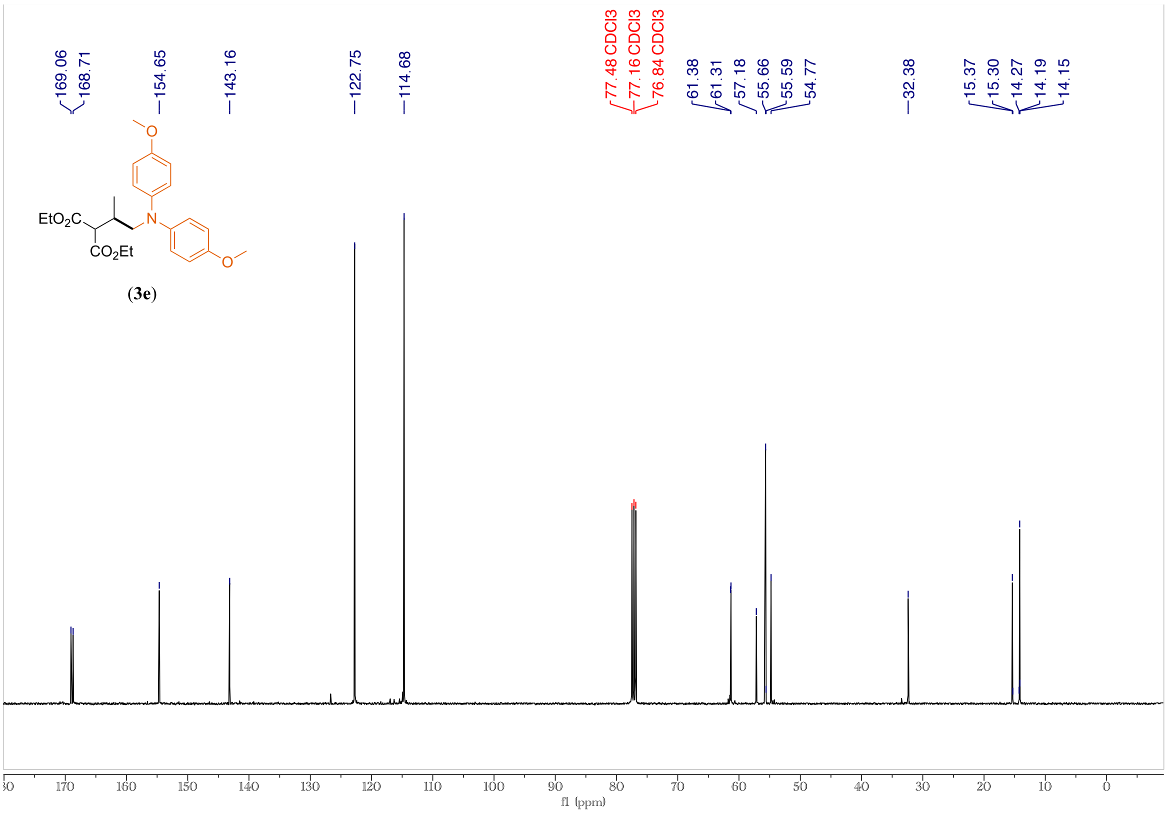

Figure S44: ^{13}C NMR spectrum (101 MHz) of (**3e**) in CDCl3.

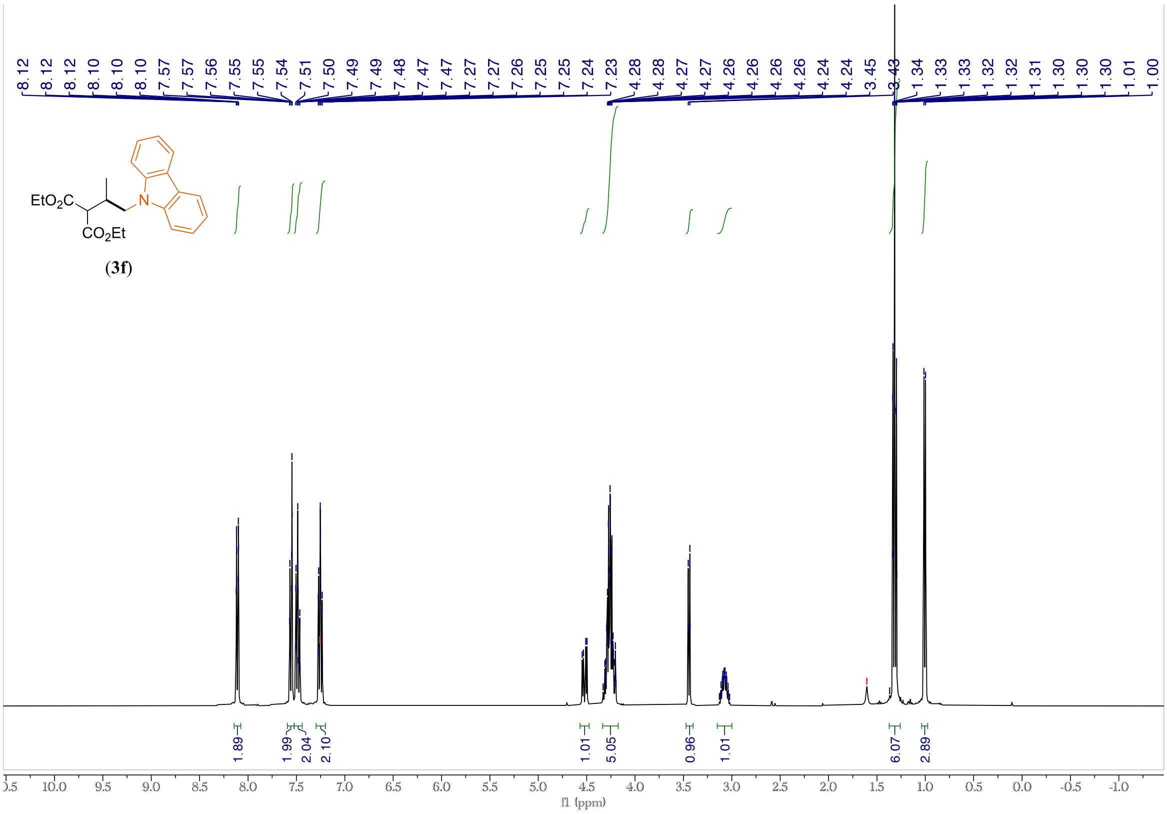

Figure S45: ^{1}H NMR spectrum (400 MHz) of **(3f)** in $CDCl_3$.

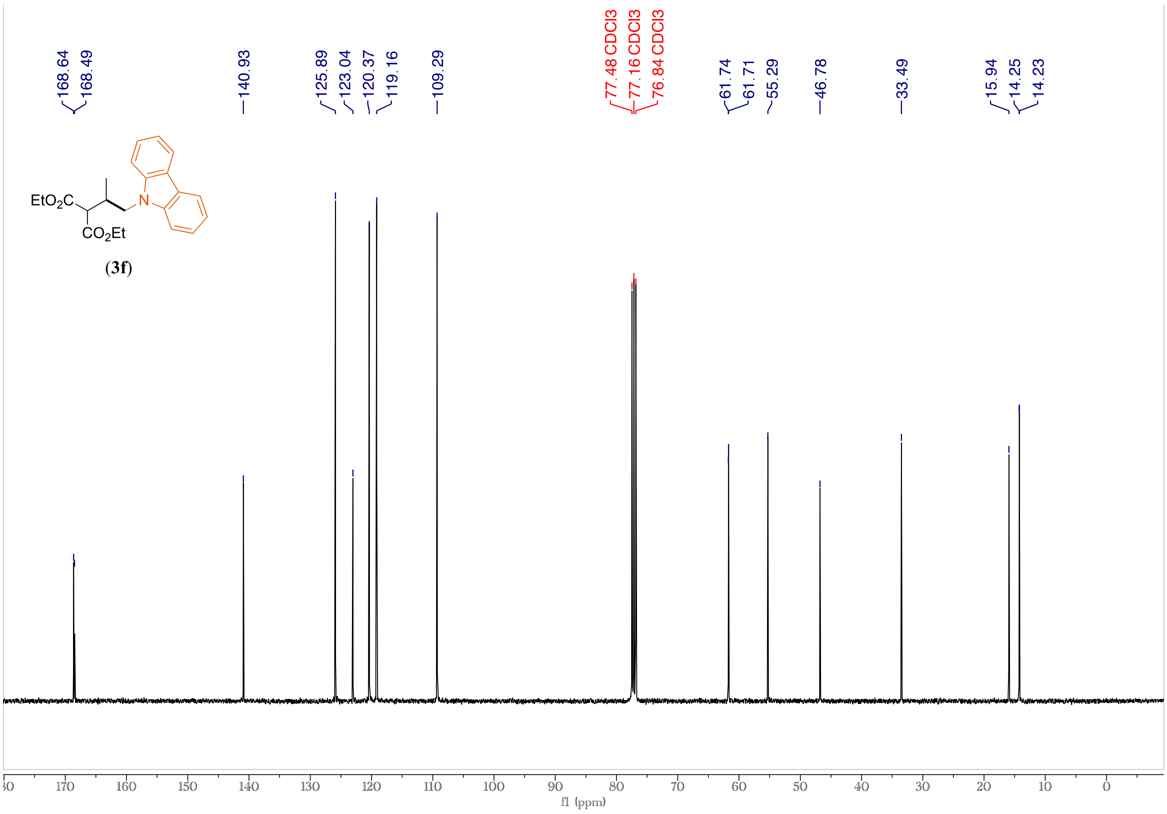

Figure S46: ^{13}C NMR spectrum (101 MHz) of (**3f**) in CDCl3.

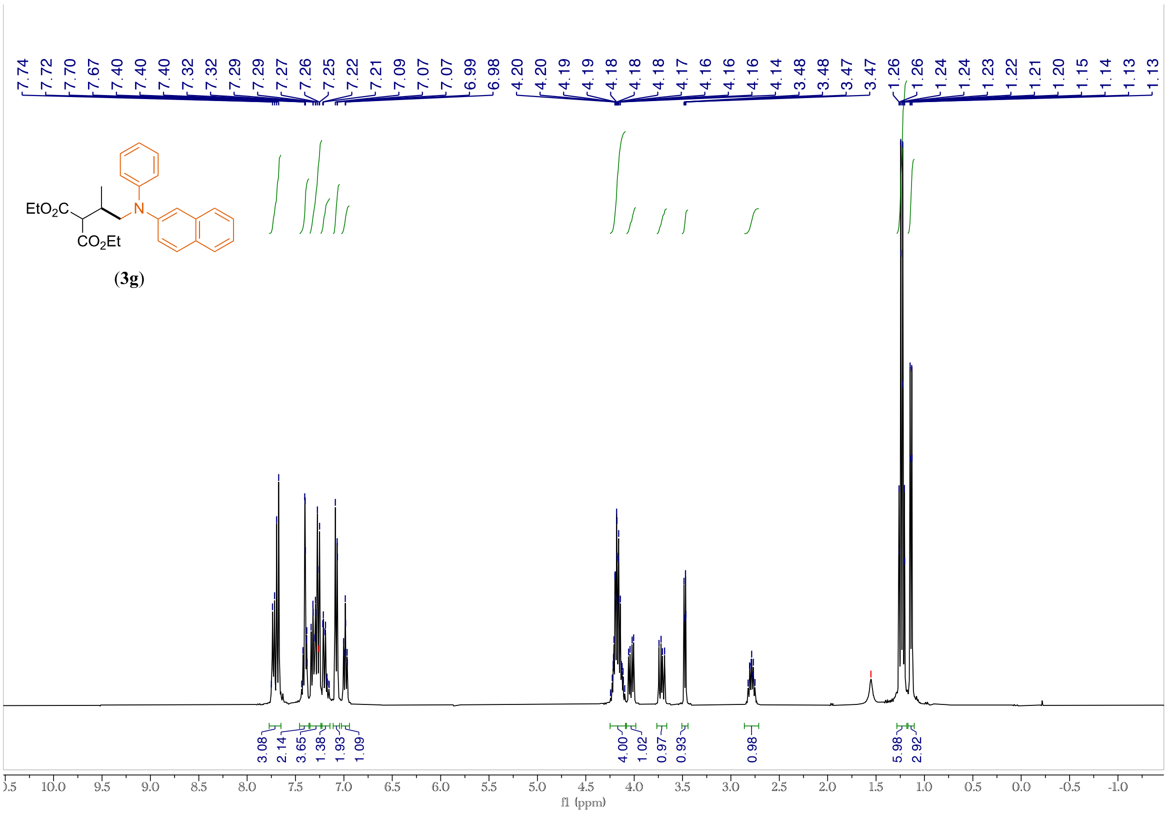

Figure S47: ^{1}H NMR spectrum (400 MHz) of **(3g)** in $CDCl_3$.

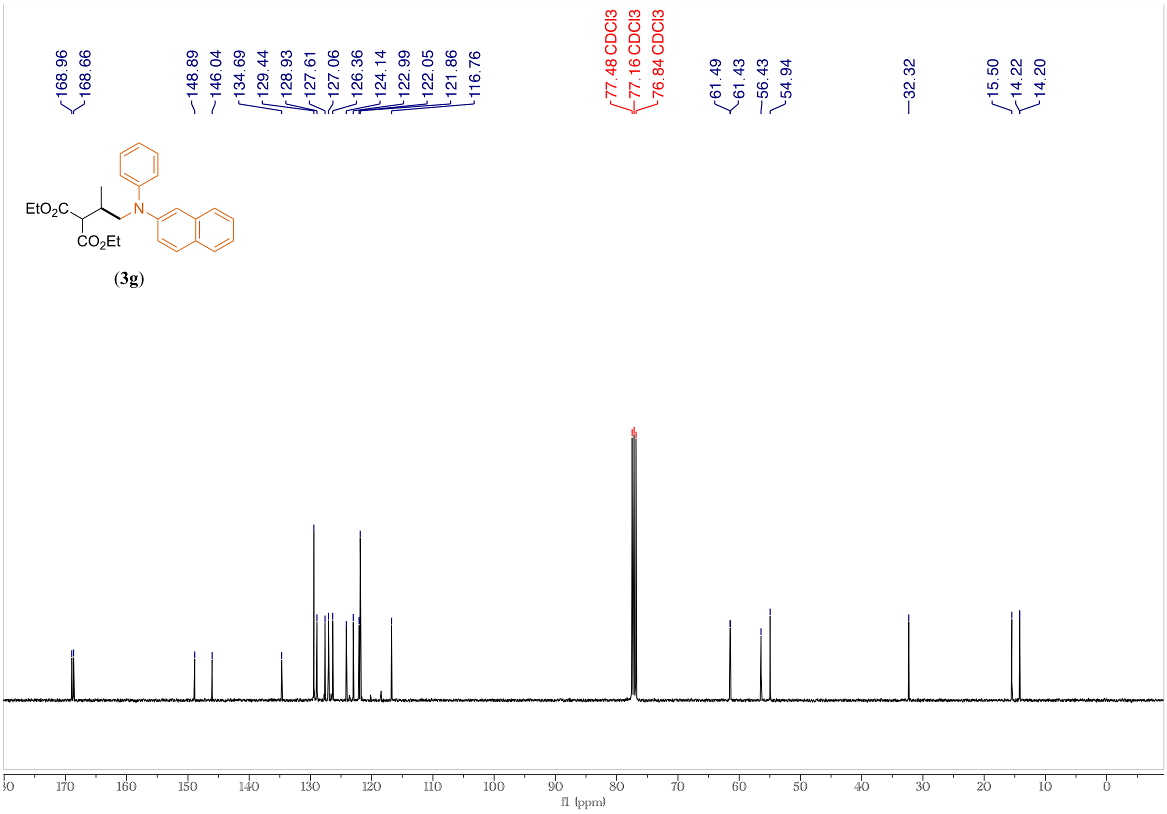

Figure S48: ^{13}C NMR spectrum (101 MHz) of (**3g**) in CDCl3.

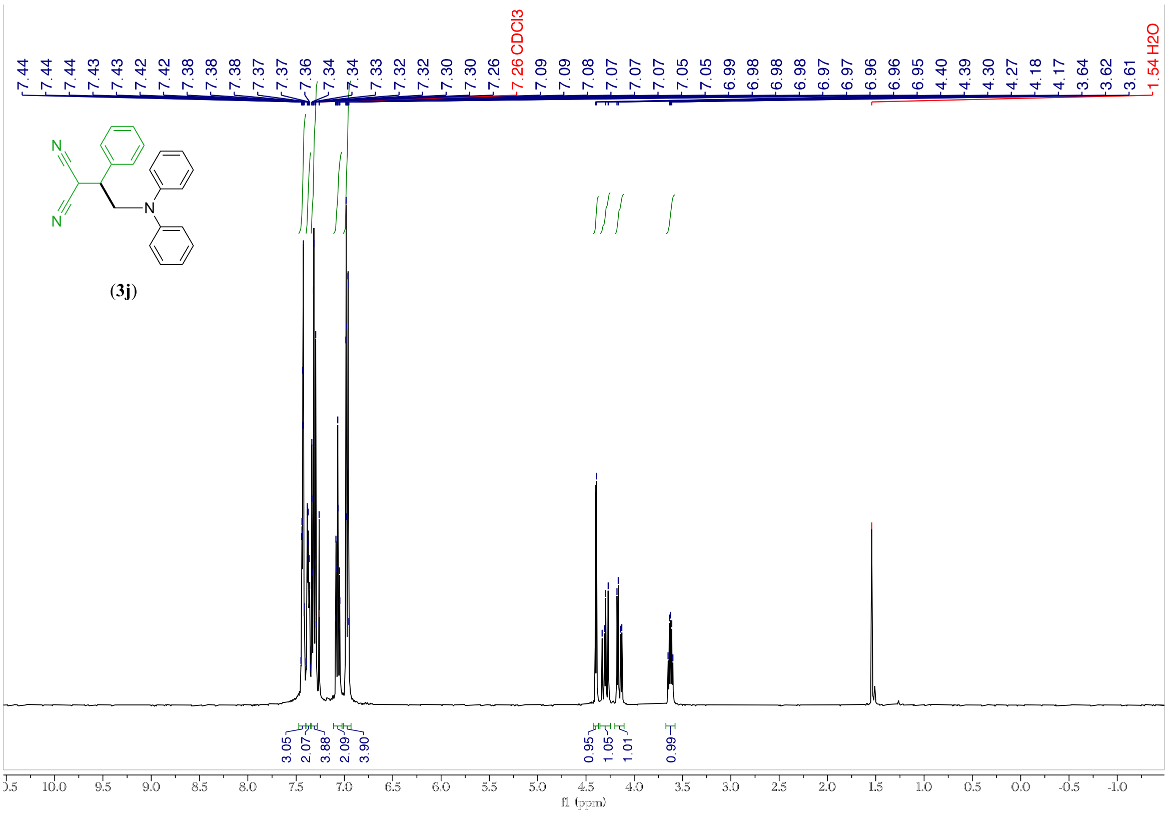

Figure S49: ^{1}H NMR spectrum (400 MHz) of **(3j)** in $CDCl_3$.

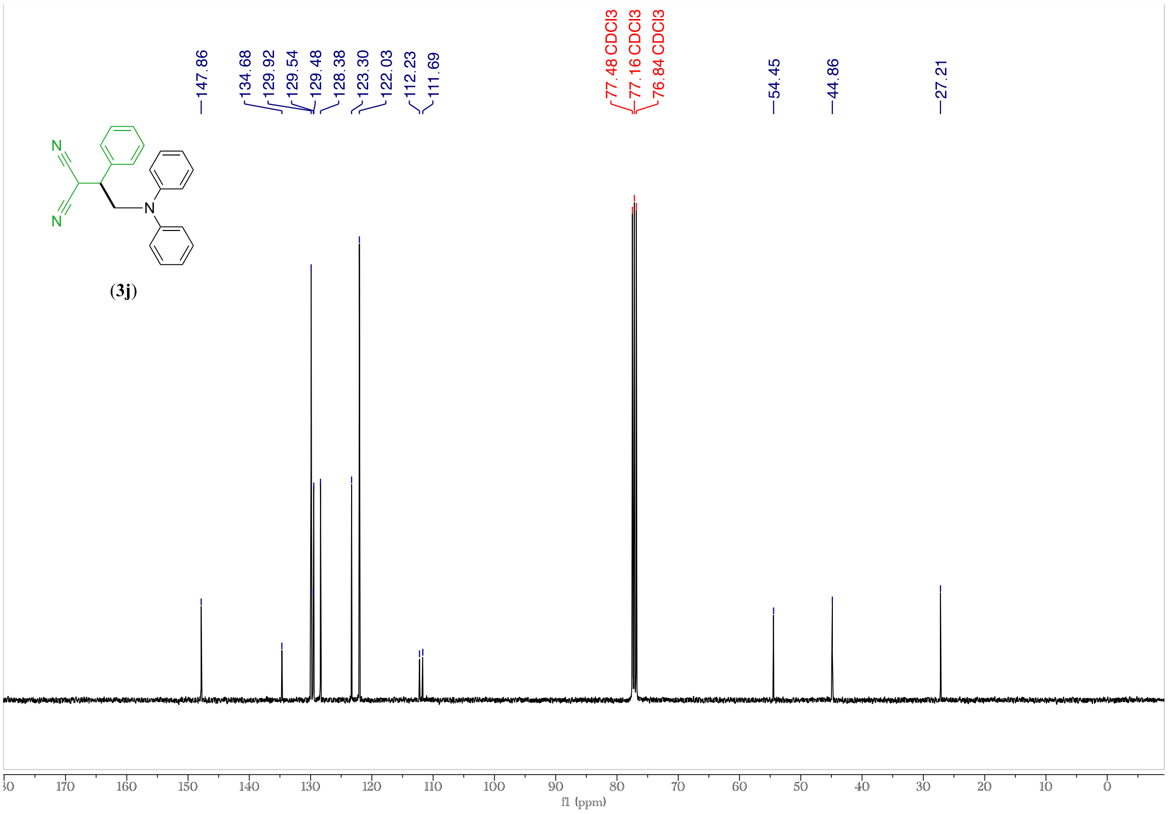

Figure S50: ^{13}C NMR spectrum (101 MHz) of (**3j**) in CDCl3.

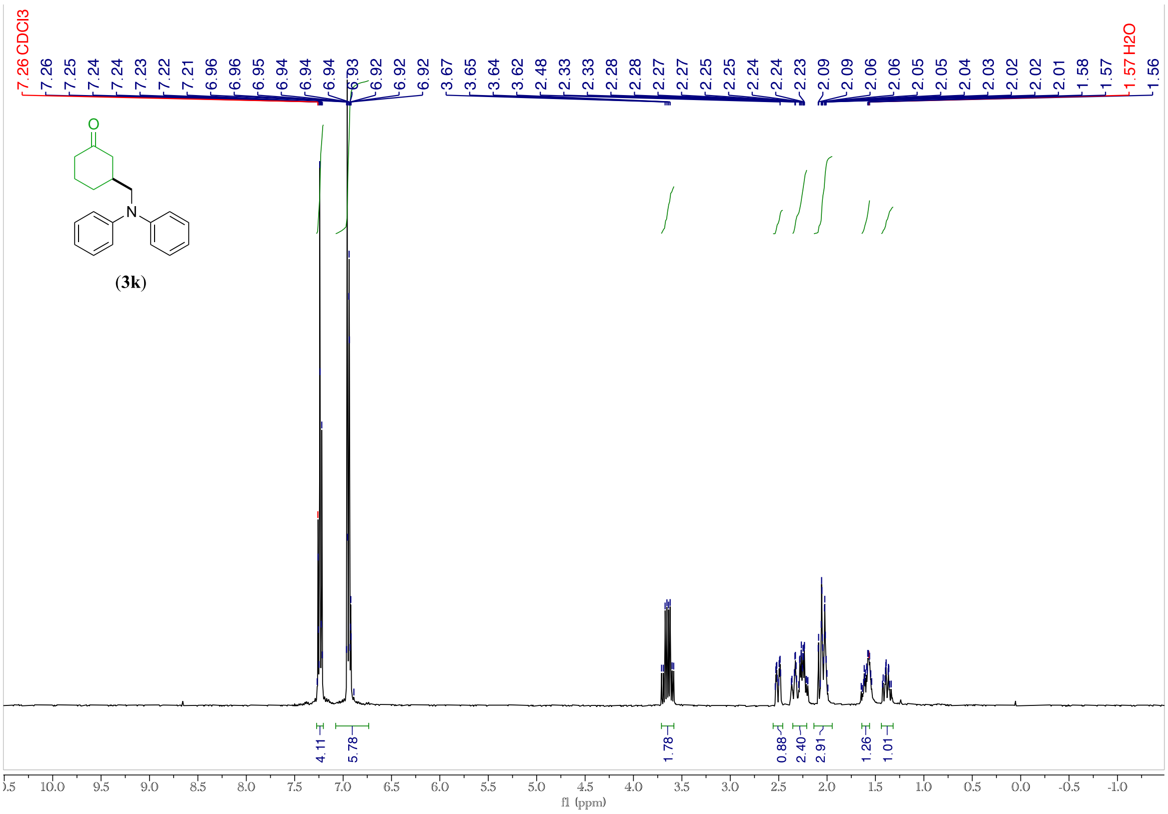

Figure S51: ^{1}H NMR spectrum (400 MHz) of **(3k)** in $CDCl_3$.

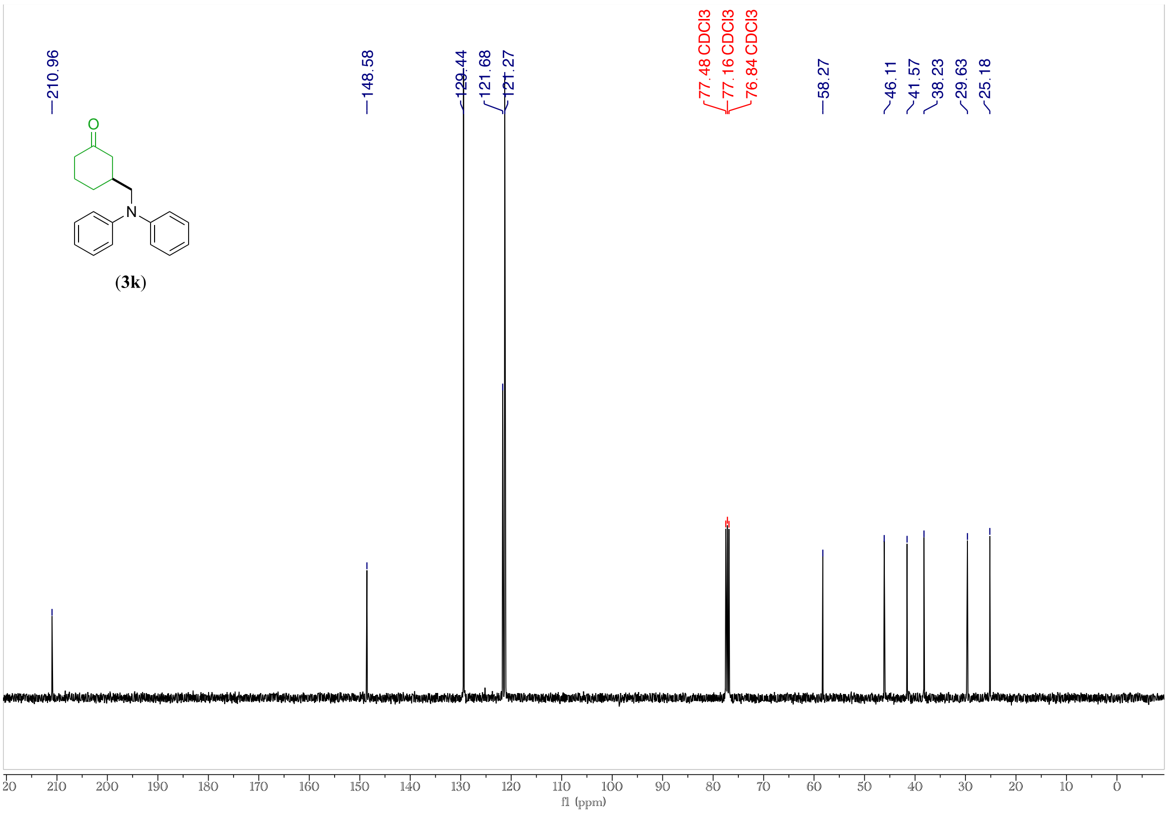

Figure S52: ^{13}C NMR spectrum (101 MHz) of (**3k**) in CDCl3.

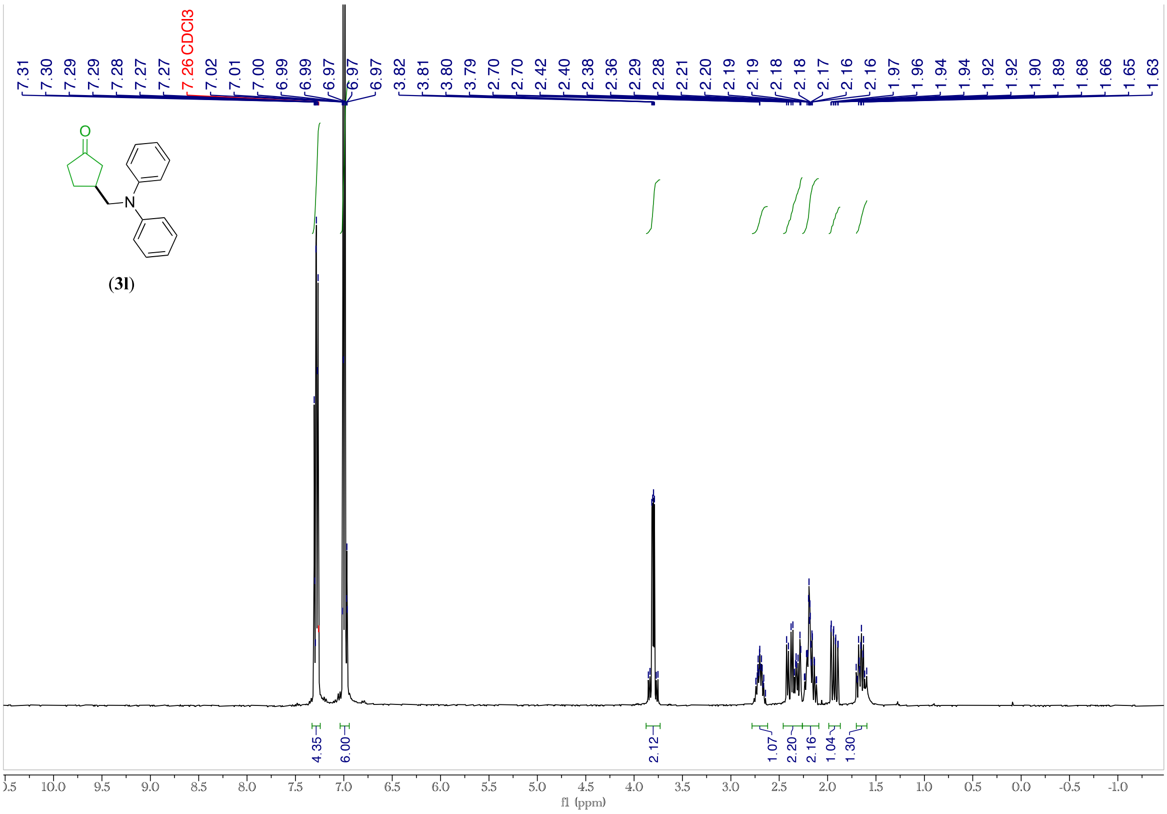

Figure S53: ^{1}H NMR spectrum (400 MHz) of **(3l)** in $CDCl_3$.

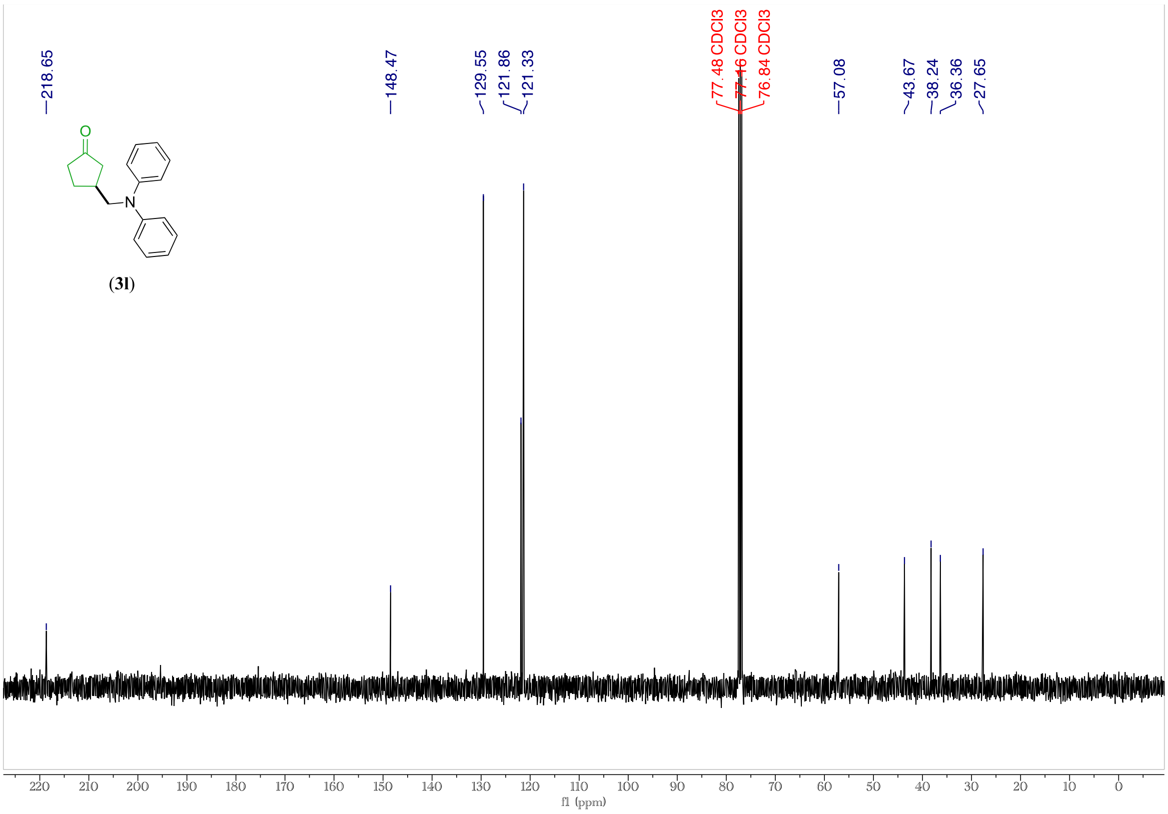

Figure S54: ^{13}C NMR spectrum (101 MHz) of (**3l**) in CDCl3.

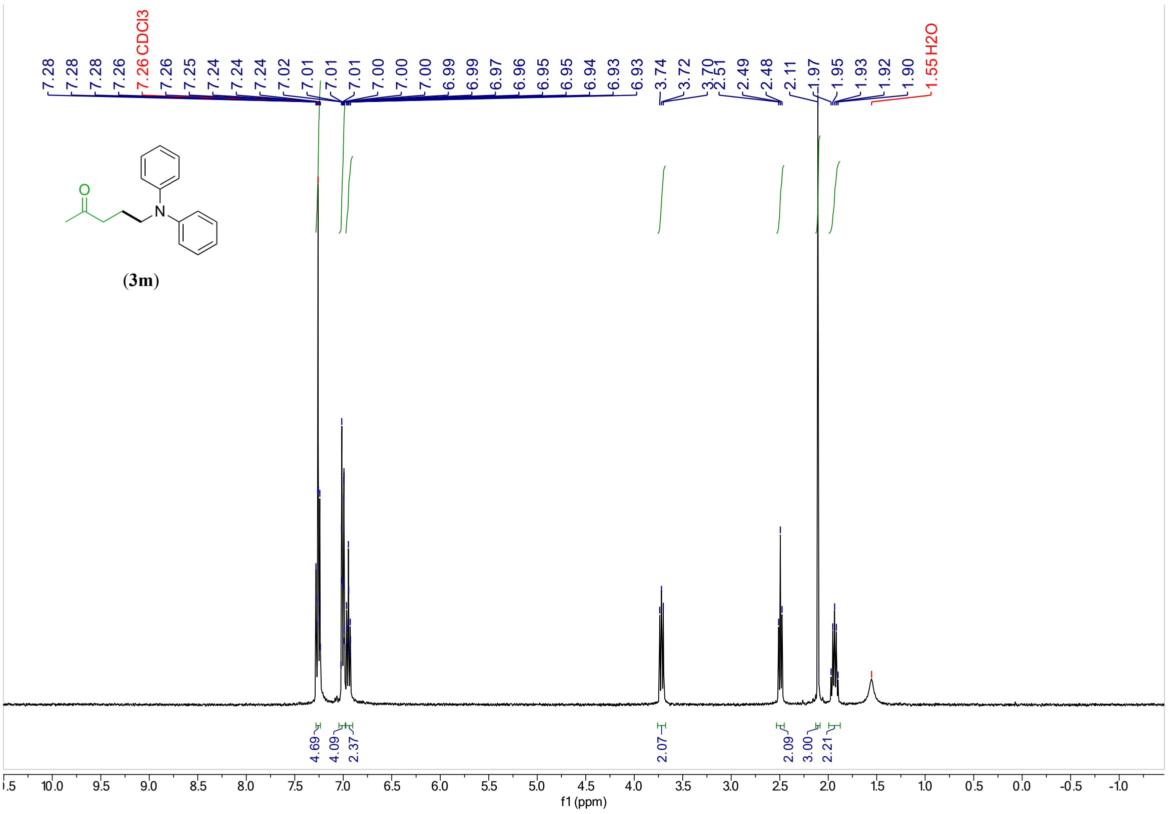

Figure S55: ^{1}H NMR spectrum (400 MHz) of **(3m)** in $CDCl_3$.

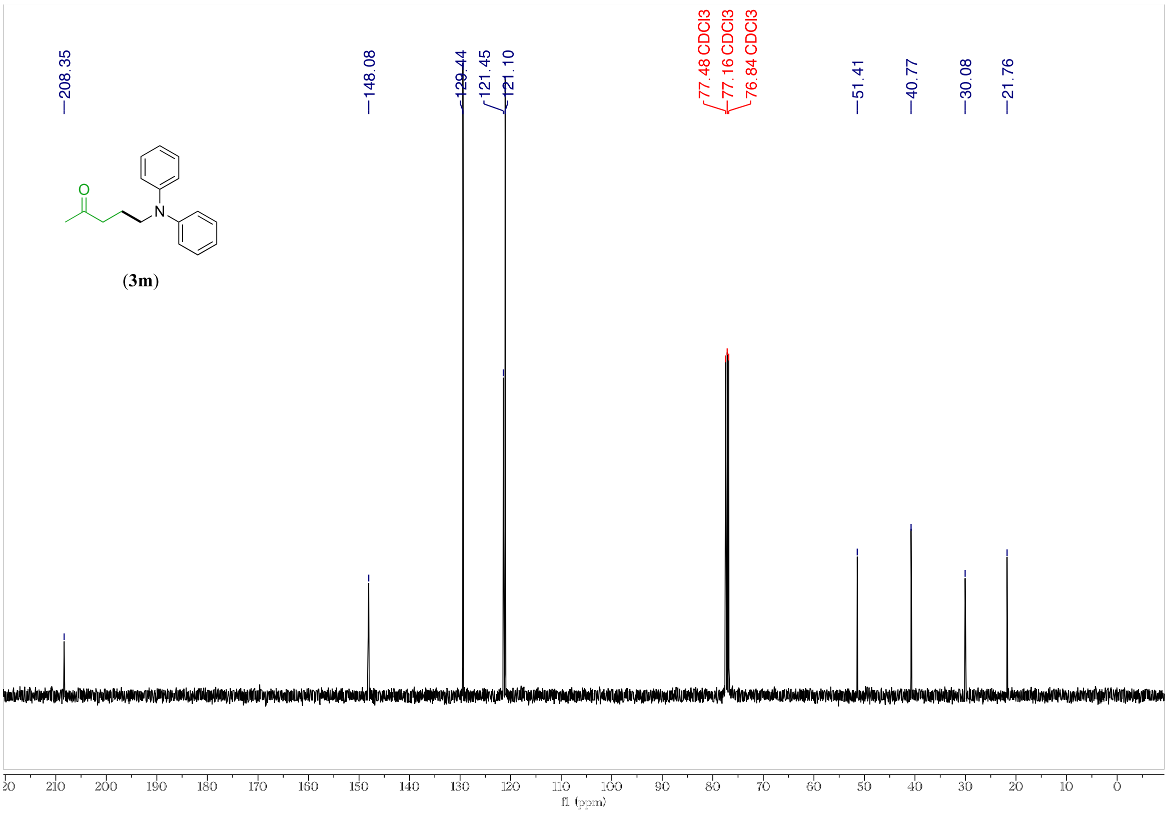

Figure S56: ^{13}C NMR spectrum (101 MHz) of (**3m**) in CDCl3.

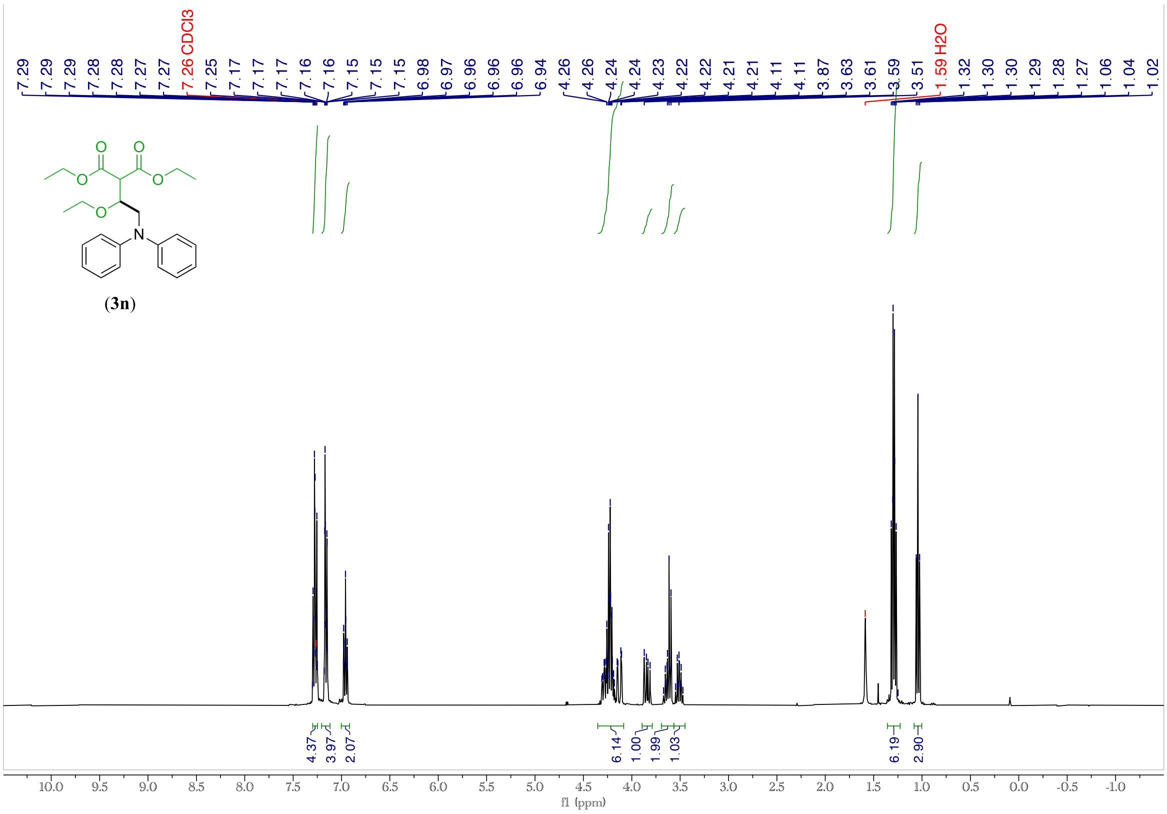

Figure S57: ^{1}H NMR spectrum (400 MHz) of **(3n)** in $CDCl_3$.

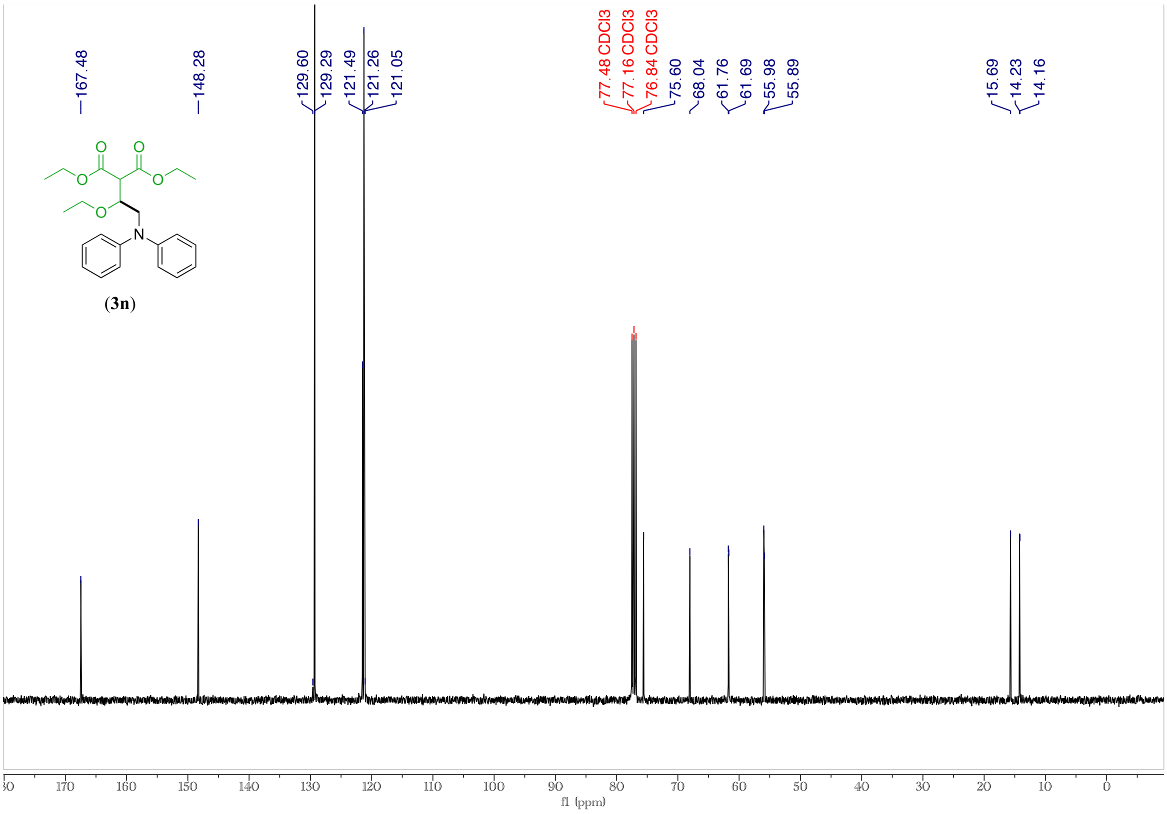

Figure S58: ^{13}C NMR spectrum (101 MHz) of (**3n**) in CDCl3.

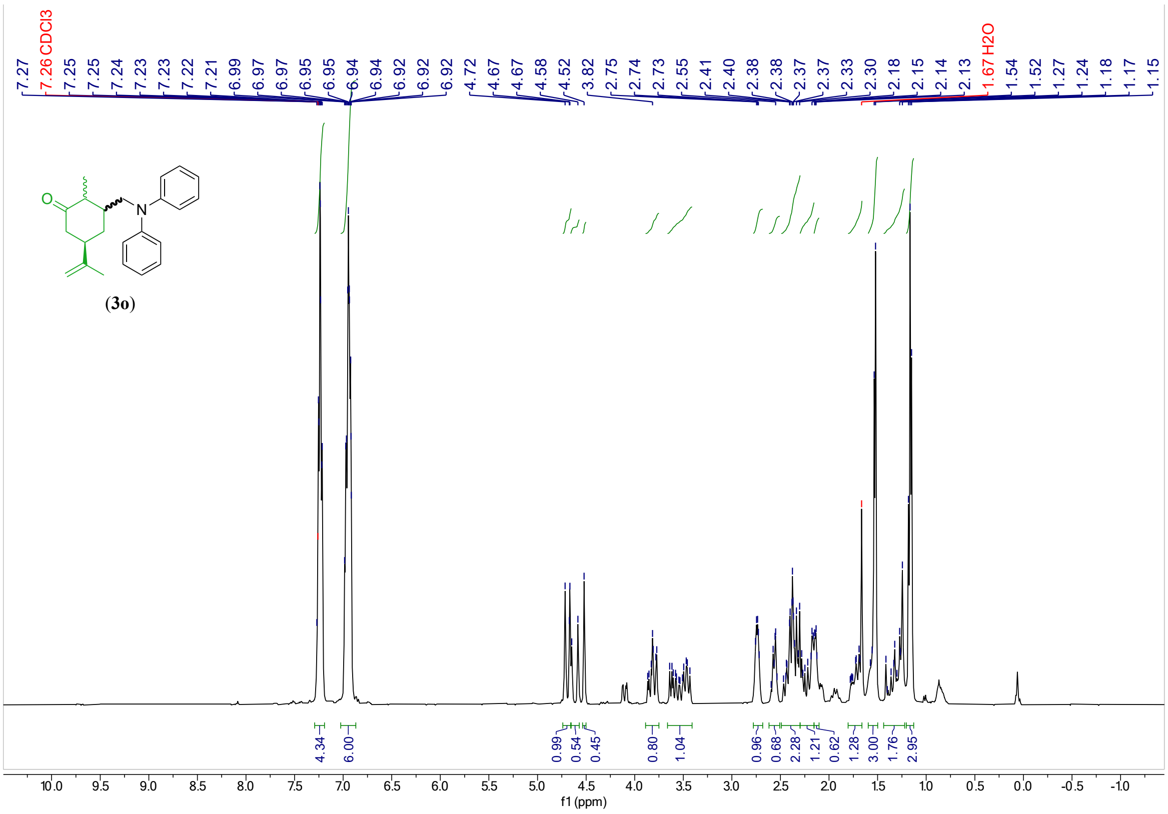

Figure S59: ^{1}H NMR spectrum (400 MHz) of **(3o)** in $CDCl_3$.

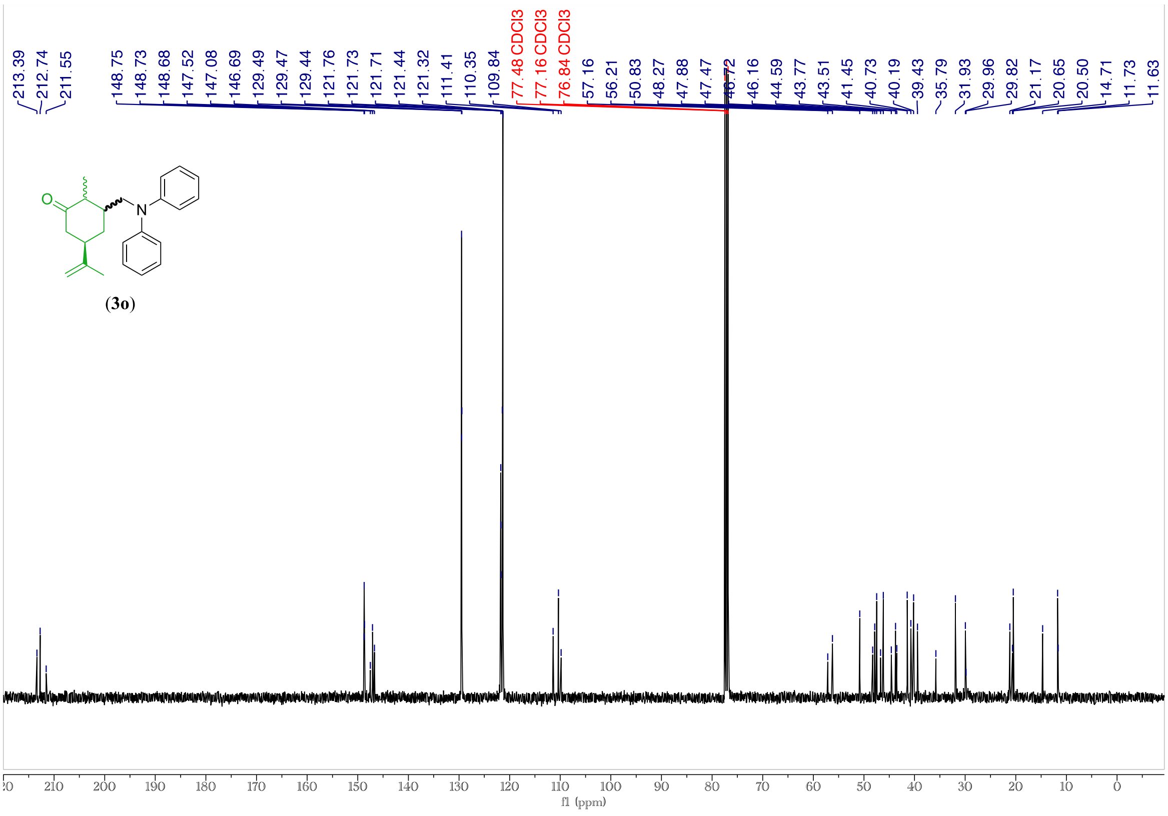

Figure S60: ^{13}C NMR spectrum (101 MHz) of (**3o**) in CDCl3.

References

1. K. S. Kjaer, N. Kaul, O. Prakash, P. Chábera, N. W. Rosemann, A. Honarfar, O. Gordivska, L. A. Fredin, K. E. Bergquist, L. Häggström, T. Ericsson, L. Lindh, A. Yartsev, S. Styring, P. Huang, J. Uhlig, J. Bendix, D. Strand, V. Sundström, P. Persson, R. Lomoth and K. Wärnmark, *Science*, 2019, **363**, 249-253.
2. P. Chábera, Y. Liu, O. Prakash, E. Thyrhaug, A. E. Nahhas, A. Honarfar, S. Essen, L. A. Fredin, T. C. Harlang, K. S. Kjaer, K. Handrup, F. Ericson, H. Tatsuno, K. Morgan, J. Schnadt, L. Häggström, T. Ericsson, A. Sobkowiak, S. Lidin, P. Huang, S. Styring, J. Uhlig, J. Bendix, R. Lomoth, V. Sundström, P. Persson and K. Wärnmark, *Nature*, 2017, **543**, 695-699.
3. J. Zhang, D. Campolo, F. Dumur, P. Xiao, J. P. Fouassier, D. Gigmes and J. Lalevée, *J. Polym. Sci. Part A: Polym. Chem.*, 2016, **54**, 2247-2253.
4. S. F. Nelsen and J. T. Ippoliti, *J. Am. Chem. Soc.*, 2002, **108**, 4879-4881.
5. X. Zhang, S.-R. Yeh, S. Hong, M. Freccero, A. Albini, D. E. Falvey and P. S. Mariano, *J. Am. Chem. Soc.*, 2002, **116**, 4211-4220.
6. C. Wang, Y. Zheng, H. Huo, P. Röse, L. Zhang, K. Harms, G. Hilt and E. Meggers, *Chem. - Eur. J.*, 2015, **21**, 7355-7359.
7. J. L. Schwarz, R. Kleinmans, T. O. Paulisch and F. Glorius, *J. Am. Chem. Soc.*, 2020, **142**, 2168-2174.
8. S. Zheng, Z. Chen, Y. Hu, X. Xi, Z. Liao, W. Li and W. Yuan, *Angew. Chem. Int. Ed.*, 2020, **59**, 17910-17916.
9. P. Müller and K. Brettel, *Photochem. Photobiol. Sci.*, 2012, **11**, 632-636.
